# A single genetic locus controls both expression of *DPEP1/CHMP1A* and kidney disease development via ferroptosis

**DOI:** 10.1038/s41467-021-25377-x

**Published:** 2021-08-23

**Authors:** Yuting Guan, Xiujie Liang, Ziyuan Ma, Hailong Hu, Hongbo Liu, Zhen Miao, Andreas Linkermann, Jacklyn N. Hellwege, Benjamin F. Voight, Katalin Susztak

**Affiliations:** 1 Department of Medicine, Renal Electrolyte and Hypertension Division, Perelman School of Medicine, University of Pennsylvania, Philadelphia, PA 19104 USA; 2 Department of Genetics, Perelman School of Medicine, University of Pennsylvania, Philadelphia, PA 19104 USA; 3 Graduate group in Genomics and Computational Biology, Perelman School of Medicine, University of Pennsylvania, Philadelphia, PA 19104 USA; 4 Division of Nephrology, Department of Internal Medicine, University Hospital Carl Gustav Carus at the Technische Universität Dresden, Dresden, Germany; 5 Biotechnology Center, Technische Universität Dresden, 01307 Dresden, Germany; 6 Division of Genetic Medicine, Department of Medicine, Vanderbilt Genetics Institute, Nashville, TN 37232 USA; 7 Department of Systems Pharmacology and Translational Therapeutics, Perelman School of Medicine, University of Pennsylvania, Philadelphia, PA 19104 USA; 8 Institute of Translational Medicine and Therapeutics, Perelman School of Medicine, University of Pennsylvania, Philadelphia, PA 19104 USA

**Keywords:** Genetics, Functional genomics, Kidney diseases

## Abstract

Genome-wide association studies (GWAS) have identified loci for kidney disease, but the causal variants, genes, and pathways remain unknown. Here we identify two kidney disease genes Dipeptidase 1 (*DPEP1*) and Charged Multivesicular Body Protein 1 A (*CHMP1A*) via the triangulation of kidney function GWAS, human kidney expression, and methylation quantitative trait loci. Using single-cell chromatin accessibility and genome editing, we fine map the region that controls the expression of both genes. Mouse genetic models demonstrate the causal roles of both genes in kidney disease. Cellular studies indicate that both *Dpep1* and *Chmp1a* are important regulators of a single pathway, ferroptosis and lead to kidney disease development via altering cellular iron trafficking.

## Abstract (web summary)

Identifying causal variants and genes is an essential step in interpreting GWAS loci. Here, the authors investigate a kidney disease GWAS locus with functional genomics data, CRISPR editing and mouse experiments to identify DPEP1 and CHMP1A as putative kidney disease genes via ferroptosis.

## Introduction

Chronic kidney disease (CKD) affects 800 million people worldwide and remains the tenth leading cause of death. Key molecular pathways that govern CKD pathogenesis remain largely unknown. Disease susceptibility shows substantial heterogeneity^1^, which is thought to be explained by environmental and genetic risk factors that have yet to be fully elucidated.

Genome-wide association analyses (GWAS) performed in large populations identified nearly 250 loci where genetic variants associated with kidney function^2–4^. Despite the success of locus discovery, the causal variants, target genes, and cell types along with the underlying molecular mechanisms remain unresolved, mostly because more than 90% of GWAS identified variants are non-coding^5^ and the variants are linked due to strong linkage disequilibrium^6^.

Prior GWAS functional annotation studies have highlighted the enrichment of the causal variants in cell type-specific cis-regulatory regions^7,8^. Epigenetic annotation has helped to narrow variants with the highest probability of regulatory function^9^. One way in which a GWAS variant could influence the nearby gene is by altering the sequence where transcription factors (TFs) bind^10^. With this simple model in mind, contemporary approaches to identify a potential target gene have relied on the expression of quantitative trait loci analysis (eQTL), where genetic variants and gene expression changes are correlated in a tissue-specific manner^11^.

Due to the strong linkage disequilibrium multiple variants at a single locus show strong association with disease development. Initial locus dissection studies focused on defining a single causal variant responsible for disease development. New experimental locus dissection studies indicate that it is possible that multiple SNPs at a single locus is responsible for gene regulation. Furthermore, it is also tempting to speculate that multiple GWAS causal variants regionally implicate the same causal gene, this may not be true in all cases. In addition, genes are not randomly distributed in the genome. Genes with similar functions are often clustered together^12^, such genic ‘operons’ may share regulatory regions in common, raising the possibility that multiple genes within an individual locus contribute causally to the underlying phenotype. The extent to which multiple phenotype-causal genes are driven by a single or multiple causal variant at a single locus is not fully known.

In this study, we computationally integrated kidney function GWAS, methylation, expression quantitative trait loci, and human kidney single nuclei ATAC sequencing studies. Our analysis prioritized both *DPEP1* and *CHMP1A* as kidney disease risk genes in kidney proximal tubules, likely mediating the effect of the eGFR GWAS signal observed on chromosome 16. Using CRISPR mediated genome deletion, we showed that both genes were regulated by a common genomic locus in human kidney proximal tubule cells. Mouse gene knockouts highlighted the key role of both genes in kidney disease development. Molecular studies indicated that both genes are regulators of ferroptosis.

## Results

### Shared causal variants for kidney function and *CHMP1A*/*DPEP1* expression

**Fig. 1:**
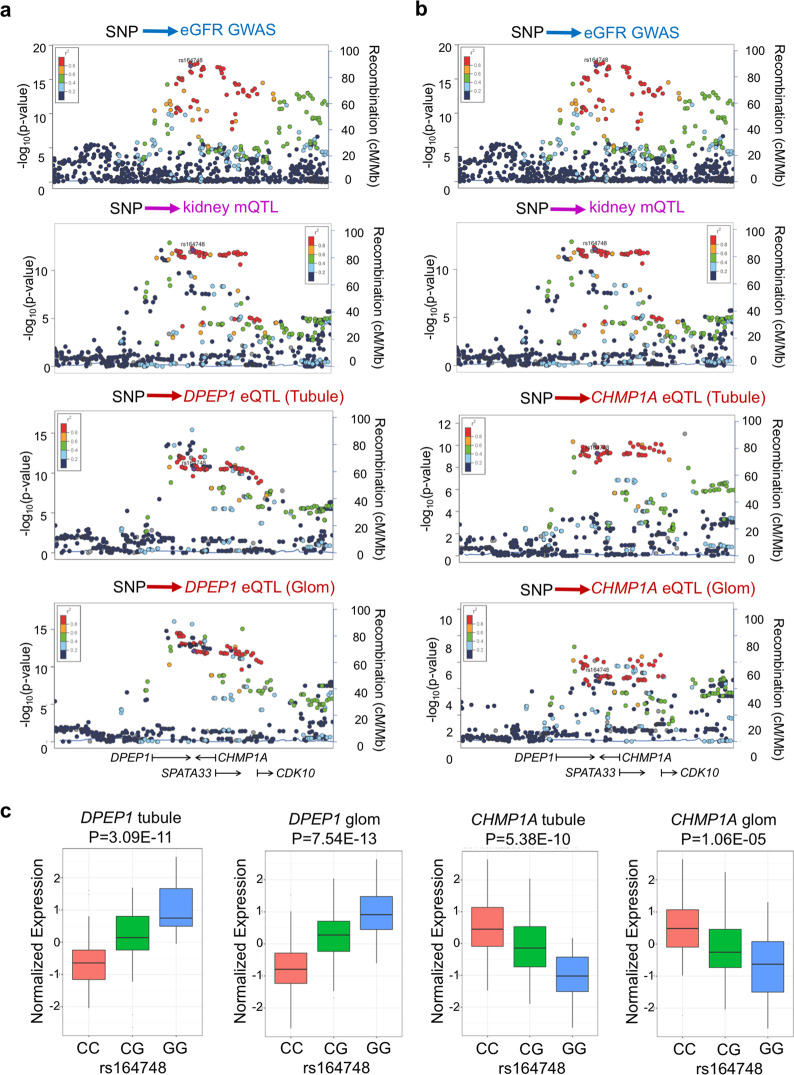
Shared genetic variants associated with kidney function, human kidney methylation, and *DPEP1*/*CHMP1A* expression. **a** LocusZoom plots of eGFR GWAS, human kidney mQTL analysis (genotype-methylation, *n* = 188), eQTLs (genotype-expression of *DPEP1*) in kidney compartments (tubule *n* = 121 or glomerulus *n* = 119). **b** LocusZoom plots of eGFR GWAS, human kidney mQTL analysis (genotype-methylation, *n* = 188), eQTLs (genotype-expression of *CHMP1A*) in kidney compartments (tubule *n* = 121 or glomerulus *n* = 119). The x-axis indicates the genomic location on chromosome 16. The arrow indicates the transcriptional direction for specific genes. Each dot represent one SNP. The dots are colored according to their correlation to the index SNP (rs164748). The red dots indicates strong correlation (*r*^*2*^ > 0.8) (LD) with the index SNP. The left y-axis indicates −log_10_ (*P* value). The right y-axis indicates recombination rate (cM/Mb). **c** Genotype (rs164748) and gene expression (*DPEP1* and *CHMP1A*) association in human tubules (*n* = 121) and glomeruli (*n* = 119) in the Susztak lab database^40^. The effect size estimate (Beta) and standard error (SE) are as below: *DPEP1* tubule Beta = 0.811 and SE = 0.11; *DPEP1* glom Beta = 0.889 and SE = 0.11; *CHMP1A* tubule Beta = −0.766 and SE = 0.113; *CHMP1A* glom Beta = −0.587 and SE = 0.127. Centerlines show the medians; box limits indicate the 25th and 75th percentiles; whiskers extend to the 5th and 95th percentiles. *P* value was calculated as previously published^40^.

Previous GWAS have demonstrated robust and reproducible association of the chromosome 16 region tagged by rs164748^3,13,14^ with kidney function (eGFR) (Fig. 1a and Fig. S1a). This locus spans multiple genes, which include *DPEP1*, *CHMP1A, SPATA33*, and *CDK10*. To dissect this region, we integrated the GWAS data with kidney methylation quantitative trait loci and (mQTL) and eQTL, respectively. First, we observed an association between nucleotide variants at this region and cytosine methylation level in healthy human kidney samples (*n* = 188) (Fig. 1a, b). Furthermore, human kidney eQTL information demonstrated an association between variants at this region and the expression of *DPEP1* and *CHMP1A* (Fig. 1a, b). This eQTL effect was detected both in the tubule (*n* = 121) and glomerular (*n* = 119) compartments.

As both mQTL and eQTLs appeared to be tagged by rs164748, we next performed statistical colocalization to quantify the extent to which causal genetic variants for kidney function, methylation, and gene expression were shared. Moloc analysis was performed with a+/− 100 kb window, covering most cis-regulatory interactions. Molocalization analyses suggested a high posterior probability that the eGFR GWAS, mQTL, and eQTL could be explained by common variants (PP_abc = 0.92, “Methods” section). The moloc identified CpGs (cg21038338, cg11907426, cg07716468, cg01510696, and cg00487989) were kidney-specific mQTLs (when compared to whole blood^15^ and skeletal muscle)^16^ (Supplementary Data 1). By examining the association between GWAS variants and gene expression in a collection of microdissected human kidney tubule and glomerulus samples, we found that the genotype of the top GWAS eGFR SNP (rs164748) strongly influenced the level of *DPEP1* both in glomeruli and tubuli. Specifically, the GWAS risk allele G was associated with increased *DPEP1* expression. Moreover, we found that the genotype of this top GWAS eGFR SNP (rs164748) strongly influenced the expression level of *CHMP1A* in tubuli. The GWAS risk allele G was associated with lower *CHMP1A* expression (Fig. 1c). We noted that the tubule and glomerular eQTL effects at rs164748 on *DPEP1* and the glomerular eQTL effect on *CHMP1A* replicated in the publicly available NephQTL database^17^, which contained only 136 samples (Fig. S1b). In the GWAS, the C allele of rs164748 was associated with high eGFR, while in the eQTL, the C allele was associated with lower *DPEP1* and higher *CHMP1A* expression.

We next examined the potential eQTL effects of rs164748 in the GTEx project^18^. While we did note an association between the CKD risk genotype and *CHMP1A* expression in skin samples (Supplementary Data 2), we did not observe an association between genetic variation of rs164748 and expression of *DPEP1* in any GTEx tissue (Supplementary Data 2). We also did not observe an association between rs164748 genotype and expression of *CDK10* and *SPATA33* in kidney samples (Fig. S1b–d). Collectively, these results suggest a kidney-specific mQTL/eQTL effect of rs164748, which prioritized both *DPEP1* and *CHMP1A* as kidney disease risk genes.

### Human kidney-specific single nuclei chromatin accessibility analysis highlights likely causal variants in kidney proximal tubules

**Fig. 2:**
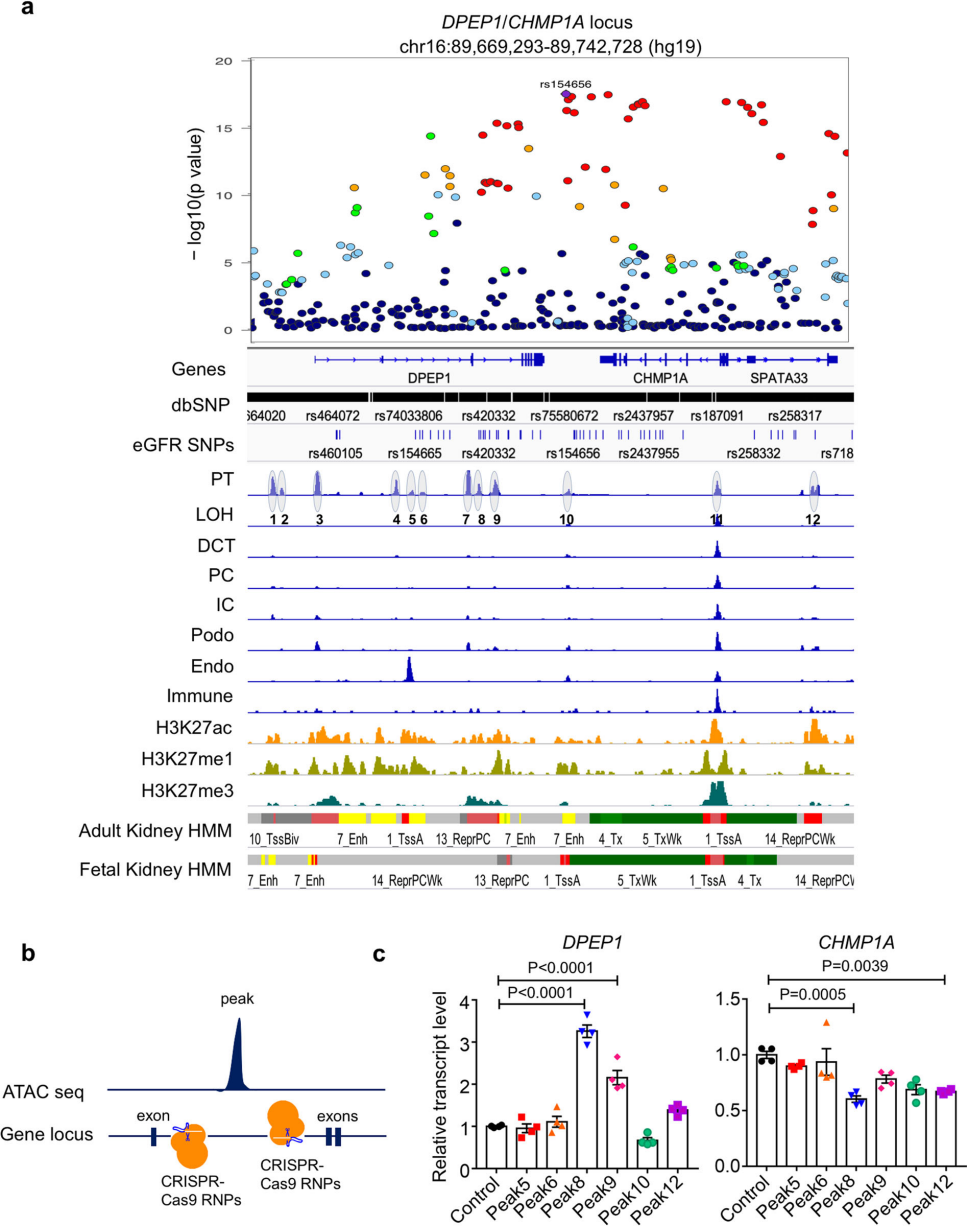
Fine mapping of the GWAS locus via human kidney single nuclei ATAC-seq analysis coupled with genome editing. **a** From top to bottom: locuszoom plots of eGFR GWAS; Gene browser view of the single nucleotide polymorphisms within the regions; genome browser view of chromatin accessibility for proximal tubules (PT), loop of Henle (LOH), distal convoluted tubule (DCT), collecting duct principal cell types (PC), collecting duct intercalated cells (IC), podocytes (Podo), endothelial cells (Endo), immune cells (Immune); genome browser view of whole kidney H3K27ac, H3K4me1, and H3K4me3 histone ChIP-seq; ChomHMM annotation human adult and fetal kidneys. Proximal tubule-specific open chromatin region across this region was circled and numbered. **b** Schematic of CRISPR/Cas9 mediated open chromatin region deletion. **c** Relative transcript levels of *DPEP1* and *CHMP1A* following open chromatin region deletion (*n* = 4). All data are represented as mean ± SEM. *P* value was calculated by one-way ANOVA with post hoc Tukey test. *P* < 0.05 is statistically significant. A Source Data file is available for this figure.

To narrow likely causal variants, we utilized human kidney single nuclei sequencing assay for transposase-accessible chromatin (snATAC-seq). Interrogation of the snATAC-Seq data at this locus identified 12 accessible chromatin regions. One accessible chromatin region, nearby the 5′ end of *CHMP1A*, was present in all analyzed cells, likely corresponding to the promoter region^19^. The remaining 11 regions were only present in proximal tubule cells of the human kidney (Fig. 2a). Mouse kidney single-cell accessible chromatin analysis showed important conservation of the locus (Fig. S2a), including the genomic organization, the open chromatin at the promoter region, and the proximal tubule-specific open chromatin pattern. Human kidney bulk H3K27ac, H3K4me1, and H3K4me3 chromatin immunopre-cipitation (ChIP-seq) information further supported the single-cell data (Fig. 2a). By direct overlapping the kidney disease-associated SNPs with peaks observed in the snATAC-seq data, we found that the 5th, 6th, 8th, 9th, 10th, and 12th peaks harbored eGFR GWAS significant SNPs.

To narrow down the set of variants within peaks of accessible chromatin, we performed conditional analysis for eGFR GWAS to explain the observed pattern of association. We found that SNPs within peaks without GWAS SNPs (peaks 1, 3, 4, and 11) did not attenuate the association signal. Peaks 2 and 7 had no available SNPs. Adjustment for SNPs within peaks 5 and 12 did not completely account for the GWAS signal, with modest residual associations (*p* < 0.001) in the remaining regions. However, conditional analysis for SNPs in peaks 6, 8, 9, and 10 all demonstrated local attenuation of association (*P* > 0.001) (Fig. S2b).

Next, we performed CRISPR-based genome editing in cultured kidney cells (HEK293) to define the role of these regions in regulating *DPEP1* and *CHMP1A* expression (Fig. 2b). We opted to delete the entire prioritized region that contained the likely causal variant rather than performing individual SNP editing, given most reports suggest that multiple SNPs play roles in gene regulation^20,21^. Successful deletion of the region was confirmed by Sanger sequencing (Fig. S3a). *DPEP1* expression was increased following the deletion of peaks 8 and 9, whereas the *CHMP1A* expression was decreased when the 8th and 12th peaks were deleted (Fig. 2c). Interestingly, the cicero-based co-accessible analysis of the snATAC-seq in mouse kidneys further confirmed the shared regulation of these peaks (Fig. S2a). TFs binding site profiling from the ENCODE database^22^ indicated potential binding of proximal tubule-specific TFs, such as HNF1A, HNF4A, and HNF4G^23,24^ (Fig. S3b) and YY1, which mediates long-distance DNA interactions^25^ (Fig. S3b).

Taken together, integration of kidney function GWAS and snATAC-seq followed by CRISPR/Cas9 genome editing prioritized causal regulatory regions influencing the expression of both *DPEP1* and *CHMP1A* in human kidney proximal tubule cells.

### *Dpep1* deficiency ameliorates toxic renal injury induced by cisplatin or folic acid

**Fig. 3:**
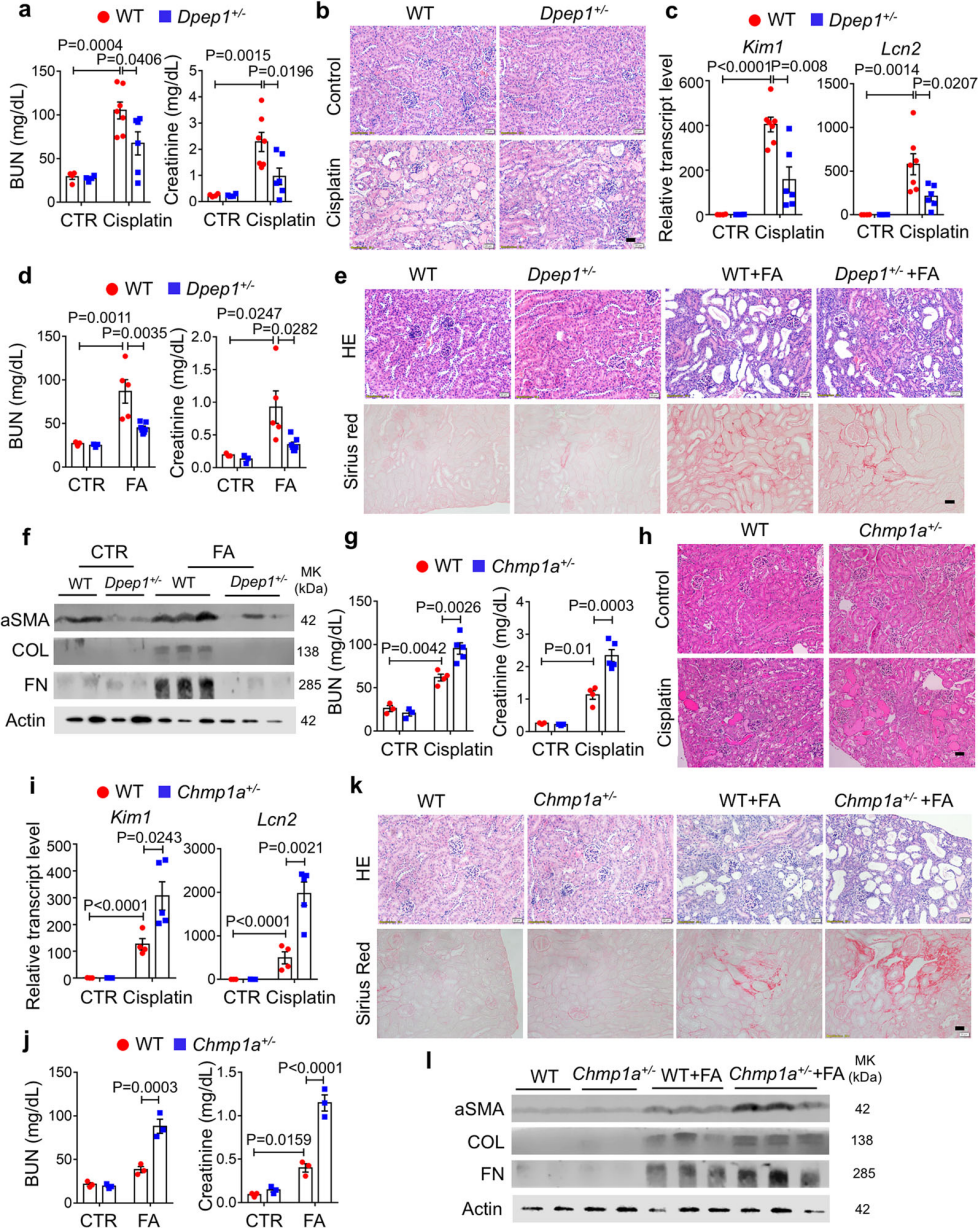
*Dpep1* deficiency ameliorated but *Chmp1a* haploinsufficiency exacerbated renal injury in mice. **a** Serum blood urea nitrogen (BUN) and creatinine measurement of control and *Dpep1*^*+/−*^ mice following sham or cisplatin injection. Sham-treated group: WT (*n* = 4), *Dpep1*^*+/−*^ (*n* = 4); cisplatin-treated group: WT (*n* = 7), *Dpep1*^*+/−*^ (*n* = 6). **b** Representative images of HE-stained kidney sections from control and *Dpep1*^*+/−*^ mice following sham or cisplatin injection. Scale bar: 20 μm. **c** Relative mRNA level of injury markers *Kim1* and *Lcn2* in the kidneys of control and *Dpep1*^*+/−*^ mice following sham or cisplatin injection. Sham-treated group: WT (*n* = 4), *Dpep1*^*+/−*^ (*n* = 4); cisplatin-treated group: WT (*n* = 7), *Dpep1*^*+/−*^ (*n* = 6). **d** Serum BUN and creatinine measurement of control and *Dpep1*^*+/−*^ mice following sham or folic acid (FA) injection. Sham-treated group: WT (*n* = 3), *Dpep1*^*+/−*^ (*n* = 3); cisplatin-treated group: WT (*n* = 5), *Dpep1*^*+/−*^ (*n* = 7). **e** Representative images of HE- and Sirius Red-stained kidney sections from control and *Dpep1*^*+/−*^ mice following sham or FA injection. Scale bar: 20 μm. **f** Western blots of fibrosis markers aSMA, Collagen3, and Fibronectin in kidneys of control and *Dpep1*^*+/−*^ mice following sham or FA injection. **g** Serum BUN and creatinine measurement of control and *Chmp1a*^*+/−*^ mice following sham or cisplatin injection. Sham-treated group: WT (*n* = 3), *Dpep1*^*+/−*^ (*n* = 3); cisplatin-treated group: WT (*n* = 4), *Dpep1*^*+/−*^ (*n* = 5). **h** Representative images of HE-stained kidney sections from control and *Chmp1a*^*+/−*^ mice following sham or cisplatin injection. Scale bar: 20 μm. **i** Relative transcript level of injury markers *Kim1* and *Lcn2* in control and *Chmp1a*^*+/−*^ mice following sham or cisplatin injection. Sham-treated group: WT (*n* = 3), *Dpep1*^*+/−*^ (*n* = 3); cisplatin-treated group: WT (*n* = 4), *Dpep1*^*+/−*^ (*n* = 5). **j** Serum BUN and creatinine levels of control and *Chmp1a*^*+/−*^ mice following sham or FA injection (*n* = 3 per group). **k** Representative images of HE- and Sirius Red-stained kidney sections from control and *Chmp1a*^*+/−*^ mice following sham or FA injection. Scale bar: 20 μm. **l** Western blots of fibrosis markers aSMA, Collagen3, and Fibronectin in control and *Chmp1a*^*+/−*^ mice following sham or FA injection. All data are represented as mean ± SEM. *P* value was calculated by two-way ANOVA with post hoc Tukey test. *P* < 0.05 is statistically significant. A Source Data file is available for this figure.

To understand the role of *Dpep1* in kidney disease development, we generated mice with genetic deletion of *Dpep1* using CRISPR/Cas9 technology. Exon 3 of *Dpep1* was targeted by two sgRNAs (Fig. S4a). Six out of thirty-five founders showed successful deletion by Sanger sequencing (Fig. S4b). Founder #5 containing a 62 base pair deletion at exon 3 was used in our experiment (Fig. S4c). Transcript and protein expression were decreased in *Dpep1*^*+/−*^ and *Dpep1*^*−/−*^ kidney tissue samples compared with control littermates (Fig. S4, e), indicating the successful generation of knockout mice. *Dpep1*^*+/−*^ and *Dpep1*^*−/−*^ mice were born at the expected Mendelian ratio and appeared normal. Gross phenotypic analysis of the *Dpep1* mutant mice showed no obvious changes in blood urea nitrogen (BUN) level, serum creatinine level, and kidney histology (Fig. 3a, b).

To understand the role of *Dpep1* in kidney disease, wild-type, *Dpep1*^*+/−*^, and *Dpep1*^*−/−*^ littermates were challenged with cisplatin, a chemotherapeutic with known proximal tubule toxicity^26^. Following cisplatin administration, *Dpep1*^*+/−*^ and *Dpep1*^*−/−*^ mice showed lower serum BUN and creatinine levels when compared to wild-type animals (Fig. 3a and Fig. S5a). Kidney histology, such as tubular dilation and immune cell infiltration was markedly lower in cisplatin-treated *Dpep1*^*+/−*^ and *Dpep1*^*−/−*^ mice when compared to wild-type mice (Fig. 3b, Figs. S4f and  S5b). Expression levels of acute kidney injury markers *Kim1* and *Lcn2*, and cytokines *Ccl2* and *Cd68*, were lower in the cisplatin-injected *Dpep1*^*+/−*^ mice when compared to wild-type littermates (Fig. 3c, Figs. S4g and  S5c).

We further investigated the role of *Dpep1* in the folic acid (FA) model, which is a mixed model of acute kidney injury leading to fibrosis development^27^. Serum BUN and creatinine levels in *Dpep1*^*+/−*^ mice with cisplatin treatment were lower than control mice (Fig. 3d). Structural changes analyzed on HE-stained kidney sections indicated attenuated tubule injury in *Dpep1*^*+/−*^ kidneys following FA injection (Fig. 3e) and less collagen on Sirius red-stained sections (Fig. 3e and Fig. S4h). Consistent with the histological changes, transcript and protein levels of profibrotic genes, including *Col1a1*, *Col3al*, *Fibronectin*, and *Vimentin*, were markedly decreased in *Dpep1*^*+/−*^ mice when compared to wild-type littermates after FA injection (Fig. 3f and Fig. S4i). *Dpep1* mutant mice also showed lesser kidney damage when compared to controls in the unilateral ureteral obstruction model (Fig. S4j). In summary, in vivo studies indicated that deletion of *Dpep1* ameliorated kidney injury in mice.

### *Chmp1a* haploinsufficiency exacerbates cisplatin and folic acid-induced kidney injury

To determine whether the second GWAS prioritized gene, *CHMP1A*, also plays a role in kidney disease development, we next imported *Chmp1a* mutant mice. *Chmp1a* null mice die at or soon after birth, whereas *Chmp1a* heterozygous mice are viable^28^. Kidneys of heterozygous mice showed lower expression of *Chmp1a* (Fig. S6a, b). By analyzing serum creatinine, BUN levels, and histological lesions on HE-stained kidney sections, no obvious kidney function and structural defect were observed in kidneys of *Chmp1a*^*+/−*^ mice at baseline (Fig. 3g, h).

To understand the role of *Chmp1a* in kidney injury, we analyzed wild-type and *Chmp1a*^*+/−*^ mice following cisplatin injection. Here we used a lower (20 mg/kg) cisplatin dose, as *Chmp1a*^*+/−*^ mice showed increased susceptibility to injury. Serum BUN and creatinine levels were higher in cisplatin-injected *Chmp1a*^*+/−*^ mice when compared to controls (Fig. 3g). Following cisplatin injection, *Chmp1a*^*+/−*^ mice displayed more severe histological changes, such as loss of proximal tubule brush border, tubular dilation, cast formation, and necrosis when compared to wild-type animals (Fig. 3h and Fig. S6c). Expression levels of injury markers, such as *Kim1* and *Lcn2*, and immune cell markers *Cd68* and *Ccl2*, were higher in the cisplatin-injected *Chmp1a*^*+/−*^ mice when compared to wild-type littermates (Fig. 3i and Fig. S6d). We also analyzed *Chmp1a*^*+/−*^ mice in the FA-induced kidney injury model. *Chmp1a*^*+/−*^ mice, when compared with wild-type animals, showed higher serum BUN and creatinine levels (Fig. 3j) and more severe tubule dilation when analyzed on H&E stained kidney section (Fig. 3k). We observed increased collagen accumulation by Sirius red-staining (Fig. 3k and Fig. S6e). Expression of fibrosis-associated genes, such as *Col1a1*, *Col3a1*, *Fibronectin*, and *Vimentin* were higher in the FA-injected *Chmp1a*^*+/−*^ mice when compared to wild-type littermates (Fig. 3l and Fig. S6f). When *Chmp1a*^*+/−*^ littermates were subjected to unilateral ureteral obstruction, *Chmp1a*^*+/−*^ mice also showed markedly increased injury compared to controls. Transcript levels of fibrosis markers *Col1a1*, *Fibronectin*, and *Vimentin*, were increased in *Chmp1a*^*+/−*^ mice compared to littermates (Fig. S6g). In summary, these data indicate the protective role of *Chmp1a* in kidney disease.

### *Dpep1* knockdown protects from cisplatin-induced injury without affecting necroptosis and pyroptosis

We next analyzed *Dpep1* expression in mouse kidneys and cultured kidney cells. Immunofluorescence staining indicated colocalization of DPEP1 with the proximal tubule marker Lotus Tetragonolobus Lectin (LTL), but not with collecting duct marker AQP2 or distal convoluted tubule marker Dolichos Biflorus Agglutinin (DBA) (Fig. S7a). The immunostaining results were consistent with the single-cell RNA^29^ and ATAC sequencing results (Fig. S7b and Fig. 2). Next, we analyzed the subcellular expression of DPEP1 in cultured kidney tubule cells by double staining with organelle markers. We did not observe colocalization with endosome markers RAB5, RAB7 and RAB11, and Golgi marker GM130. DPEP1 expression overlapped with Clathrin, a protein that plays a major role in the formation of coated vesicles near the plasma membrane in kidney proximal tubules (Fig. S7c).

**Fig. 4:**
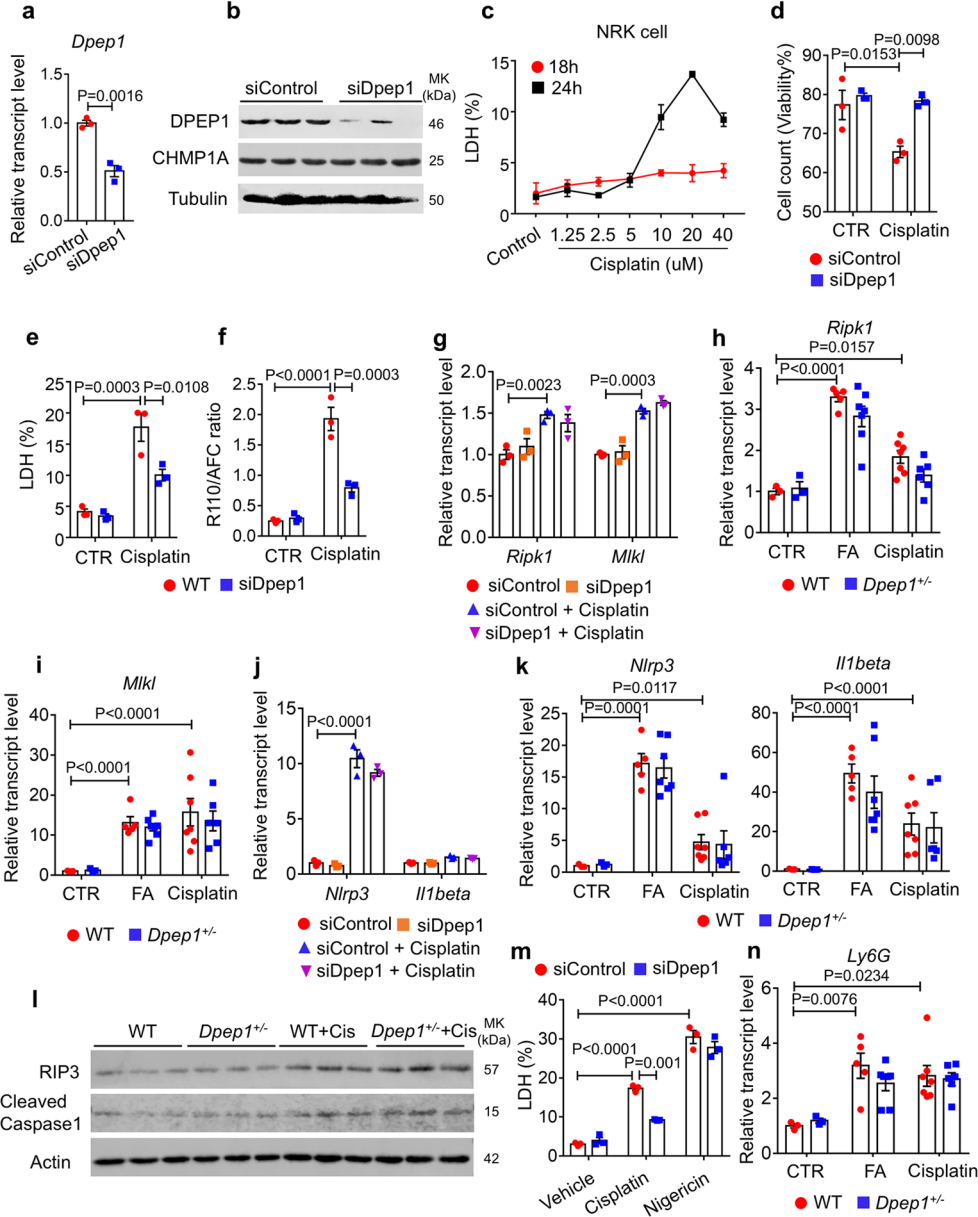
*Dpep1* deficiency protects from cisplatin-induced injury without affecting necroptosis or pyroptosis. **a** Relative mRNA level of *Dpep1* in scramble siRNA (siControl) and Dpep1 siRNA (siDpep1) transfected tubule cell (*n* = 3). **b** Western blots of DPEP1 and CHMP1A in scramble and *Dpep1* siRNA transfected tubule cell. **c** LDH level of NRK52E cell treated with varying dose of cisplatin for varying degree of time (*n* = 3). **d** The percentage of viable cells following siControl and siDpep1 transfection and in the presence and absence of cisplatin treatment (*n* = 3). **e** LDH level of following siControl and siDpep1 transfection and sham or cisplatin treatment (*n* = 3). **f** The ratio of cell-impermeable peptide substrate bis-AAF-R110 (dead cell indicator) to cell-permeable GF-AFC substrate (live cell indicator) from siControl and siDpep1 transfected cell following sham or cisplatin treatment (*n* = 3). **g** Relative transcript level of *Ripk1* and *Mlkl* of siControl and siDpep1 transfected cell following sham or cisplatin treatment (*n* = 3). **h** Relative transcript level of *Ripk1* in kidneys of folic acid and cisplatin-treated wild-type and *Dpep1*^*+/−*^ mice. Sham-treated group: WT (*n* = 3), *Dpep1*^*+/−*^ (*n* = 3); FA-treated group: WT (*n* = 5), *Dpep1*^*+/−*^ (*n* = 7); cisplatin-treated group: WT (*n* = 7), *Dpep1*^*+/−*^ (*n* = 6). **i** Relative transcript level of *Mlkl* in kidneys of folic acid and cisplatin-treated wild-type and *Dpep1*^*+/−*^ mice. Sham-treated group: WT (*n* = 3), *Dpep1*^*+/−*^ (*n* = 3); FA-treated group: WT (*n* = 5), *Dpep1*^*+/−*^ (*n* = 7); cisplatin-treated group: WT (*n* = 7), *Dpep1*^*+/−*^ (*n* = 6). **j** Relative transcript level of *Nlrp3* and *Il1beta* from siControl and siDpep1 transfected cell following sham or cisplatin treatment (*n* = 3). **k** Relative transcript level of *Nlrp3* and *Il1beta* in kidneys of folic acid and cisplatin-treated wild-type and *Dpep1*^*+/−*^ mice. Sham-treated group: WT (*n* = 3), *Dpep1*^*+/−*^ (*n* = 3); FA-treated group: WT (*n* = 5), *Dpep1*^*+/−*^ (*n* = 7); cisplatin-treated group: WT (*n* = 7), *Dpep1*^*+/−*^ (*n* = 6). **l** Western blots of RIPK3 and cleaved caspase 1 in kidneys of wild-type and *Dpep1*^*+/−*^ mice following sham or cisplatin treatment. **m** LDH level of siControl and siDpep1 transfected cell following sham or Nigericin treatment (*n* = 3). **n** Relative mRNA level of *Ly6G* in kidneys of control, folic acid and cisplatin-treated wild-type and *Dpep1*^*+/−*^ mice. Sham-treated group: WT (*n* = 3), *Dpep1*^*+/−*^ (*n* = 3); FA-treated group: WT (*n* = 5), *Dpep1*^*+/−*^ (*n* = 7); cisplatin-treated group: WT (*n* = 7), *Dpep1*^*+/−*^ (*n* = 6). All data are represented as mean ± SEM. *P* value was calculated by two-way ANOVA with post hoc Tukey test for **c**–**n**. *P* value was calculated by two-tailed *t*-test for **a**. *P* < 0.05 is statistically significant. A Source Data file is available for this figure.

To define the mechanism of *Dpep1*-mediated kidney injury, we transfected rat renal epithelial cells with siRNA targeted to *Dpep1* (siDpep1) or scramble siRNA (siControl). *Dpep1* expression was decreased, while *Chmp1a* level was not affected, in siDpep1 transfected cells (Fig. 4a, b). To mimic tubule epithelial cell (TEC) injury observed in mice, TECs were treated with cisplatin. We observed the highest cytotoxicity with 20 µM cisplatin dose after 24 h of treatment (Fig. 4c). Cell count by trypan blue stain showed improved cell viability in siDpep1 transfected cells compared to siControl cells after cisplatin treatment (Fig. 4d). Cytotoxicity measured by LDH release confirmed the improved cell viability of siDpep1 transfected cells (Fig. 4e). Cytotoxicity measured as a ratio of dead cell substrate bis-AAF-R110 and live cell substrate GF-AFC showed a lower R110/AFC ratio in siDpep1 transfected cells after cisplatin treatment (Fig. 4f), again confirming the protective role of *Dpep1* knockdown from cisplatin-induced cytotoxicity.

Next, we set out to understand pathways that mediate the *Dpep1* knockdown-afforded protection from TEC cytotoxicity. Transcript levels of *Ripk1* and *Mlkl*, genes involved in the necroptosis, were increased following cisplatin treatment; however, they were not affected in siDpep1 cells (Fig. 4g). Similarly, expression of *Ripk1*, *Ripk3*, and *Mlkl* was increased in injured mouse kidney samples, but there was no difference between diseased wild-type and *Dpep1*^*+/−*^ kidneys (Fig. 4h, i, l). Pyroptosis markers, such as *Nlrp3, Il1beta*, and cleaved caspase 1 were elevated in cisplatin-treated cells and kidneys of cisplatin- or FA-treated mice. We did not observe differences when cisplatin-treated control cells were compared to *Dpep1* knockdown cells or *Dpep1* knockout kidneys (Fig. 4j–l). We also observed no difference in LDH release following nigericin-induced *Nlrp3* activation when control and *Dpep1* knockdown cells were compared (Fig. 4m). Recently, *Dpep1* was shown to play a role in cell adhesion for neutrophil recruitment in the lung and liver^30^; however, we found no difference in expression of the neutrophil marker *Ly6G* in kidneys of control and diseased *Dpep1*^*+/−*^ mice (Fig. 4n). In summary, our data failed to support the role of pyroptosis and necroptosis in *Dpep1* knockdown-mediated protection from cisplatin-induced cytotoxicity.

### *Chmp1a* knockdown sensitizes cisplatin-induced cell death without altering necroptosis, pyroptosis, and apoptosis

We first examined the expression of *Chmp1a* in mouse kidney tissue and cell lines. Immunofluorescence staining showed that CHMP1A colocalized with the proximal tubule marker LTL, loop of Henle marker Peanut Agglutinin (PNA), collecting duct marker AQP2, and distal convoluted tubule marker DBA, indicating that CHMP1A was expressed in all tubular segments (Fig. S8a). CHMP1A is a component of endosomal sorting complexes required for transport (ESCRT). We found that CHMP1A was not colocalized with early endosome markers EEA1 and RAB5, and recycling endosome marker RAB11, but colocalized with late endosome markers RAB7 and VAMP7 (Fig. S8b).

**Fig. 5:**
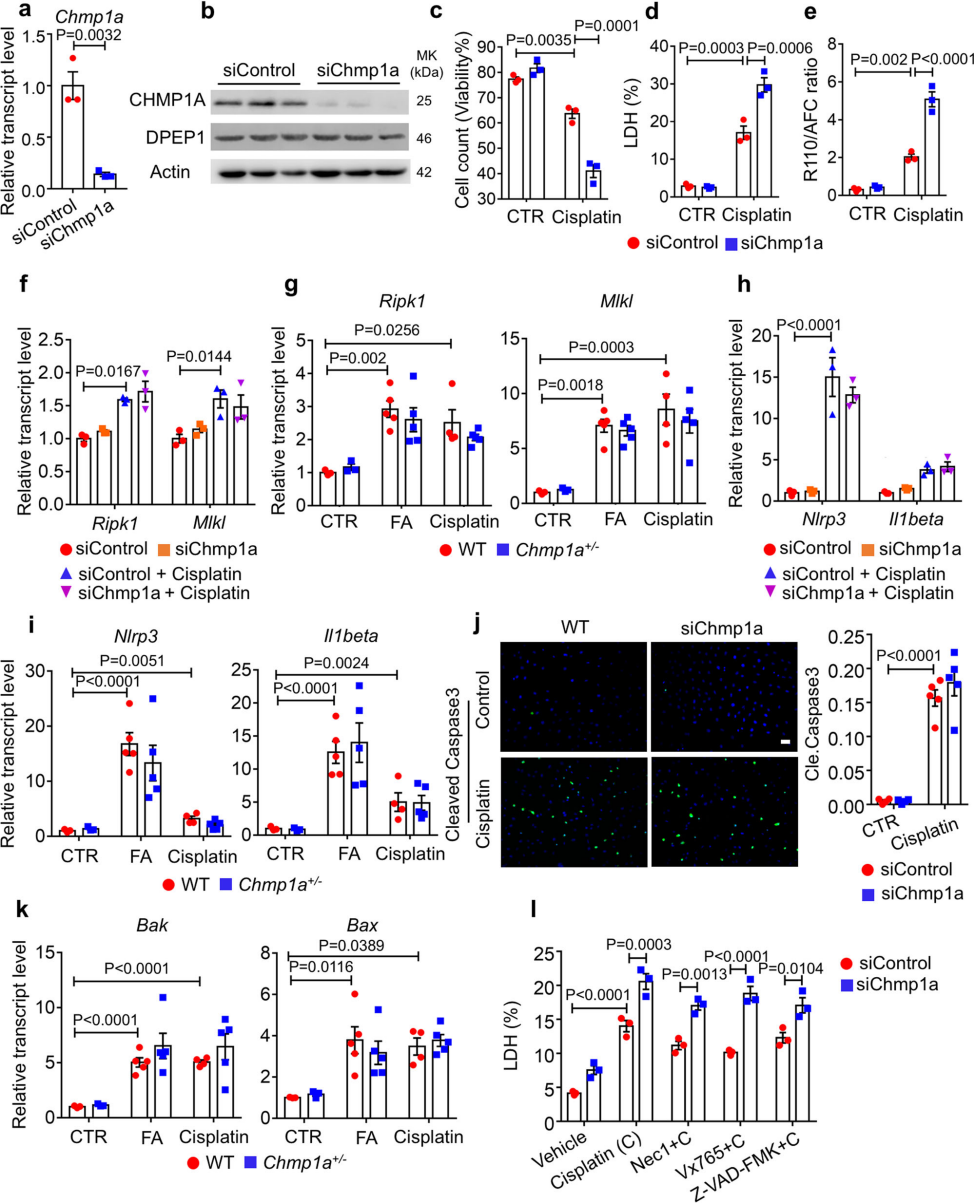
*Chmp1a* sensitizes for cisplatin-induced cell death without altering necroptosis, pyroptosis, and apoptosis. **a** Relative mRNA level of *Chmp1a* in scramble siRNA (siControl) and *Chmp1a* siRNA (siChmp1a) transfected kidney tubule cell (*n* = 3). **b** Western blots of CHMP1A and DPEP1 from siControl and siChmp1a transfected cells. **c** Viability of siControl and siChmp1a transfected tubule cell following sham or cisplatin treatment (*n* = 3). **d** LDH level of siControl and siChmp1 transfected tubule cell following sham or cisplatin treatment (*n* = 3). **e** The ratio of cell-impermeable peptide substrate bis-AAF-R110 (dead cell indicator) to cell-permeable GF-AFC substrate (live cell indicator) from siControl siChmp1a transfected cell following sham or cisplatin treatment (*n* = 3). **f** Relative mRNA level of *Ripk1* and *Mlkl* of siControl and siChmp1a transfected cell following sham or cisplatin treatment (*n* = 3). **g** Relative mRNA level of *Ripk1* and *Mlkl* in kidneys of folic acid and cisplatin-treated wild-type and *Chmp1a*^*+/−*^ mice. Sham-treated group: WT (*n* = 3), *Chmp1a*^*+/−*^ (*n* = 3); FA-treated group: WT (*n* = 5), *Chmp1a*^*+/−*^ (*n* = 5); cisplatin-treated group: WT (*n* = 4), *Chmp1a*^*+/−*^ (*n* = 5). **h** Relative mRNA level of *Nlrp3* and *Il1beta* of siControl and siChmp1a transfected cell following sham or cisplatin treatment (*n* = 3). **i** Relative mRNA level of *Nlrp3* and *Il1beta* in kidneys of folic acid and cisplatin-treated wild-type and *Chmp1a*^*+/−*^ mice. Sham-treated group: WT (*n* = 3), *Chmp1a*^*+/−*^ (*n* = 3); FA-treated group: WT (*n* = 5), *Chmp1a*^*+/−*^ (*n* = 5); cisplatin-treated group: WT (*n* = 4), *Chmp1a*^*+/−*^ (*n* = 5). **j** (left) Representative cleaved Caspase 3 staining of siControl and siChmp1a transfected cell following sham or cisplatin treatment. (right) Quantification of cleaved Caspase 3 positive cell (*n* = 5). Scale bar: 20 μm. **k** Relative mRNA transcript of *Bak* and *Bax* in kidneys of folic acid and cisplatin-treated wild-type and *Chmp1a*^*+/−*^ mice. Sham-treated group: WT (*n* = 3), *Chmp1a*^*+/−*^ (*n* = 3); FA-treated group: WT (*n* = 5), *Chmp1a*^*+/−*^ (*n* = 5); cisplatin-treated group: WT (*n* = 4), *Chmp1a*^*+/−*^ (*n* = 5). **l** LDH level of siControl and siChmp1a transfected cell with or without cisplatin and necroptosis (Nec1), pyroptosis (Vx765), and apoptosis (Z-VAD-FMK) inhibitors (*n* = 3). All data are represented as mean ± SEM. *P* value was calculated by two-way ANOVA with post hoc Tukey test for **c**–**l**. *P* value was calculated by two-tailed t-test for **a**. *P* < 0.05 is statistically significant. A Source Data file is available for this figure.

Consistent with the in vivo data, we found that rat TEC cells NRK52E expressed high levels of *Chmp1a*. We used *Chmp1a* siRNA, which reduced *Chmp1a* but not *Dpep1* levels (Fig. 5a, b). We found that cell viability was lower in siChmp1a treated cells when compared to controls, following cisplatin treatment (Fig. 5c). Both LDH release and cell death indicator, R110/AFC, were significantly increased in the siChmp1a cells after cisplatin treatment (Fig. 5d, e). These results indicated increased cytotoxicity in *Chmp1a* deficient kidney tubule cells.

We found that markers of necroptosis and pyroptosis, such as *Ripk1*, *Mlkl*, *Nlrp3*, and *Il1beta* were increased in cisplatin-treated cells and mice; however, we observed comparable change in their expression levels in siChmp1a treated cells and *Chmp1a*^*+/−*^ kidneys (Fig. 5f–i). Looking at genes associated with apoptosis, we found no obvious difference of cleaved caspase 3-positive cells when siControl and siChmp1a cells were compared after cisplatin treatment (Fig. 5j). Transcript levels of *Bak* and *Bax* were comparable in injured control and *Chmp1a*^*+/−*^ kidneys (Fig. 5k). To confirm the role of *Chmp1a* in necroptosis, pyroptosis and apoptosis, siControl and siChmp1a cells were pretreated with the necroptosis inhibitor necrostatin-1 (Nec-1), the pyroptosis inhibitor Vx765 and a pan-caspase inhibitor Z-VAD-FMK followed by cisplatin treatment. We observed no change in LDH release following co-incubation with any of these inhibitors (Fig. 5l). In summary, *Chmp1a* deficiency increased cisplatin-induced cell death with no observable change in classical genes involved in necroptosis, pyroptosis, and apoptosis.

### *Dpep1* knockdown ameliorates cisplatin-induced apoptosis and ferroptosis

**Fig. 6:**
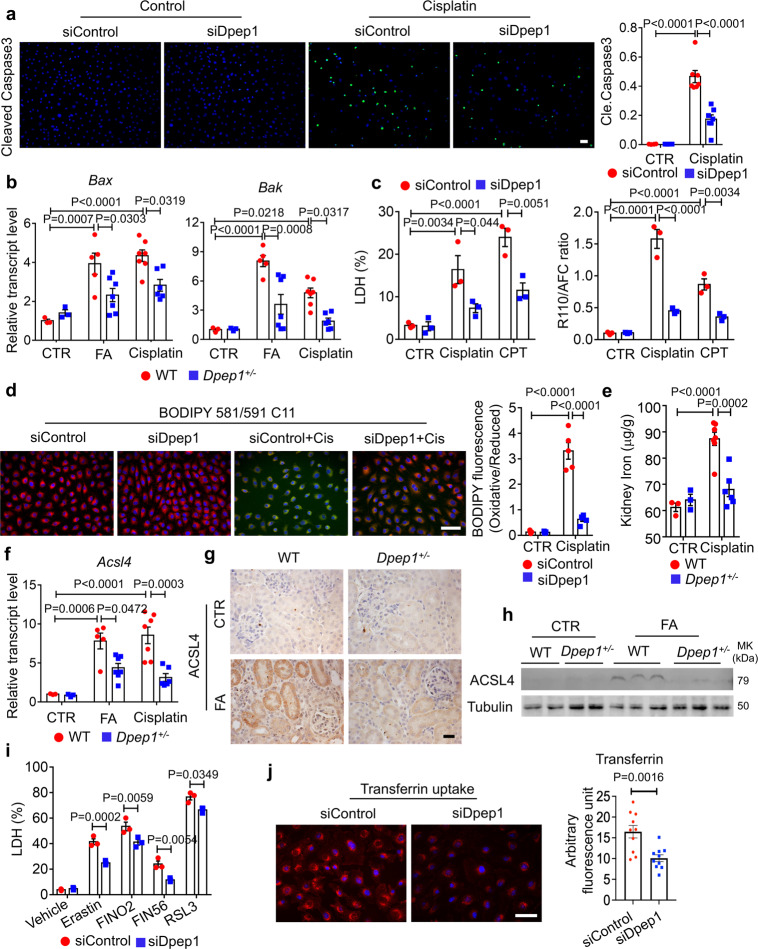
*Dpep1* knockdown ameliorated cisplatin-induced apoptosis and ferroptosis. **a** (Left) Representative cleaved caspase 3 staining of siControl and siDpep1 cell following sham or cisplatin treatment. (Right) Quantification of positive cleaved caspase 3 cell (*n* = 7). Scale bar: 20 μm. **b** Relative mRNA transcript of *Bax* and *Bak* in kidneys of folic acid and cisplatin-treated wild-type and *Dpep1*^*+/−*^ mice. Sham-treated g group: WT (*n* = 3), *Dpep1*^*+/−*^ (*n* = 3); FA-treated group: WT (*n* = 5), *Dpep1*^*+/−*^ (*n* = 7); cisplatin-treated group: WT (*n* = 7), *Dpep1*^*+/−*^ (*n* = 6). **c** (Left) LDH level of siControl and siDpep1 transfected cell with or without apoptosis activator camptothecin (CPT) treatment. (Right) The ratio of cell-impermeable peptide substrate bis-AAF-R110 (dead cell indicator) to cell-permeable GF-AFC substrate (live cell indicator) of siControl and siDpep1 transfected cell with or without CPT treatment (*n* = 3). **d** (left) Representative BODIPY 581/591 C11 fluorescence of siControl and siDpep1 transfected cell following sham or cisplatin treatment. (right) Quantification of the oxidized vs. reduced probe (*n* = 5). Scale bar: 50 μm. **e** Ferrous iron concentration (normalized to kidney weight) in kidneys of cisplatin-treated wild-type and *Dpep1*^*+/−*^ mice. Sham-treated group: WT (*n* = 3), *Dpep1*^*+/−*^ (*n* = 3); cisplatin-treated group: WT (*n* = 7), *Dpep1*^*+/−*^ (*n* = 6). **f** Relative mRNA level of *Acsl4* in kidneys of folic acid and cisplatin-treated wild-type and *Dpep1*^*+/−*^ mice. Sham-treated group: WT (*n* = 3), *Dpep1*^*+/*^^−^ (*n* = 3); FA-treated group: WT (*n* = 5), *Dpep1*^*+/−*^ (*n* = 7); cisplatin-treated group: WT (*n* = 7), *Dpep1*^*+/−*^ (*n* = 6). **g** Representative images of ACSL4 immunostaining in kidney sections of folic acid-treated wild-type and *Dpep1*^*+/−*^ mice. Scale bar: 10 μm. **h** Western blots of ACSL4 kidneys of folic acid-treated wild-type and *Dpep1*^*+/−*^ mice. **i** LDH level of scramble siControl and siDpep1 transfected cell following sham or ferroptosis activator Erastin, FIN56, FINO2, and RSL3 treatment (*n* = 3). **j** (Left) Representative images of transferrin uptake of siControl and siDpep1 transfected cells. (Right) Quantification of arbitrary fluorescence unit of transferrin in siControl and siDpep1 transfected cell (*n* = 10). Scale bar: 50 μm. All data are represented as mean ± SEM. *P* value was calculated by two-way ANOVA with post hoc Tukey test for **a**–**i** and was calculated by two-tailed *t*-test for **j**. *P* < 0.05 is statistically significant. A Source Data file is available for this figure.

Next, we examined the role of *Dpep1* in cisplatin-induced programmed cell death. The number of cleaved caspase 3-positive cells was lower in siDpep1 transfected cells compared to siControl cells following cisplatin administration (Fig. 6a). Transcript expression of *Bax* and *Bak* was lower in *Dpep1*^*+/−*^ kidneys both in the FA and cisplatin models compared to controls (Fig. 6b). LDH release and the ratio of R110/AFC indicated increased cytotoxicity following treatment with the apoptosis-inducer camptothecin (CPT). We observed improved cell viability in *Dpep1* knockdown TEC when compared to control cells (Fig. 6c). TUNEL assay further confirmed the lower cell death rate in kidneys of cisplatin-treated *Dpep1*^*−/−*^ mice when compared with wild-type littermates (Fig. S5d). Taken together, these data indicated that *Dpep1*-mediated cell death was associated with caspase 3 cleavage.

We next examined the potential role of ferroptosis in *Dpep1*-loss offered protection from cell death. Toxic lipid ROS accumulation is a feature of ferroptosis, which can be monitored by C11 BODIPY 581/591. siControl-transfected cells treated with cisplatin exhibited a robust green signal indicating lipid oxidation, whereas siDpep1 transfection had lower lipid oxidation upon cisplatin treatment (Fig. 6d). Iron catalyzes ferroptosis, and we observed lower iron content in *Dpep1*^*+/−*^ kidney lysates after cisplatin administration than in control kidneys (Fig. 6e). Ferroptosis is associated with mitochondrial damage^31,32^. MitoSOX staining was increased upon cisplatin treatment; however, siDpep1 transfection attenuated the increase (Fig. S9a). In addition, we observed a marked increase of long-chain-fatty-acid-CoA ligase 4 (*Acsl4*) expression, an important marker of ferroptosis, in diseased wild-type kidneys but not in *Dpep1*^*+/*^^−^ kidneys (Fig. 6f–h). Similarly, the expression of *Acsl4* was increased in the cisplatin-treated siControl but not in siDpep1 cells (Fig. S9b). To further explore the role of *Dpep1* in ferroptosis, we analyzed the effect of the ferroptosis activators erastin, FINO2, FIN56, and RSL3. *Dpep1* knockdown cells had lower LDH release following erastin, FINO2, FIN56, and RSL3 when compared to siControl-transfected cells (Fig. 6i). BODIPY 581/591 probe labeling showed lower lipid peroxidation in cisplatin-treated primary tubular epithelial cells cultured from *Dpep1*^*+/−*^ mice (Fig. S9c). The primary tubular epithelial cells obtained from *Dpep1*^*+/−*^ mice also showed less LDH release following cisplatin, CPT, and erastin treatment (Fig. S9d). As *Dpep1* was reported to regulate radiographic contrast uptake in TEC^33^, we explored whether *Dpep1* regulated dextran and transferrin uptake. We observed both lower dextran (Fig. S9e) and transferrin uptake in siDpep1 TEC compared to scramble siRNA cell (Fig. 6j), suggesting that *Dpep1* deficiency results in decreased iron import, leading to lower intracellular iron concentration higher lipid peroxidation threshold and lower ferroptosis.

*Dpep1* has been proposed to regulate glutathione (GSH) levels, which is the main substrate of the key ferroptosis regulator *Gpx4*. As shown in Fig. S10a, the total and free GSH level was higher in kidneys of *Dpep1*^*−/−*^ mice compared to the wild-type mice with or without cisplatin treatment. Interestingly, cisplatin had no effect on the total and free GSH level and *Gpx4* mRNA level in the *Dpep1*^*−/−*^ mice (Fig. S10a, b). In addition, we observed no change in mRNA and protein level of GPX4 level in *Dpep1*^*−/−*^ mice compared with the wild-type mice (Fig. S10b, c). Protein level of GPX4 was slightly lower in the kidneys of the wild-type cisplatin-treated mice (Fig. S10c).

To further confirm the role of *Dpep1*, we transfected NRK-52E cells with DPEP1 plasmid. We observed markedly higher LDH release in DPEP1 transfected cells, which was further increased following cisplatin treatment (Fig. S11a). Lipid peroxidation by BODIPY 581/591 C11 was also higher in DPEP1 transfected cells (Fig. S11b), supporting the deleterious role of DPEP1. An important limitation of this experiment remains the supraphysiological level of DPEP1 in these cells.

### *Chmp1a* knockdown enhances ferroptosis through increased iron accumulation

**Fig. 7:**
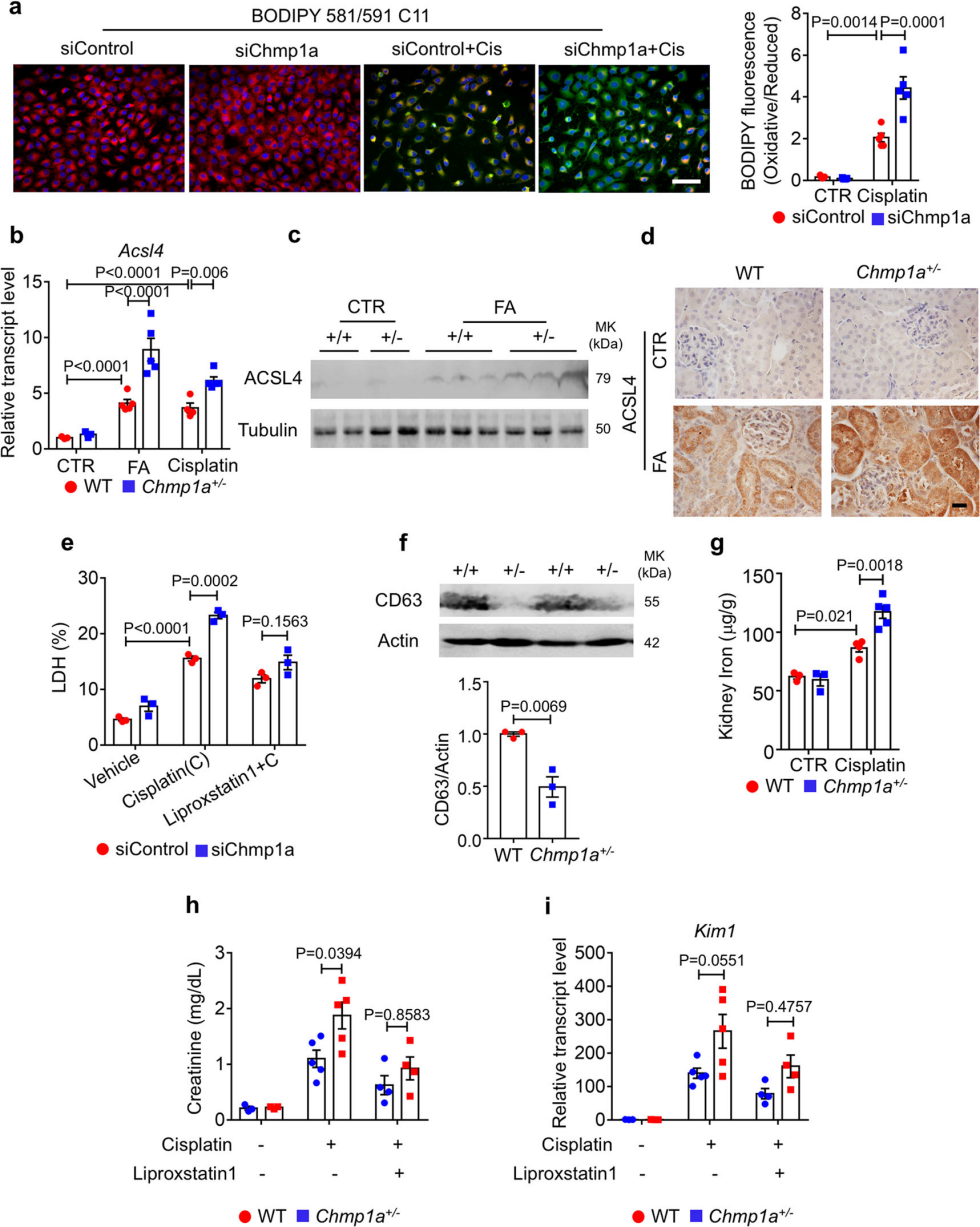
*Chmp1a* knockdown enhanced ferroptosis through iron accumulation. **a** (left) Representative BODIPY 581/591 C11 staining of siControl and siChmp1a transfected cell following sham or cisplatin treatment. Scale bar: 50 μm. (right) Quantification of the oxidized vs. reduced probe (*n* = 5). **b** Relative mRNA level of *Acsl4* in the kidneys of folic acid and cisplatin-treated wild-type and *Chmp1a*^*+/−*^ mice. Sham-treated group: WT (*n* = 3), *Chmp1a*^*+/−*^ (*n* = 3); FA-treated group: WT (*n* = 5), *Chmp1a*^*+/−*^ (*n* = 5); cisplatin-treated group: WT (*n* = 4), *Chmp1a*^*+/−*^ (*n* = 5). **c** Western blots of ACSL4 in kidneys of folic acid-treated wild-type and *Chmp1a*^*+/*^^−^ mice. **d** Representative images of ACSL4 immunostaining of kidney sections from folic acid-treated wild-type and *Chmp1a*^*+/−*^ mice. Scale bar: 10 μm. **e** LDH level of siControl and siChmp1a transfected cell with or without cisplatin and ferroptosis inhibitor liproxstatin1 (*n* = 3). **f** (upper) Western blots of CD63 of kidney exosomes of wild-type and *Chmp1a*^*+/−*^ mice. (bottom) Quantification of three independent experiments (*n* = 3). **g** Ferrous iron concentrations (normalized to kidney weight) of kidneys of cisplatin-treated wild-type and *Chmp1a*^*+/*^^−^ mice. Sham-treated group: WT (*n* = 3), *Chmp1a*^*+/−*^ (*n* = 3); cisplatin-treated group: WT (*n* = 4), *Chmp1a*^*+/−*^ (*n* = 5). **h** Serum creatinine level of control and *Chmp1a*^*+/−*^ mice with or without cisplatin and liproxstatin injection. Sham-treated group: WT (*n* = 3), *Chmp1a*^*+/−*^ (*n* = 3); cisplatin-treated group: WT (*n* = 5), *Chmp1a*^*+/*^^−^ (*n* = 5); cisplatin and liproxstatin-treated group: WT (*n* = 4), *Chmp1a*^*+/−*^ (*n* = 4). **i** Relative transcript level of injury marker *Kim1* in kidneys of control and *Chmp1a*^*+/*^^−^ mice with or without cisplatin and liproxstatin injection. Sham-treated group: WT (*n* = 3), *Chmp1a*^*+/*^^−^ (*n* = 3); cisplatin-treated group: WT (*n* = 5), *Chmp1a*^*+/*^^−^ (*n* = 5); cisplatin and liproxstatin-treated group: WT (*n* = 4), *Chmp1a*^*+/−*^ (*n* = 4). All data are represented as mean ± SEM. *P* value was calculated by two-way ANOVA with post hoc Tukey test for **a**, **b**, **e**, **g**, **h**, **i**. *P* value was calculated by two-tailed *t*-test for **f**. *P* < 0.05 is statistically significant. A Source Data file is available for this figure.

Next, we examined the potential role of *Chmp1a* in ferroptosis. *Chmp1a* knockdown (siChmp1a) cells, compared to siControl after cisplatin treatment, showed higher lipid peroxidation (oxidized BODIPY 581/591 C11 fluorescence) (Fig. 7a). The transcript level of *Acsl4* was higher in *Chmp1a*^*+/−*^ kidneys and siChmp1a cell after cisplatin or FA administration (Fig. 7b–d and Fig. S12a). Moreover, *Chmp1a* knockdown cells showed increased mitochondrial damage following injury by mitoSOX fluorescence (Fig. S12b). Administration of Mito-TEMPO, an inhibitor of mitochondrial ROS production, markedly lowered LDH release (Fig. S12c) and lipid peroxidation (Fig. S12d) induced by cisplatin in *Chmp1a*^*+/−*^ primary TECs. To further assess the potential role of *Chmp1a* in ferroptosis, siControl, and siChmp1a cells were pretreated with the ferroptosis inhibitor liproxstatin1 prior to cisplatin treatment. While cisplatin-induced LDH release was increased upon *Chmp1a* knockdown, liproxstatin reduced this effect (Fig. 7e), indicating that the cisplatin cytotoxicity effect on tubule cell is mediated by *Chmp1a*. Primary TECs obtained from wild-type or *Chmp1a*^*+/−*^ mice with liproxstatin1 treatment also showed lower lipid peroxidation (C11 BODIPY 581/591) as siRNA treated cells (Fig. S12d). Treatment of primary TEC with inhibitors of ferroptosis, necroptosis, apoptosis, and pyroptosis indicated that only the ferroptosis inhibitor liproxstatin1 alleviated the cisplatin-induced cytotoxicity in *Chmp1a*^*+/−*^ primary TECs (Fig. S12e).

Next, we wanted to understand the mechanism of *Chmp1a*-mediated ferroptosis protection. *Chmp1a* has been reported to be involved in intraluminal vesicle formation in multivesicular bodies (MVBs). *Chmp1a* null cells have been shown to shed fewer CD63 positive exosomes^28^. Formation of ferritin-containing MVBs/exosomes controls iron efflux and ferroptosis^34,35^, therefore, impairment of exosome formation in the *Chmp1a*^*+/−*^ kidneys might explain iron accumulation and increased sensitivity to ferroptosis. We quantified exosome formation by CD63 in *Chmp1a*^*+/−*^ kidneys (Fig. 7f). Consistent with the potential role of *Chmp1a* in exosome formation, iron concentration in cisplatin-injected *Chmp1a*^*+/−*^ kidney lysates was higher than cisplatin-injected control kidneys (Fig. 7g).

To confirm the role of ferroptosis in vivo in cisplatin-induced *Chmp1a*-mediated cell death, we injected wild-type and *Chmp1a*^*+/−*^ mice with liproxstatin and sham. Liproxstatin1 protected *Chmp1a*^*+/−*^ mice from cisplatin-induced injury and reduced serum creatinine level (Fig. 7h**)**. Kidney injury marker *Kim1* was lower in liproxstatin injected *Chmp1a*^*+/−*^ mice (Fig. 7i). In summary, these data suggested that *Chmp1a* plays a role in exosome formation and iron export.

### *DPEP1* and *CHMP1A* levels strongly correlated with each other and other ferroptosis genes in human kidney samples

**Fig. 8:**
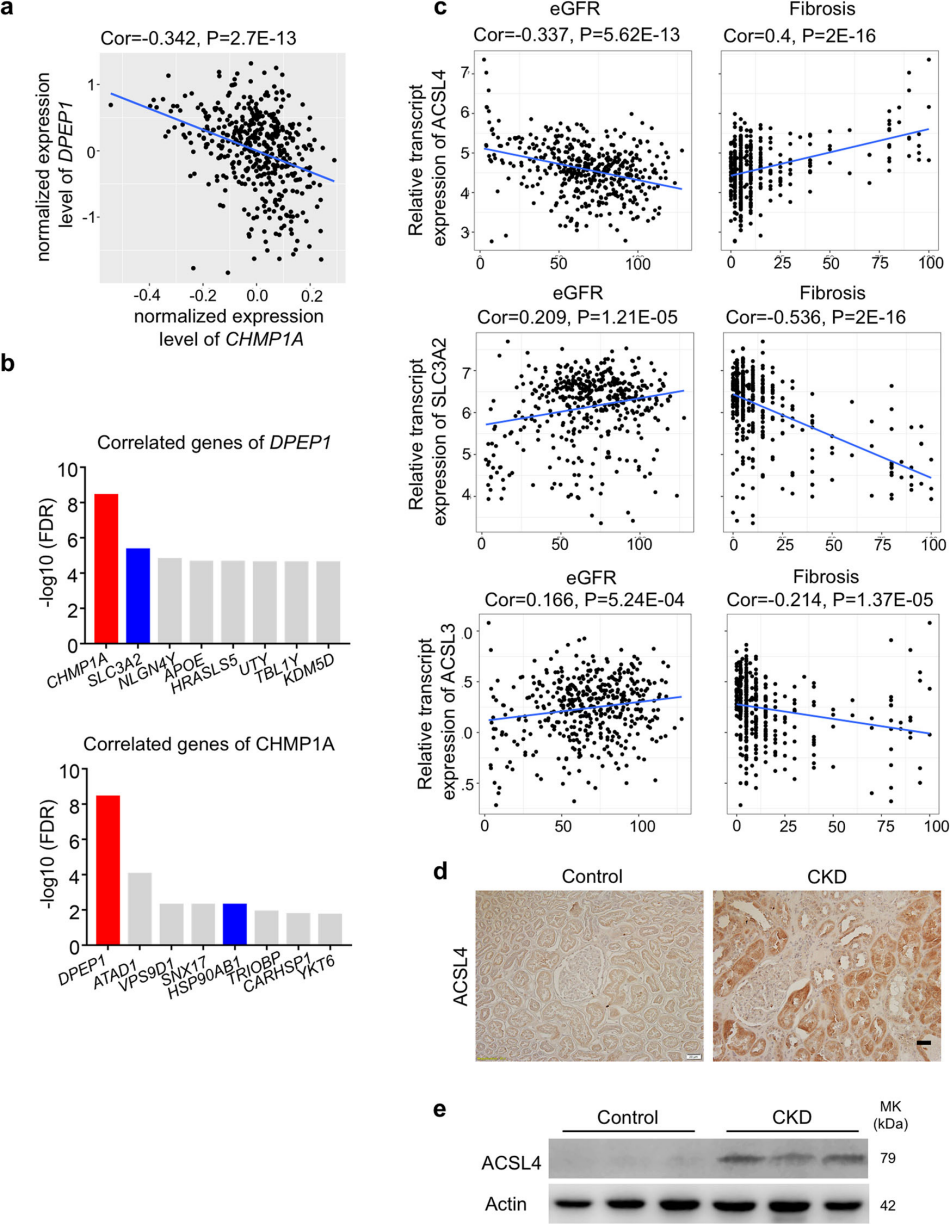
*DPEP1* and *CHMP1A* levels correlate and associated with ferroptosis gene expression in human kidney samples. **a** Relative expression of *DPEP1* (y-axis) and *CHMP1A* (x-axis) in 432 microdissected human kidney tubule samples. Pearson correlation is shown. Student *t*-test based on the Pearson correlation coefficient was used to calculate the statistical significance of the association. **b** Genes showing significant correlation with *DPEP1* and *CHMP1A* in microdissected human kidney tubule samples. y*-*axis represents the Pearson correlation *P* value. **c** Relative transcript levels of *ACSL4*, *ACSL3*, and *SLC3A2* (FPKM, y-axis), and kidney function (eGFR, x-axis) or kidney fibrosis (x-axis) as analyzed in 432 microdissected human kidney samples. Pearson correlation is shown. Student’s *t*-test based on the Pearson correlation coefficient was used to calculate the statistical significance of the association. **d** Representative images of ACSL4 immunostaining in healthy control and CKD kidney samples. Scale bar: 20 μm. **e** Western blots of ACSL4 in healthy control and CKD kidney samples.

Finally, we wanted to understand if we could recapitulate changes observed in *Dpep1* and *Chmp1a* mice in human kidney samples. First, we examined RNA sequencing data from 432 microdissected human kidney samples. The collection included subjects with normal kidney function and absence of fibrosis and also samples with diabetic or hypertensive kidney disease. The clinical information is shown in Supplementary Data 3. We first identified genes whose expression was strongly correlated with *DPEP1*. In keeping with our results, indicating a common regulatory region for both genes, we found that the top correlating gene for *DPEP1* was *CHMP1A* (*P* = 2.7E−13) (Fig. 8a). *Dpep1* and *Chmp1a* levels also strongly and negatively correlated in mouse models of kidney disease induced by UUO, FA, APOL1, and PGC1a (Fig. S13a). The correlations of *DPEP1*/*CHMP1A* expression with eGFR and fibrosis of bulk human kidney tissue were less consistent, albeit cell fraction changes could have influenced this (Fig. S13b).

Next, we analyzed the list of genes that showed strong correlation with *DPEP1* or *CHMP1A* expression (Fig. 8b). One of the top genes that correlated with *DPEP1* level was *SLC3A2* (Fig. 8b), a neutral amino acid transporter important for cysteine transport with a key role in ferroptosis^36^. Amongst the top genes that correlated with *CHMP1A* level was *HSP90* (Fig. 8b), a chaperone that was shown to regulate ferroptosis^37^. Gene ontology analysis for genes that correlated with either *CHMP1A* or *DPEP1*, respectively, showed enrichment for metabolic processes (Fig. S14 and Supplementary Data 4).

We found that the expression of ferroptosis activator *ACSL4* showed a strong negative correlation with kidney function (eGFR) and positive correlation with fibrosis, and the expression of *ACSL3* and *SLC3A2* showed a positive correlation with eGFR and negative correlation with fibrosis (Fig. 8c), supporting an important role for ferroptosis in patients with CKD and fibrosis. We also found an increase in protein expression of ACSL4 in human kidney samples with CKD (Fig. 8d, e). In summary, our results indicate a strong correlation between the expression of *DPEP1* and *CHMP1A* in human kidney samples, supporting their shared regulation. In addition, their levels correlated with several key ferroptosis regulators.

## Discussion

Here we identified *DPEP1* and *CHMP1A* as kidney disease genes via the triangulation of genome-wide association studies, human kidney mQTL and eQTL data. We fine mapped the locus via single-cell chromatin accessibility annotation, conditional analysis of the GWAS and CRISPR/Cas9 mediated genome deletion studies to identify common regulatory regions for *DPEP1* and *CHMP1A* in proximal tubules. Analysis of *Dpep1* and *Chmp1a* haploinsufficient mice demonstrated the functional role of both *Dpep1* and *Chmp1a* in kidney injury. On the molecular level, we show that both genes are crucial regulators of ferroptosis. While *Dpep1* altered iron import, *Chmp1a* interfered with iron export, indicating an important mechanistic convergence and likely explaining the linked regulation of these genes.

Defining the key genes and regulatory mechanism for kidney disease is a critically important next step for therapeutic development. Kidney function heritability is estimated to be around 50%^38^. There are more than 250 loci showing genome-wide significant association with kidney function^3^. As of now, functional annotation of target genes has only been performed for a handful of loci, such as *UMOD*^39^, *DAB2*^40^*, SHROOM3*^41^*, DACH1*^42^, and *MANBA*^43^. These studies indicated the critical role of the endolysosomal system in proximal tubule cells in disease development.

While the GTEx compendium highlighted that a single variant might show association with the expression of multiple genes, this study is one of the first studies to demonstrate that multiple genes actually contribute to disease development at a single fine-mapped genetic locus. While the GWAS, methylation, and expression of *DPEP1* and *CHMP1A* share common causal variants using Bayesian molocalization analysis, transcriptome-wide association analysis and Mendelian randomization further confirmed that *DPEP1* and *CHMP1A* mediate the effect of genotype on kidney disease development^42^. Single-cell epigenome analysis, GWAS conditional analysis and genome editing studies defined that the expression of *DPEP1* and *CHMP1A* are controlled by a shared genomic region. Furthermore, *DPEP1* and *CHMP1A* expressions strongly correlates in human kidney tissue samples, further supporting their shared regulation in the human kidney. Our work indicates that a more comprehensive approach is needed for target variant and gene characterization.

As of now, almost all cell death pathways have been observed in mouse kidney injury models, including apoptosis, necroptosis, pyroptosis, and ferroptosis. Our results indicate converging evidence on ferroptosis as a key pathway in kidney function heritability. While injured kidneys and TECs showed enhanced pyroptosis and necroptosis, we observed minimal changes in these pathways in mice with genetic deletion of *Dpep1* or *Chmp1a*. Ferroptosis is a recently described form of regulated necrosis^36^. It is characterized by iron-catalyzed intracellular accumulation of lipid hydroperoxides. Ferroptosis is proposed to play a role in the pathogenesis of Huntington’s disease^44^, Parkinson’s disease^45^, hemochromatosis^46^, and acute kidney injury^47^. The abundance of polyunsaturated fatty acids, within the kidney tubular compartment^48^, likely contributes to the kidney’s high susceptibility to ferroptosis^49^. Our study now indicates the critical causal role of ferroptosis in CKD and kidney function regulation in patients as defined by kidney genetic studies.

We demonstrated that *Dpep1* and *Chmp1a* are important regulators of ferroptosis with opposing directions. Quantification of lipid peroxidation is an important feature of ferroptosis. Knockdown of *Dpep1* was associated with lower lipid peroxidation, whereas *Chmp1a* knockdown showed an increase in lipid peroxidation. ACSL4, which suppresses polyunsaturated fatty acid incorporation into phospholipid membranes, is another key player of ferroptosis^50–52^. We found that expression of *Acsl4* was lower in *Dpep1*^*+/−*^ when compared to wild-type after injury, whereas it was higher in *Chmp1a*^*+/*^^−^ heterozygous mice. Our results are consistent with previous publication showing that *ACSL4* expression correlates with renal function in patients with acute kidney tubular injury^53^. *DPEP1* expression is also strongly correlated with the ferroptosis marker *SLC3A2* in human microdissected kidney samples. In the kidney, both *SLC3A2/SLC7A11* and *SLC3A1/SLC7A9* are the transport system for cystine. *SLC3A1/SLC7A9* heterodimeric complex is present in the apical surface of renal proximal tubules. This system plays a role in cysteine reabsorption.

*CHMP1A* is a member of the ESCRT-III family that functions in the sorting of receptor proteins via the formation of endosomal MVBs^54^. Recently the role of the ESCRT-III in necroptosis^55^ and pyroptosis^56^ was proposed, however, we did not observe a marked alteration in these pathways in *Chmp1a* mutant mice. Treatment of *Chmp1a* heterozygous mice with liproxstatin offered marked protection from kidney disease, indicating the key role of ferroptosis in *Chmp1a-*mediated tubule injury in vivo. *Dpep1* is a membrane-bound glycoprotein responsible for hydrolyzing dipeptides. Here we show that *Dpep1* colocalized with endocytic vesicle marker clathrin and plays role in transferrin endocytosis. Loss of *Dpep1* protected from cisplatin-induced ferroptosis. *Dpep1* has also been reported to play roles in radiocontrast-induced kidney injury^33^ and cisplatin-induced tubule cell death^57^. Future studies shall determine the relative contribution of apoptosis and ferroptosis to DPEP1-afforded disease protection.

In conclusion, we show that a single fine-mapped GWAS locus controls the expression of two target genes using computational integration of GWAS, kidney mQTL, eQTL, single nuclei ATAC sequencing, and CRISPR-based genome editing. We identify *DPEP1* and *CHMP1A* as kidney disease genes and important regulators of ferroptosis. Our studies indicate that pharmacological targeting of ferroptosis through *Dpep1* or *Chmp1a* in kidney tubule cells could offer therapeutic benefits for patients with kidney disease.

## Methods

### GWAS, eQTL, mQTL, and moloc analysis

eGFR GWAS summary dataset was downloaded from CKDGen Consortium website (https://ckdgen.imbi.uni-freiburg.de/), and significant associations were defined by *P* value < 5.0E−8. Human kidney and glomerular eQTL dataset have been generated as previously published^40^. The DNA mQTL was performed on 188 human healthy kidney samples with genotyping and CpG methylation data following our prior publication^15^. Briefly human kidney samples were genotyped using Affymetrix Axiom arrays. Illumina EPIC arrays were used for methylation analysis. We conducted cis-mQTL (referred to as mQTL) association analysis using 188 samples with imputed genotyping data and methylation data by EPIC array. Beta values of each CpG were transformed by an inverse-normal transformation (INT). Missing values were imputed based on nearest neighbor averaging implemented by R package impute (v1.64.0). For each SNP-CpG pair within a cis window of ±1 Mb from the queried CpG site, the association between INT transformed methylation and genotype dosage was quantified using MatrixQTL (v2.1.0). R package using an additive linear model. This model was fitted with covariates including general variables (sample collection site, age, sex, top five genotype PCs, degree of bisulfite conversion, sample plate, and sentrix position) and PEER factors. Multiple trait colocalization among GWAS, eQTL, and mQTL was performed by moloc^58^. In short, SNPs+/− 100 kb of each leading GWAS SNPs were used to calculate the posterior probability. In the moloc results, PP_abc represents the posterior probability of three traits are associated and share a single causal variant. We used PP_abc > 0.8 as the threshold of multiple trait colocalization.

### Human kidney RNA-seq data

Gene expression changes were examined in previously published microdissected human kidney RNA sequencing data^59^ (*n* = 432). The clinical information is shown in Supplementary Data 3. The study was approved by the institutional review board of the University of Pennsylvania.

### Single-cell ATAC sequencing

We reanalyzed mouse snATAC-seq data (three healthy mouse kidneys; 16,887 nuclei) and human snATAC-seq data (two healthy human kidneys; 12,720 nuclei), as described earlier^41,60^.

### Conditional analysis

Each of the 12 open-chromatin peaks was evaluated for conditional analysis. SNPs within each peak were identified and combined into peak-specific lists. Peak-specific conditional analyses used the–cojo-cond command in GCTA, with the list of peak SNPs being input as the conditional SNP-list, with eGFR GWAS analysis^13^ results used as the input summary data, and BioVU imputed genetic data as the reference dataset for evaluating linkage disequilibrium. Output for each peak provides the conditional analysis results of all SNPs within the locus region after conditioning on all available SNPs within the open chromatin peak. When peak SNPs were in strong LD introducing collinearity, one SNP from each group was excluded from the analysis. Final included SNPs were assigned to peaks as follows (Peaks 2 and 7 did not contain any available SNPs):

Peak 1: rs8059821; rs80089054; rs4785697

Peak 3: rs111857923; rs187720

Peak 4: rs62068712; rs192325916

Peak 5: rs154665

Peak 6: rs11641525; rs258340; rs12930346; rs7197490; rs146442848

Peak 8: rs908951; rs11649482

Peak 9: rs142099578; rs12920969; rs4785581; rs1657380

Peak 10: rs164751; rs58290281; rs201976326; rs12921177

Peak 11: rs35415928; rs151272435; rs13329897

Peak 12: rs16965913; rs5818725; rs80164364; rs117418297; rs59863025

### CRISPR/Cas9 mediated peak deletion

HEK293 cell stably expressing Cas9 was a gift from Dr. Liling Wan from University of Pennsylvania. The sgRNA expression plasmids were generated as previously published^61^. Briefly, Annealed sgRNA oligos were subcloned into pLKO5.sgRNA.EFS.GFP with the Bsmb1 site. All constructs were verified by Sanger sequencing. sgRNA expression plasmids were transfected to Cas9 stable HEK293 cell using lipofectamine 3000. After 72 h, cells were harvested, and *Dpep1* and *Chmp1a* expression were determined by QRT-PCR. At the same time, DNA was isolated, and sgRNAs target regions were determined by Sanger sequencing. sgRNA sequences are listed in Supplementary Data 5.

### Mice

Eight- to ten-week-old male mice were used in this study. All mice were maintained under SPF conditions with ambient temperature 20–22, humidity 50–70%, and a 12/12 h light/dark cycle. All animal experiments were reviewed and approved by the Institutional Animal Care and Use Committee of the University of Pennsylvania and were performed in accordance with the institutional guidelines. *Dpep1* mutant mice were generated by co-injection of Cas9 mRNA (100 ng/μl; ThermoFisher, A29378), sgRNA (50 ng/μl) in CRISPR Cas9 Mouse Targeting Core of University of Pennsylvania. Two sgRNAs were generated with Guide-it™ sgRNA In Vitro Transcription Kit (Takara #632635). The sgRNA sequences and genotyping primers are listed in Supplementary Data 5. *Chmp1a* mutant mice were imported from Mutant Mouse Regional Resource Center of UC Davis (#031089-UCD). For FA-induced nephropathy mouse models, 8-week-old male wild-type and *Dpep1*^*+/−*^ or *Chmp1a*^*+/−*^ mice were injected with FA (250 or 200 mg/kg, dissolved in 300 mM sodium bicarbonate) intraperitoneally and euthanized on day 7. For the cisplatin-induced injury model, 8-week-old male wild-type, *Dpep1*^*+/−*^ or *Chmp1a*^*+/−*^ mice were injected with cisplatin (25 or 20 mg/kg) intraperitoneally. Mice were euthanized on day 3. For the UUO model, mice underwent ligation of the left ureter and were euthanized on day 7, and sham-operated mice were used as controls.

### BUN and creatinine level

Serum creatinine was measured by Creatinine Enzymatic and Creatinine Standard (DIAZYME #DZ072B-KY1). Serum BUN was measured by Infinity™ Urea Liquid Stable Reagent (Pointe Scientific #B7552150). Both measurements were performed according to the manufacturers’ instructions.

### Histopathology analysis

Kidneys were harvested from mice, rinsed in PBS, fixed in 10% formalin, and embedded in paraffin. Histological analysis was examined by H&E and Picrosirius red (Polyscience #24901). The acute tubular injury was scored as previously described^43^. In brief, semi-quantitation was evaluated, including tubular dilation, tubular atrophy, tubular cast formation, vacuolization, degeneration, using the following scoring system, Score 0: no tubular injury; Score 1: <10% of tubules injury; Score 2: 10–25% of tubules injury; Score 3: 25–50% of tubules injury; Score 4: 50–74% of tubules injury; Score 5: >75% of tubules injury.

### Real-time RT-PCR

RNA was isolated from mouse kidneys or cultured cells using Trizol reagent (Invitrogen) and was reverse transcribed into cDNA using cDNA Archival Kit (Life Technologies). Real-time RT-PCR was performed using SYBR Green Master Mix (Applied Biosystems). Primer pair sequences are shown in Supplementary Data 6.

### Cell culture

Rat epithelial cells (NRK-52E; ATCC (CRL-1571)) were cultured in DMEM with 5% fetal bovine serum (FBS). *Dpep1* and *Chmp1a* siRNA were purchased from Dharmacon. *Dpep1-*overexpressing vector pCMV6-Entry was purchased from OriGene Technologies, Inc. Transfection of gene-targeting siRNA, negative control siRNA, and overexpressing plasmid were performed using Lipofectamine 3000.

For transfection, cells were seeded in six-well plates, grown overnight until 60–70% confluency, and then transfected with 50 nM (final concentration) siRNA or 5 μg *Dpep1*-overexpression plasmids. Transfection efficiency was determined under a fluorescence microscope by the presence of Cy3 transfection control. After 24 h of *Chmp1a* siRNA transfection, cells were pretreated with the following inhibitors: 20 μM Nec-1 (Cayman #11658); 1 μM Liproxstatin1 (Cayman #17730); 10 μM VX-765 (Cayman # 28825); 20 μM Z-VAD(OMe)-FMK for 18 h and then cells were treated with 20 μM cisplatin (Cayman #15663-27-1) for 24 h. After 24 h of *Dpep1* siRNA transfection, cells were treated with cell death inducers for 18–20 h, respectively: 5 μM Nigericin (Sigma # N7143); 5 μM CPT (Cayman #11694); 5 μM erastin (Cayman #17754), 20 μM FIN56 (Cayman #25180), 5 μM FINO2 (Cayman #25096) and 5 μM RSL3 (Cayman #19288).

For primary culture of renal tubule cells, kidneys were collected from 2- to 4-week-old male mice. Cells were isolated by 2 mg/ml collagenase I (Gibco #17018-029) digestion for 30 min at 37 °C with gentle stirring and filtered through a 100-μm mesh. The cell suspension was cultured in RPMI 1640 (Corning #10-040-CM) supplement with 10% FBS (Atlanta Biologicals #S11950), 20 ng/ml EGF (Peprotech #AF-100-15), 1× ITS (Gibco #51500–056), and 1% penicillin-streptomycin (Corning #30-002-CI) at 5% CO_2_ and 37 °C. Primary renal tubule cells were pretreated with 50 μM Mito-TEMPO (Sigma #SML0737) for 1 h and treated with 20 μM cisplatin for 24 h.

### Western blot

Kidney tissue or cultured cell lysates were prepared with ice-cold lysis buffer (CST # 9806) containing protease inhibitor cocktail (cOmplete Mini, Roche #11836153001) and phosphatase inhibitor (PhosSTOP, Roche #4906837001), resolved on 8–12% gradient gels, transferred on to polyvinylidene difluoride membranes, and probed with the following antibodies: DPEP1 (Proteintech #12222-1-AP 1:500), CHMP1A (Proteintech #15761-1-AP 1:500), RIPK3 (Sigma #PRS2283 1:1000), Cleaved Caspase 1 (Santa cruz #sc-56036 1:500), Collagen III (Abcam #ab7778 1:1000), Fibronectin (Abcam #ab2413 1:1000), aSMA (Sigma #A5228 1:1000), ACSL4 (Abcam #ab155282 1:1000), CD63 (Abcam #ab193349 1:1000), GPX4 (Abcam #ab125066 1:1000), Actin (Sigma #A3854 1:20000), GAPDH (Proteintech #60004-1-Ig 1:1000), and Tubulin (BioLegend #801202 1:1000). Anti-rabbit IgG (H + L) (DyLight™ 800 4X PEG Conjugate) (CST #5151 1:10000) and Anti-mouse IgG (H + L) (DyLight™ 680 Conjugate 1:10000) (CST #5470) was used as a secondary antibody.

### Immunofluorescence

Cells were washed with PBS, fixed with 4% paraformaldehyde, permeabilized with PBS-0.2% Triton X 100, and blocked with 5% FBS. Immunostaining were performed using the following primary antibodies: Cleaved Caspase 3 (CST #9664 1:500), Fluorescein labeled LTL (Vector #FL-1321 1:500), AQP2 (Santa Cruz #sc-9882 1:200), Fluorescein labeled DBA (Vector #FL-1031-5 1:500), Fluorescein labeled PNA (Vector # FL-1071-5 1:500), DPEP1 (Invitrogen #PA5-52984 1:200), CHMP1A (Proteintech #15761-1-AP 1:200), EEA1 (BD#610456 1:200), RAB5 (CST #3547 1:200), RAB7 (Sigma #R8779 1:200), RAB11 (BD #610658 1:200), VAMP7 (NOVUS #NBP1-07118 1:200), and GM130 (BD #610822 1:200).

### Live cell imaging

Primary cultured renal tubule cells or NRK52E cells were incubated with 1 μM transferrin (Thermo Fisher #T13342) for 4 h, or 5 μM mitoSOX (ThermoFisher #M36008) for 10 min, or 2 μM BODIPY™ 581/591 C11 (Thermo Fisher #D3861) for 0.5 h, or 20 ng/ml dextran conjugated to 488 (Thermo Fisher #D22910) for 2 h, at 5% CO_2_ and 37 °C, respectively. After washing with PBS, cells were imaged directly under the confocal or regular fluorescence microscope.

### Cell count and cytotoxicity assays

Mouse primary tubular epithelial cells from wild-type, mutant mice or NRK52E cells were plated in 96-well plate. Transfection of NRK52E cells with siRNA was carried out as described above. After 24 h of transfection, cisplatin was added for 24 h. Cells were harvested and stained with trypan blue (Thermo Fisher #T10282) to visualize dead cells. Cell counts were analyzed in Countess Auto Counter (Invitrogen, C10227). LDH release was quantified using CytoTox 96 Non-Radioactive Cytotoxicity Assay (Promega #G1780). Medium was collected in triplicates, spun down, and incubated in a 96-well plate with the CytoTox reagent for 20–30 min. After adding the stop solution, the absorbance signal was measured at 490 nm in a plate reader. The ratio between dead cell and live cell was calculated through the measurement using MultiTox-Fluor Multiplex Cytotoxicity Assay (Promega #9201). Cells were incubated in assay buffer containing live cell substrate GF-AFC and dead cell substrate bis-AAF-R110 for 30 min at 37 °C. The plate fluorescence was measured using a plate reader as following: viability: excitation ~400 nm; emission ~505 nm. cytotoxicity: excitation ~485 nm; emission ~520 nm.

### TUNEL assay

Apoptotic cells in the kidney were detected by Click-iT^TM^ Plus TUNEL Assay for In Situ Apoptosis Detection, Alexa Fluor^TM^ 594 dye (ThermoFisher Scientific, #C10618) according to the manufacturer’s instruction. Images were obtained under fluorescence microscope. Apoptotic cells were quantified and presented as the number of TUNEL positive cells per field.

### Glutathione assay

The GSH and oxidized glutathione (GSSG) content in the kidney was measured by Glutathione Colorimetric Detection Kit (Invitrogen #EIAGSHC) according to the instruction of the manufacturer.

### Iron assay

Kidney tissue was weighed, cut, and lysed, and iron content was measured by Iron Colorimetric Assay Kit according to the manufacturers’ instructions (BioVision # K390-100). The iron content was normalized to the weight of the tissue (microgram of iron per gram of tissue).

### Statistics and reproducibility

Statistical analyses were performed using GraphPad Prism software (GraphPad Software Inc., La Jolla, CA). A two-tailed *t*-test was used to compare two groups. One-way or two-way ANOVA was used to compare multiple groups with post hoc Tukey test.

### Reporting summary

Further information on research design is available in the Nature Research Reporting Summary linked to this article.

## Supplementary information

Supplementary Information

Description of Additional Supplementary Files

Supplementary Data 1-6

Reporting Summary

## Data Availability

The RNA-seq data used in this study are available in the NCBI’s Gene Expression Omnibus database under accession code GSE115098. Mouse kidney snATAC-seq data used in this study are available in the NCBI’s Gene Expression Omnibus database under accession code GSE157079 and can be viewed on the Susztak Lab website (http://susztaklab.com/developing_adult_kidney/igv/). The precomputed human kidney eQTL data used in this study are available on the Susztak Lab website (http://www.susztaklab.com/eQTLci/download.php). The human kidney single-nuclei ATAC-seq data used in this study are available in the NCBI’s Gene Expression Omnibus database under accession code GSE172008 and can be viewed on the Susztak Lab website (http://www.susztaklab.com/human_kidney/igv/). Biochemical source data are provided in the Source Data file with this paper. Source data are provided with this paper.

## Source data

Source Data
