# Supplementary material for: A single genetic locus controls both expression of DPEP1/CHMP1A and kidney disease development via ferroptosis
Source: Nat Commun. 2021 Aug 23;12:5078. doi: 10.1038/s41467-021-25377-x (PMC8382756; doi:10.1038/s41467-021-25377-x)
Supplement: Supplementary file 1 — Supplementary Information [file 41467_2021_25377_MOESM1_ESM.pdf]

Supplementary Materials for

A single genetic locus controls both expression of *DPEP1/CHMP1A* and disease development via ferroptosis

Yuting Guan[1,2#], Xiujie Liang[1,2#], Ziyuan Ma[1,2], Hailong Hu[1,2], Hongbo Liu[1,2], Zhen Miao[1,2,3], Andreas Linkermann[4,5], Jacklyn N. Hellwege[6], Benjamin F. Voight[2,7,8], and Katalin Susztak[1,2,*]

Correspondence to: ksusztak@pennmedicine.upenn.edu

Figure S1

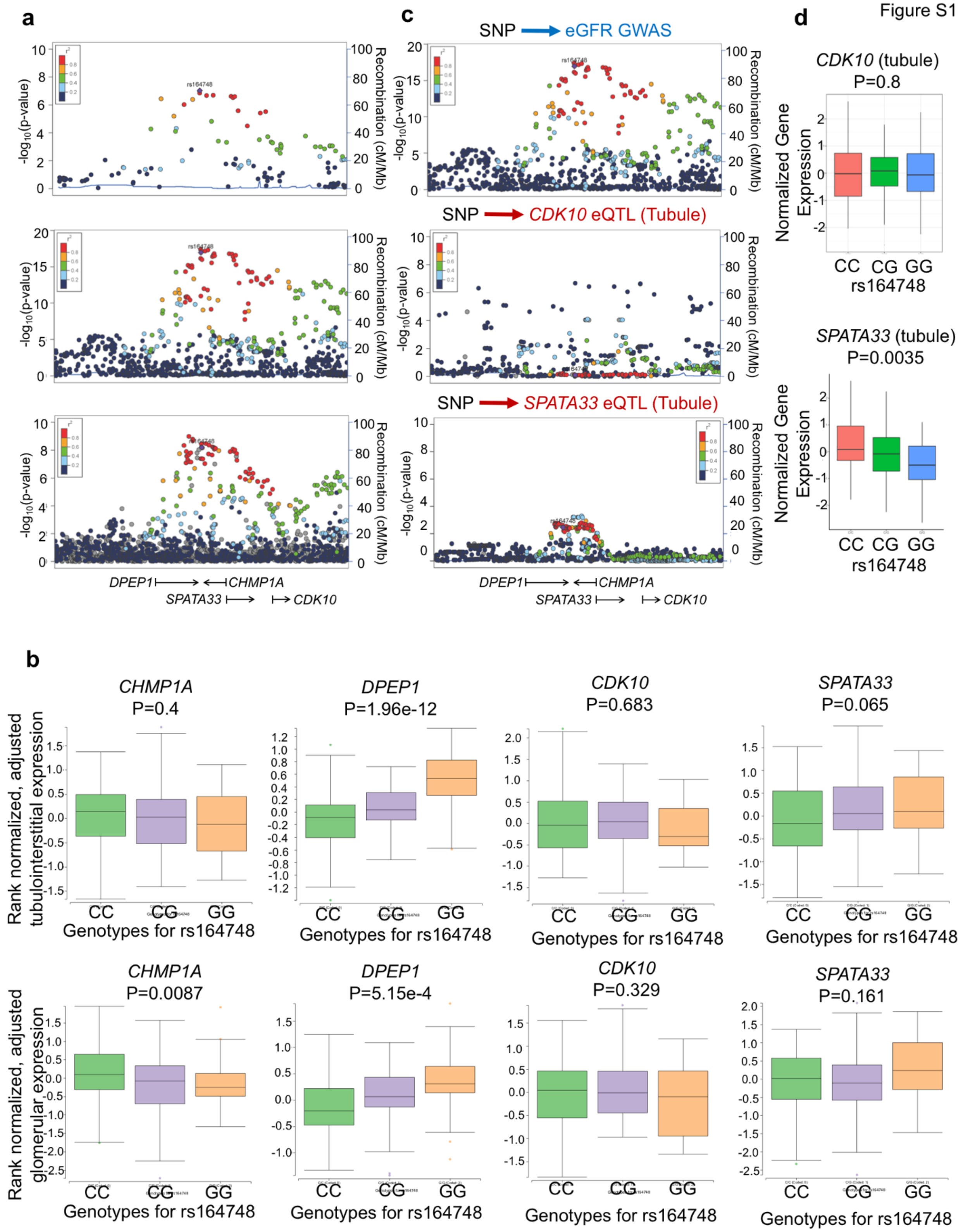
a
b
c
d
SNP → eGFR GWAS
SNP → CDK10 eQTL (Tubule)
SNP → SPATA33 eQTL (Tubule)
rs164748
-log10(p-value)
Recombination (cM/Mb)
DPEP1
CHMP1A
SPATA33
CDK10
CDK10 (tubule)
P=0.8
SPATA33 (tubule)
P=0.0035
Normalized Gene Expression
CC CG GG
CHMP1A
P=0.4
DPEP1
P=1.96e-12
CDK10
P=0.683
SPATA33
P=0.065
Rank normalized, adjusted tubulointerstitial expression
Genotypes for rs164748
CHMP1A
P=0.0087
DPEP1
P=5.15e-4
CDK10
P=0.329
SPATA33
P=0.161
Rank normalized, adjusted glomerular expression

Fig. S1. Genotype-phenotype (GWAS) and genotype-gene expression (eQTL) association analysis at the chromosome 16 eGFR GWAS locus.

a, LocusZoom plots of chromosome 16 region of eGFR GWAS, using the CKDGen 2016 release (upper), the CKDGen 2019 release (middle), and the Million Veteran Program (bottom).

b, The association of genotype (rs164748) and *CHMP1A, DPEP1, CDK10*, *SPATA33* expression in human tubules (n=166) and glomeruli (n=136) in the NephQTL database[1] (http://nephqtl.org). Centerlines show the medians; box limits indicate the 25th and 75th percentiles; whiskers extend to the 5th and 95th percentiles; outliers are represented by dots.

c, LocusZoom plots of eGFR GWAS (top), *CDK10* eQTL (middle) and *SPATA33* eQTL (bottom) analysis in tubules (n=121) around the region of rs164748. The *x-axis* shows the chromosomal location. The *y* axis shows –log10(*P*) of association tests (by linear regression).

d, The association of genotype (rs164748) and gene expression (*CDK10* and *SPATA33*) in human tubules (n=121) in the Susztak lab eQTL database (www.susztaklab.com). Centerlines show the medians; box limits indicate the 25th and 75th percentiles; whiskers extend to the 5th and 95th percentiles. P-value was calculated by linear regression.

Figure S2

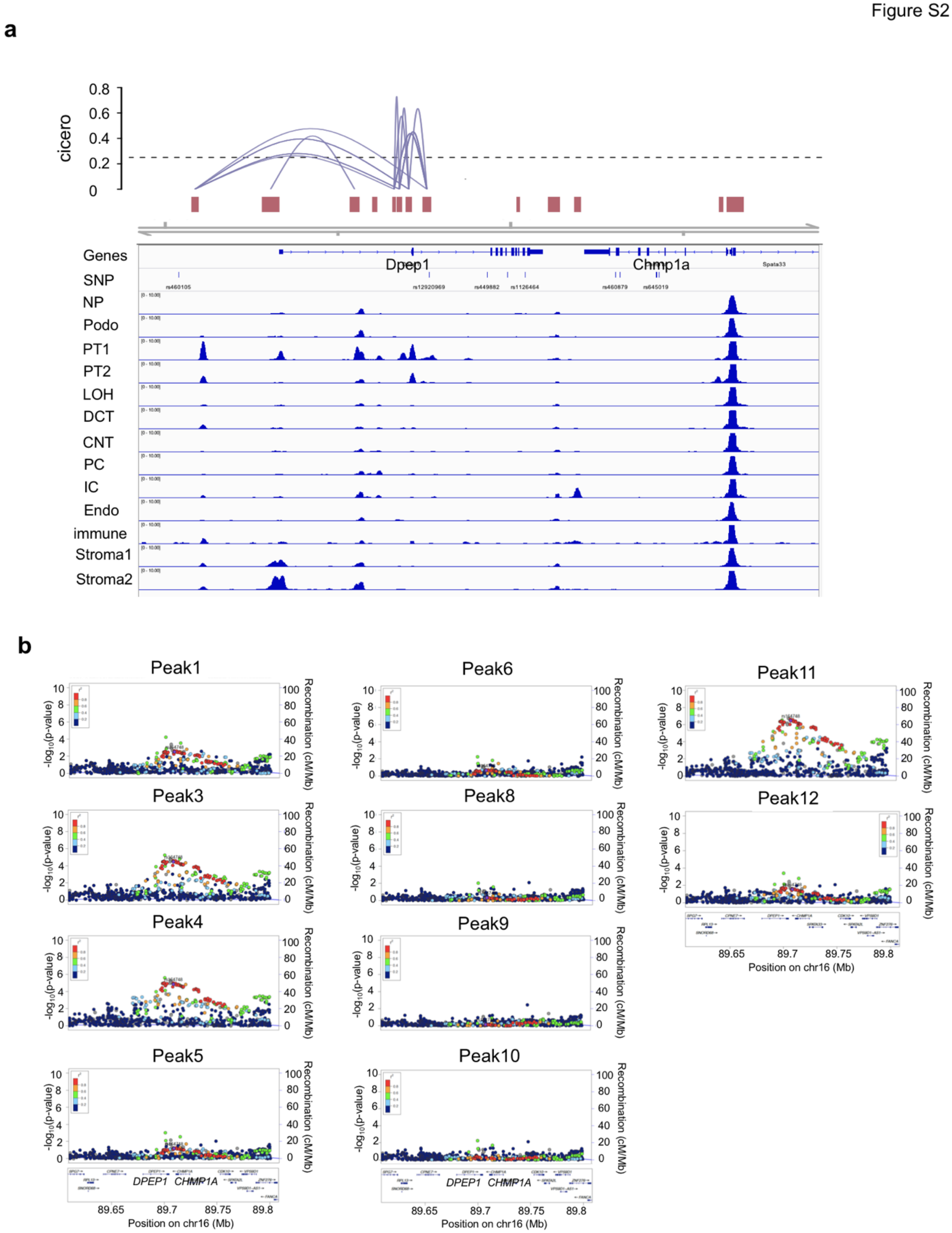
a
cicero
0.8
0.6
0.4
0.2
0
Genes
Dpep1
Chmp1a
Spata33
SNP
rs460105
rs12920969
rs449882
rs1126464
rs460879
rs645019
NP
Podo
PT1
PT2
LOH
DCT
CNT
PC
IC
Endo
immune
Stroma1
Stroma2
b
Peak1
Peak3
Peak4
Peak5
Peak6
Peak8
Peak9
Peak10
Peak11
Peak12
-log10(p-value)
Recombination (cM/Mb)
DPEP1 CHMP1A
89.65
89.7
89.75
89.8
Position on chr16 (Mb)

Fig. S2. Cicero-inferred co-accessibility of open chromatin regions in mouse *Dpep1* and *Chmp1a* locus and conditional eGFR GWAS analysis.

a, From top to bottom: (top) Cicero-inferred co-accessibility of open chromatin regions in adult mouse kidney. (bottom) Genome browser view of chromatin accessibility of nephron progenitors (NP), podocytes (Podo), proximal tubules segment 1 and 2 (PT S1 and PT S2), the loop of Henle (LOH), distal convoluted tubule (DCT), collecting duct principal cell types (PC), collecting duct intercalated cells (IC), endothelial cells (Endo), immune cells (Immune) and stromal cells (stroma).

b, LocusZoom plots of conditional eGFR GWAS analysis of snATAC-seq harbored multiple SNPs. Peak locations of 1-12 are shown in Figure 2a.

Figure S3

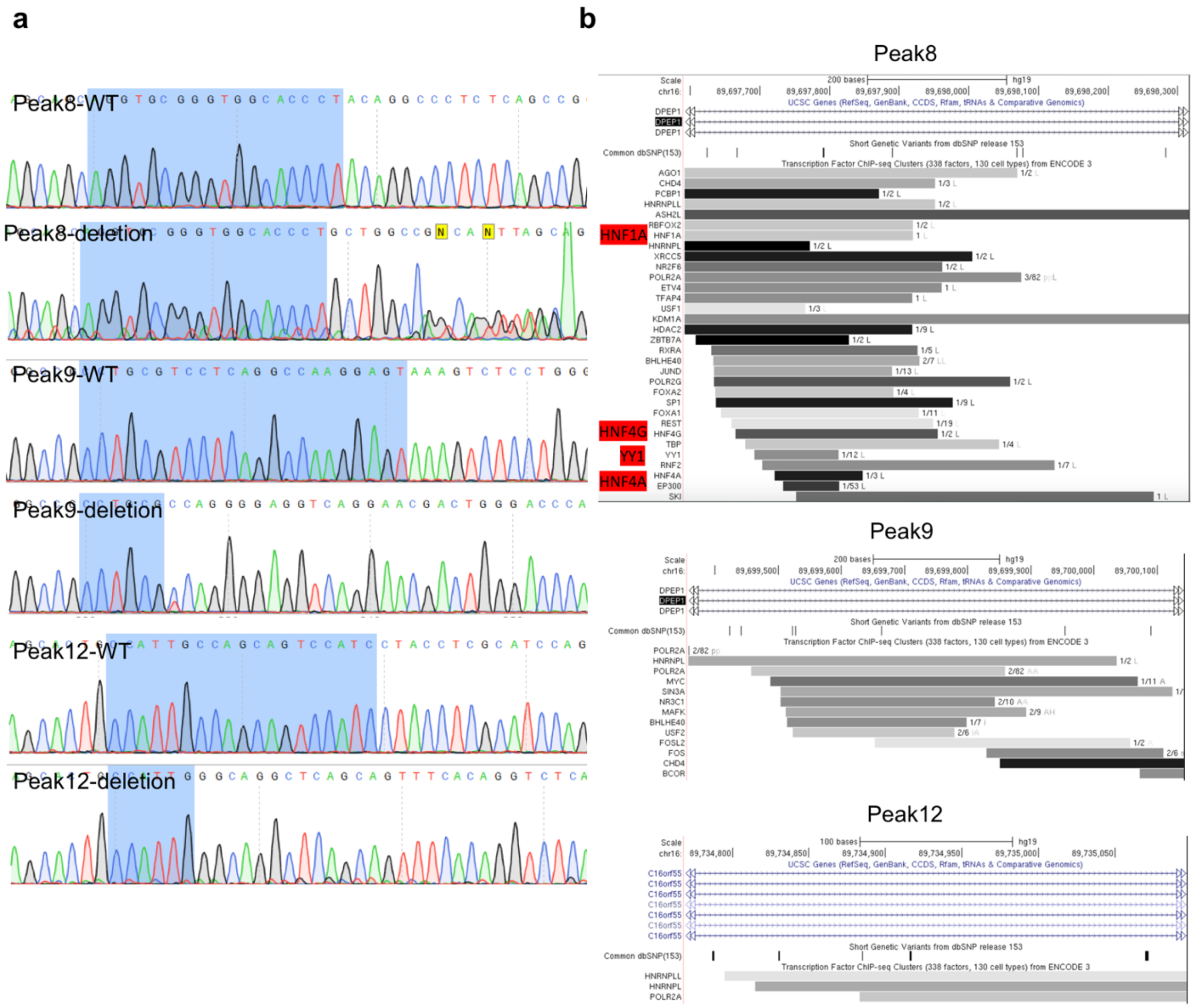
a
b
Peak8-WT
Peak8-deletion
Peak9-WT
Peak9-deletion
Peak12-WT
Peak12-deletion
Peak8
Peak9
Peak12
HNF1A
HNF4G
YY1
HNF4A

Fig. S3. Genome editing-based fine mapping of the kidney function GWAS region

a, CRISPR/Cas9 mediated deletion of the genomic region.

b, Genome Brower view of transcription factor binding in the genomic regions of 8, 9 and 12.

Figure S4

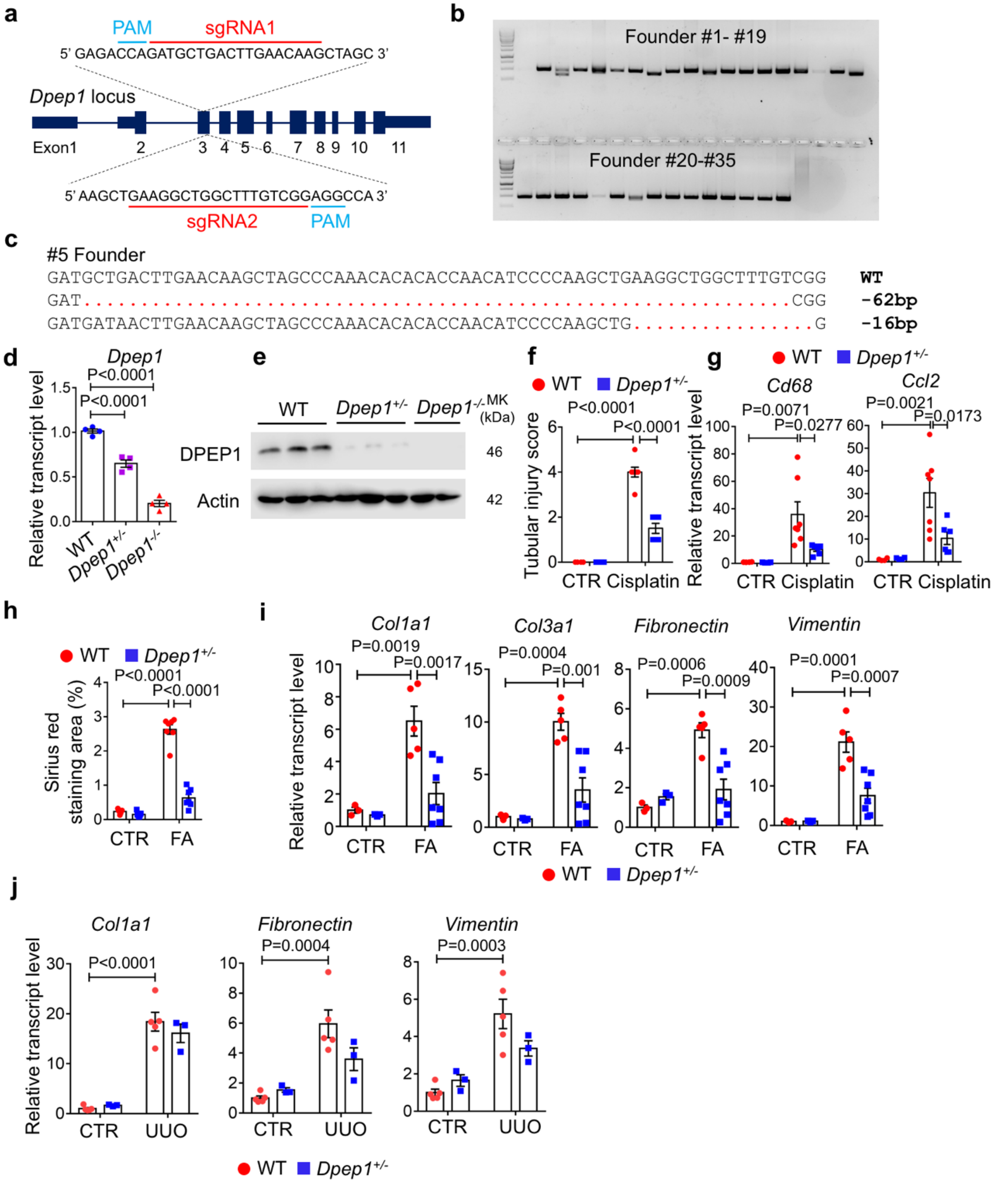

a
PAM
sgRNA1
5' GAGACCAGATGCTGACTTGAACAAGCTAGC 3'
Dpep1 locus
Exon1
2
3 4 5 6 7 8 9 10 11
5' AAGCTGAAGGCTGGCTTTGTCGGAGGCCA 3'
sgRNA2
PAM
b
Founder #1- #19
Founder #20-#35
c
#5 Founder
GATGCTGACTTGAACAAGCTAGCCCAAACACACACCAACATCCCCAAGCTGAAGGCTGGCTTTGTCGG WT
GAT.....CGG -62bp
GATGATAACTTGAACAAGCTAGCCCAAACACACACCAACATCCCCAAGCTG.....G -16bp
d
Dpep1
P<0.0001
P<0.0001
Relative transcript level
WT
Dpep1+/-
Dpep1-/-
e
WT
Dpep1+/-
Dpep1-/-
MK (kDa)
DPEP1
Actin
46
42
f
WT
Dpep1+/-
P<0.0001
P<0.0001
Tubular injury score
CTR Cisplatin
g
WT
Dpep1+/-
Cd68
Ccl2
P=0.0071
P=0.0277
P=0.0021
P=0.0173
Relative transcript level
CTR Cisplatin
CTR Cisplatin
h
WT
Dpep1+/-
P<0.0001
P<0.0001
Sirius red staining area (%)
CTR FA
i
Col1a1
Col3a1
Fibronectin
Vimentin
P=0.0019
P=0.0017
P=0.0004
P=0.001
P=0.0006
P=0.0009
P=0.0001
P=0.0007
Relative transcript level
CTR FA
WT
Dpep1+/-
j
Col1a1
Fibronectin
Vimentin
P<0.0001
P=0.0004
P=0.0003
Relative transcript level
CTR UUO
WT
Dpep1+/-

Fig. S4. Generation of *Dpep1* knock-out animals.

a, The guide RNA targeting region at the mouse *Dpep1* locus.

b, Electrophoresis image of *Dpep1* founder lines genotyping.

c, Nucleotide deletion information for the #5 founder line.

d, Relative mRNA level of *Dpep1* in kidneys of wildtype, *Dpep1* heterozygous and homozygous mice (n=4 per group).

e, Western blots of DPEP1 from kidneys of wildtype, *Dpep1* heterozygous and homozygous mice.

f, Tubular injury scores in kidneys of control and $Dpep1^{+/-}$ mice following sham or cisplatin injection. Sham-treated group: WT (n=4), $Dpep1^{+/-}$ (n=4); cisplatin-treated group: WT (n=7), $Dpep1^{+/-}$ (n=6).

g, Relative mRNA level of immune cell markers *Cd68* and *Ccl2* in the kidneys of control and $Dpep1^{+/-}$ mice following sham or cisplatin injection. Sham-treated group: WT (n=4), $Dpep1^{+/-}$ (n=4); cisplatin-treated group: WT (n=7), $Dpep1^{+/-}$ (n=6).

h, Quantification of Sirius Red-stained kidney sections from control and $Dpep1^{+/-}$ mice following sham or folic acid injection (n=7 per group).

i, Relative transcript levels of fibrosis markers in kidneys of control and $Dpep1^{+/-}$ mice following sham or folic acid injection. Sham-treated group: WT (n=3), $Dpep1^{+/-}$ (n=3); FA-treated group: WT (n=5), $Dpep1^{+/-}$ (n=7).

j, Relative mRNA levels of fibrosis markers in kidneys of control and $Dpep1^{+/-}$mice following sham or UUO procedure. Sham-treated group: WT (n=5), $Dpep1^{+/-}$ (n=5); UUO-treated group: WT (n=3), $Dpep1^{+/-}$ (n=3).

All data are represented as mean ± SEM. P-value was calculated by one-way or two-way ANOVA with post hoc Tukey test. $P<0.05$ is statistically significant.

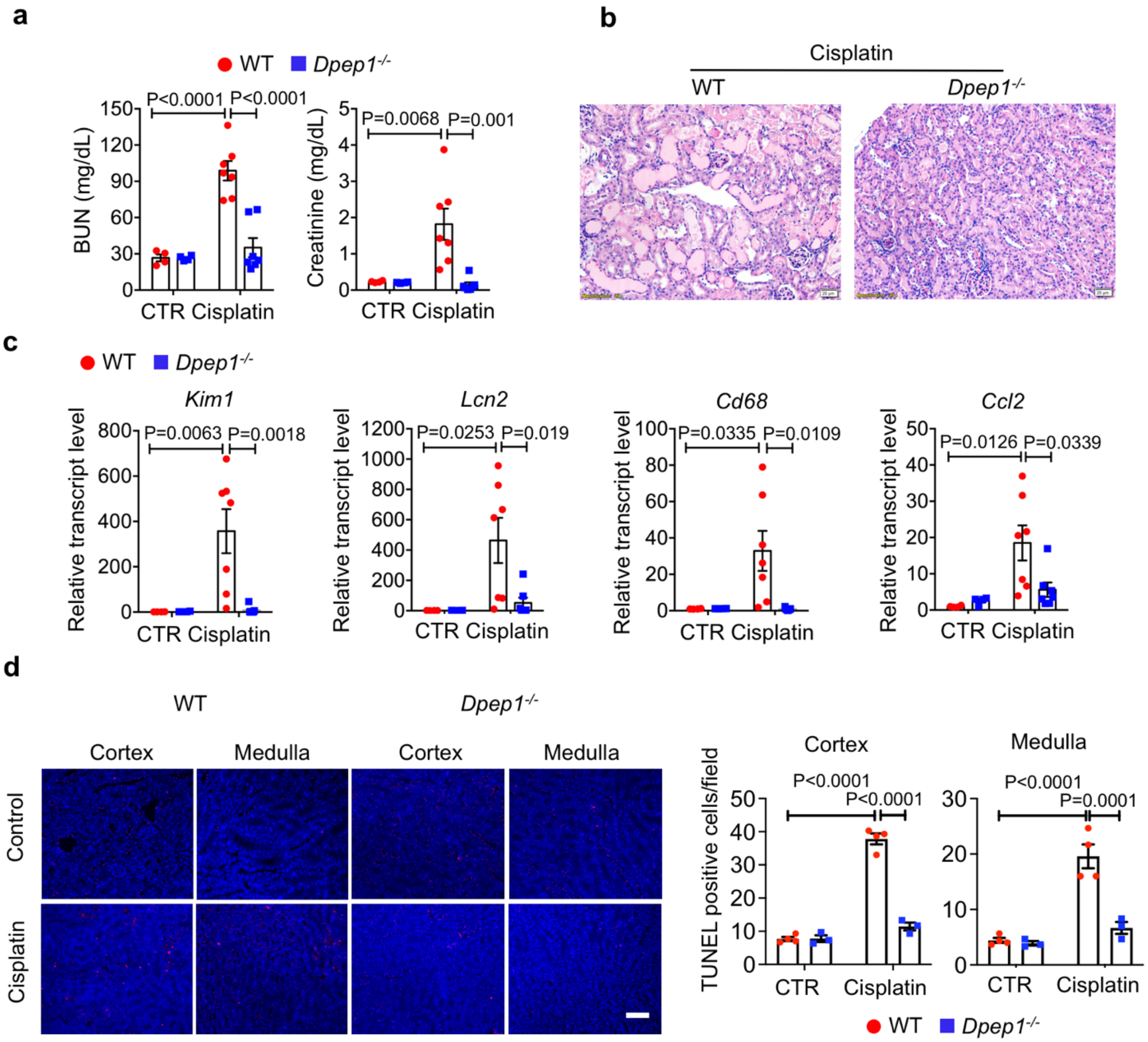
a
WT Dpep1-/-
BUN (mg/dL)
P<0.0001 P<0.0001
Creatinine (mg/dL)
P=0.0068 P=0.001
CTR Cisplatin
b
Cisplatin
WT
Dpep1-/-
c
WT Dpep1-/-
Kim1
Lcn2
Cd68
Ccl2
Relative transcript level
P=0.0063 P=0.0018
P=0.0253 P=0.019
P=0.0335 P=0.0109
P=0.0126 P=0.0339
CTR Cisplatin
d
WT
Dpep1-/-
Cortex
Medulla
Control
Cisplatin
TUNEL positive cells/field
P<0.0001
P<0.0001
P=0.0001
CTR Cisplatin
WT Dpep1-/-

Fig. S5. *Dpep1* knock-out mice are protected from cisplatin-induced renal injury.

a, Serum BUN and creatinine measurement of control and *Dpep1*$^{-/-}$ mice following sham or cisplatin injection. Sham-treated group: WT (n=4), *Dpep1*$^{-/-}$ (n=4); cisplatin-treated group: WT (n=7), *Dpep1*$^{-/-}$ (n=7).

b, Representative images of HE-stained kidney sections from control and *Dpep1*$^{-/-}$ mice following sham or cisplatin injection. Scale bar: 20 μm.

c, Relative mRNA level of injury markers *Kim1* and *Lcn2,* and immune cell markers *Cd68* and *Ccl2* in kidneys of control and *Dpep1*$^{-/-}$ mice following sham or cisplatin injection. Sham-treated group: WT (n=4), *Dpep1*$^{-/-}$ (n=4); cisplatin-treated group: WT (n=7), *Dpep1*$^{-/-}$ (n=7).

d, TUNEL staining of kidney sections of control and *Dpep1*$^{-/-}$ mice following sham or cisplatin injection. Quantification of TUNEL positive cells per field in cortex and medulla (n=3 per group). Scale bar: 20 μm.

All data are represented as mean ± SEM. P-value was calculated two-way ANOVA with post hoc Tukey test. P<0.05 is statistically significant.

Figure S6

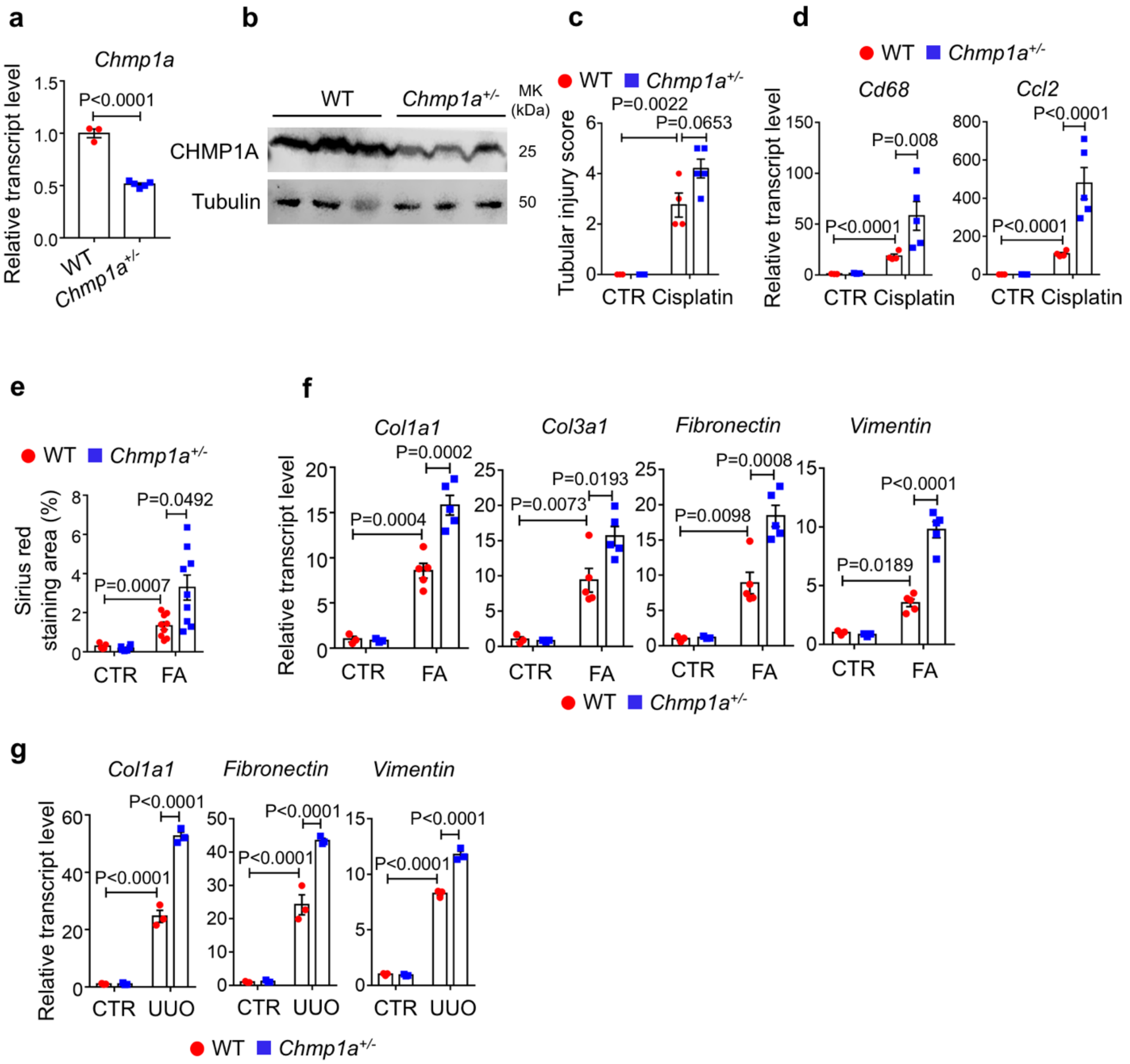
a
b
c
d
Chmp1a
Relative transcript level
P<0.0001
WT
Chmp1a+/-
CHMP1A
Tubulin
MK (kDa)
25
50
WT ■ Chmp1a+/-
Tubular injury score
P=0.0022
P=0.0653
CTR Cisplatin
Cd68
Ccl2
P=0.008
P<0.0001
e
f
Sirius red staining area (%)
P=0.0492
P=0.0007
CTR FA
Col1a1
Col3a1
Fibronectin
Vimentin
P=0.0002
P=0.0004
P=0.0193
P=0.0073
P=0.0008
P=0.0098
P<0.0001
P=0.0189
g
CTR UUO

Fig.S6. *Chmp1a* haploinsufficiency exacerbates kidney diseases.

a, Relative mRNA level of *Chmp1a* in kidney tissue of wildtype (n=3) and *Chmp1a*$^{+/-}$ mice (n=5).

b, Western blots of CHMP1A of kidney tissue of wildtype and *Chmp1a*$^{+/-}$ mice.

c, Tubular injury scores of kidney sections of control and *Chmp1a*$^{+/-}$ mice following sham or cisplatin injection. Sham-treated group: WT (n=3), *Chmp1a*$^{+/-}$ (n=3); cisplatin-treated group: WT (n=4), *Chmp1a*$^{+/-}$ (n=5).

d, Relative transcript levels of immune cell markers *Cd68* and *Ccl2* in kidneys of control and *Chmp1a*$^{+/-}$ mice following sham or cisplatin injection. Sham-treated group: WT (n=3), *Chmp1a*$^{+/-}$ (n=3); cisplatin-treated group: WT (n=4), *Chmp1a*$^{+/-}$ (n=5).

e, Quantification of Sirius Red-stained kidney sections of control and *Chmp1a*$^{+/-}$ mice following sham or folic acid injection. Sham-treated group: WT (n=5), *Chmp1a*$^{+/-}$ (n=6); FA-treated group: WT (n=9), *Chmp1a*$^{+/-}$ (n=9).

f, Relative transcript levels of fibrosis markers in kidneys of control and *Chmp1a*$^{+/-}$ mice following sham or folic acid injection. Sham-treated group: WT (n=3), *Chmp1a*$^{+/-}$ (n=3); FA-treated group: WT (n=5), *Chmp1a*$^{+/-}$ (n=5).

g, Relative transcript levels of fibrosis markers in kidneys of control and *Chmp1a*$^{+/-}$ mice following sham or UUO procedure (n=3 per group).

All data are represented as mean ± SEM. P-value was calculated by two-tailed t-test or two-way ANOVA with post hoc Tukey test. $P<0.05$ is statistically significant.

Figure S7

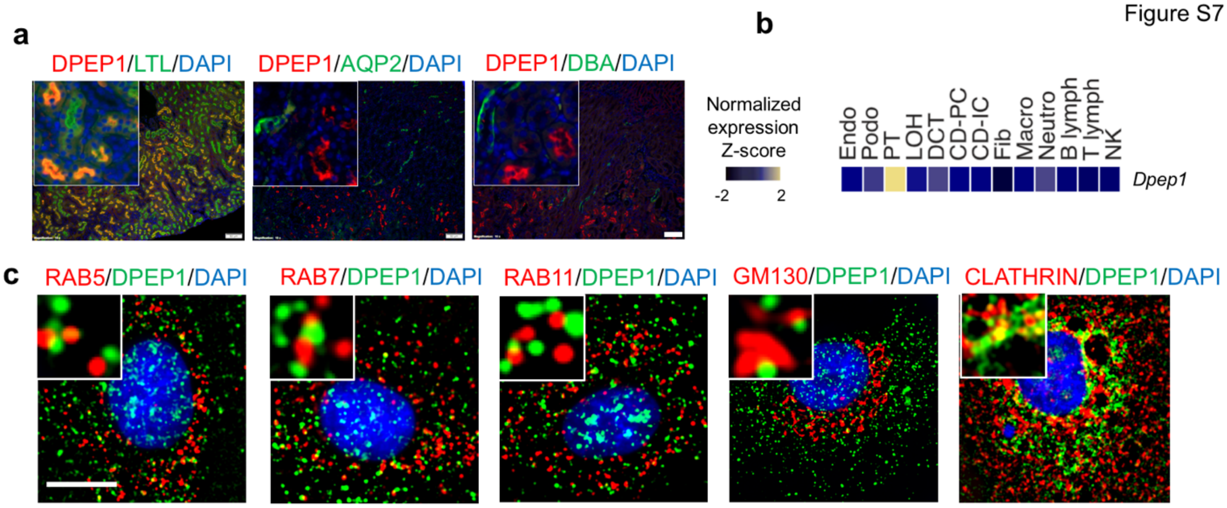
a
DPEP1/LTL/DAPI
DPEP1/AQP2/DAPI
DPEP1/DBA/DAPI
b
Normalized expression Z-score
-2
2
Endo
Podo
PT
LOH
DCT
CD-PC
CD-IC
Fib
Macro
Neutro
B lymph
T lymph
NK
Dpep1
c
RAB5/DPEP1/DAPI
RAB7/DPEP1/DAPI
RAB11/DPEP1/DAPI
GM130/DPEP1/DAPI
CLATHRIN/DPEP1/DAPI

Fig. S7. Characterization of DPEP1 expression in mouse kidney tissue and cells.

a, Representative images of double staining of DPEP1 and kidney segment markers LTL, AQP2, or DBA in healthy mouse kidney samples. Scale bar: 50 μm.

b, Relative *Dpep1* expression in mouse kidney single-cell RNA sequencing dataset.

c, Representative images of double staining of DPEP1 and early endosome marker RAB5, late endosome marker RAB7, recycling endosome marker RAB11, Golgi marker GM130, and coated vesicles marker clathrin in rat epithelial NRK52E cell. Scale bar: 10 μm.

Figure S8

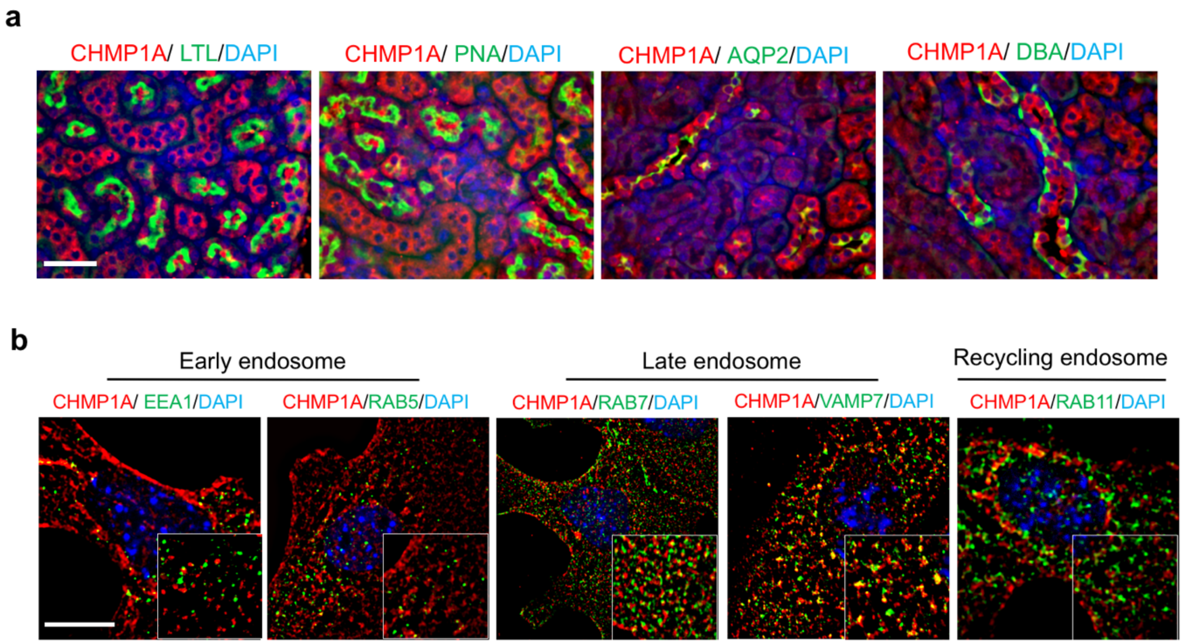
a
CHMP1A/ LTL/DAPI
CHMP1A/ PNA/DAPI
CHMP1A/ AQP2/DAPI
CHMP1A/ DBA/DAPI
b
Early endosome
Late endosome
Recycling endosome
CHMP1A/ EEA1/DAPI
CHMP1A/RAB5/DAPI
CHMP1A/RAB7/DAPI
CHMP1A/VAMP7/DAPI
CHMP1A/RAB11/DAPI

Fig. S8. Characterization of CHMP1A expression in kidney tissue samples and cells.

a, Representative images of double staining of CHMP1A and kidney segment markers LTL, PNA, AQP2, or DBA in healthy mouse kidney samples. Scale bar: 20 μm.

b, Representative images of double staining of CHMP1A and early endosome marker EEA1, RAB5, late endosome marker RAB7 and VAMP7, and recycling endosome marker RAB11 in rat epithelial NRK52E cell. Scale bar: 10 μm.

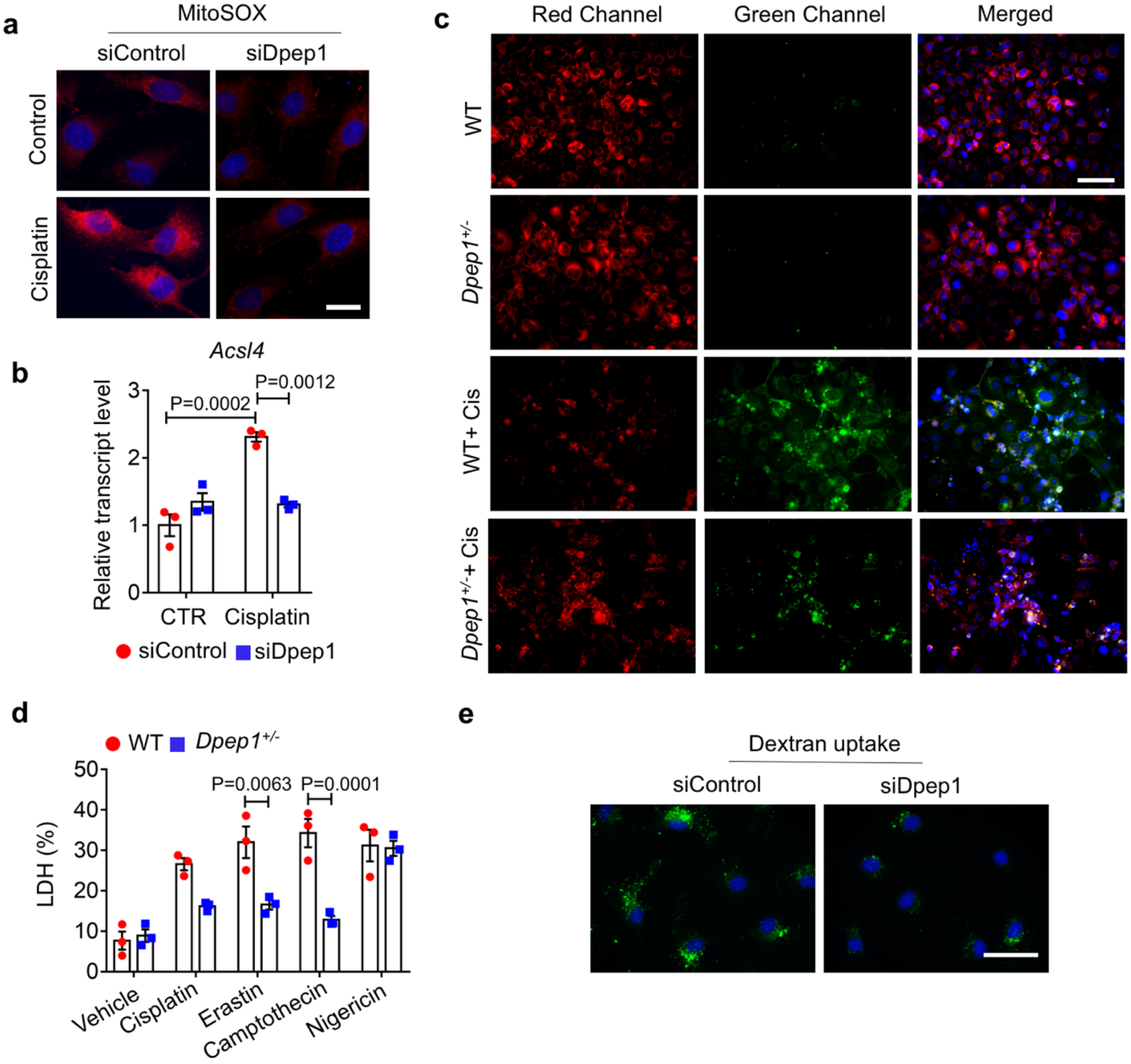

a
MitoSOX
siControl
siDpep1
Control
Cisplatin
b
Acsl4
Relative transcript level
P=0.0002
P=0.0012
CTR
Cisplatin
siControl
siDpep1
c
Red Channel
Green Channel
Merged
WT
Dpep1+/-
WT+ Cis
Dpep1+/- + Cis
d
WT
Dpep1+/-
LDH (%)
P=0.0063
P=0.0001
Vehicle
Cisplatin
Erastin
Camptothecin
Nigericin
e
Dextran uptake
siControl
siDpep1

Fig. S9. *Dpep1* knockdown is associated with lower lipid peroxidation

a, Representative mitoSOX staining of siControl and siDpep1 transfected cells following sham or cisplatin treatment. Scale bar: 10 µm.

b, Relative mRNA level of *Acsl4* from siControl and siDpep1 transfected cell following sham or cisplatin treatment (n=3).

c, Representative BODIPY 581/591 C11 staining of wildtype and wildtype and *Dpep1*$^{+/-}$ primary kidney tubule cell following sham or cisplatin treatment. Scale bar: 50 µm.

d, LDH level of wildtype and *Dpep1*$^{+/-}$ primary kidney tubule cell with or without cisplatin, erastin, camptothecin, and nigericin treatment (n=3).

e, Representative images of dextran uptake from siControl and siDpep1 transfected cells. Scale bar: 50 µm.

All data are represented as mean ± SEM. P-value was calculated by two-way ANOVA with post hoc Tukey test. $P<0.05$ is statistically significant.

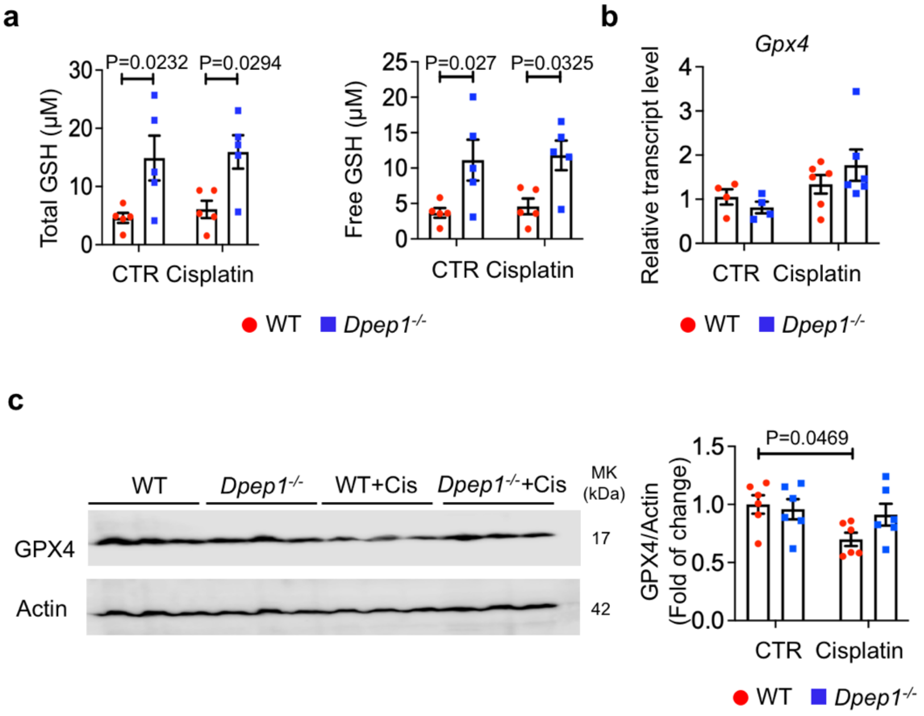
a
Total GSH (µM)
P=0.0232
P=0.0294
CTR Cisplatin
Free GSH (µM)
P=0.027
P=0.0325
CTR Cisplatin
WT
Dpep1-/-
b
Gpx4
Relative transcript level
CTR Cisplatin
WT
Dpep1-/-
c
WT
Dpep1-/-
WT+Cis
Dpep1-/-+Cis
MK (kDa)
GPX4
17
Actin
42
P=0.0469
GPX4/Actin (Fold of change)
CTR Cisplatin
WT
Dpep1-/-

Fig. S10. The effect of *Dpep1* loss on GSH and GPX4 levels.

a, The total and free GSH concentration in kidneys of wildtype and *Dpep1*$^{-/-}$ mice treated with or without cisplatin (n=5 per group).

b, Relative mRNA level of *Gpx4* in kidneys of control and *Dpep1*$^{-/-}$ mice following sham or cisplatin injection. Sham-treated group: WT (n=4), *Dpep1*$^{-/-}$ (n=4); cisplatin-treated group: WT (n=6), *Dpep1*$^{-/-}$ (n=6).

c, Western blots of GPX4 in kidneys of control and *Dpep*$^{-/-}$ mice following sham or cisplatin injection (n=6 per group).

All data are represented as mean ± SEM. P-value was calculated by two-way ANOVA with a post hoc Tukey test. P<0.05 is statistically significant.

Figure S11

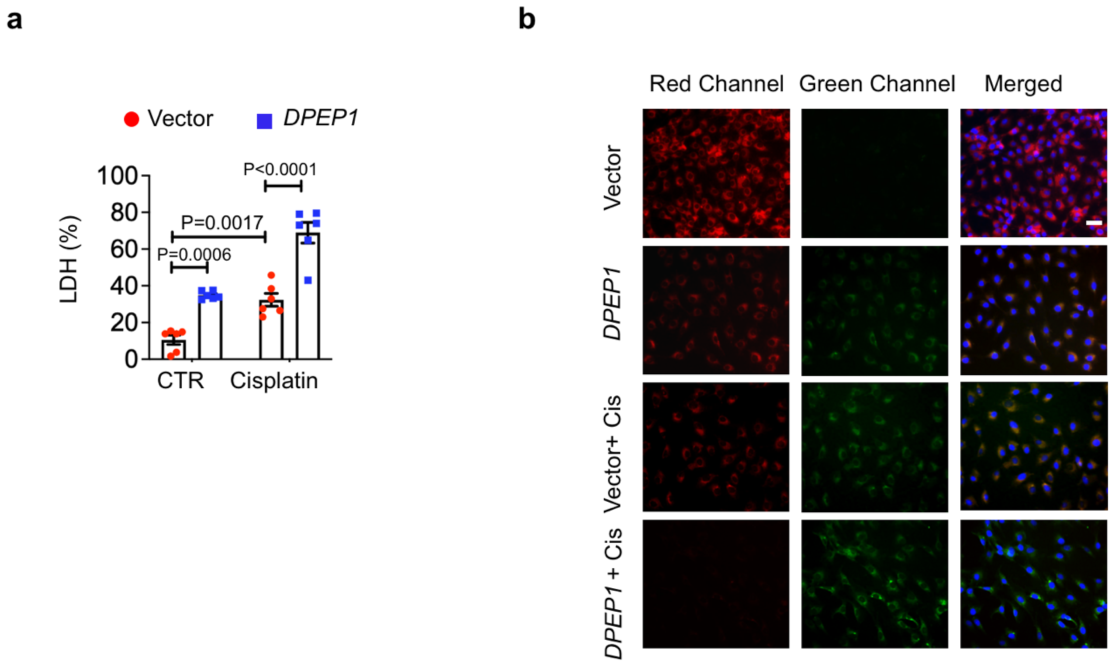
a
Vector
DPEP1
P<0.0001
P=0.0017
P=0.0006
LDH (%)
100
80
60
40
20
0
CTR
Cisplatin
b
Red Channel
Green Channel
Merged
Vector
DPEP1
Vector+ Cis
DPEP1 + Cis

Fig. S11. Overexpression of *DPEP1* is cytotoxic to kidney tubule cells.

a, LDH level of vector-transfected and *Dpep1*-overpressing vector-transfected cell with or without cisplatin treatment (n=6).

b, Representative BODIPY 581/591 C11 labeling of vector-transfected and *Dpep1*-overpressing vector-transfected cell with or without cisplatin treatment. Scale bar: 20 μm.

All data are represented as mean ± SEM. P-value was calculated by two-way ANOVA with post hoc Tukey test. $P<0.05$ is statistically significant.

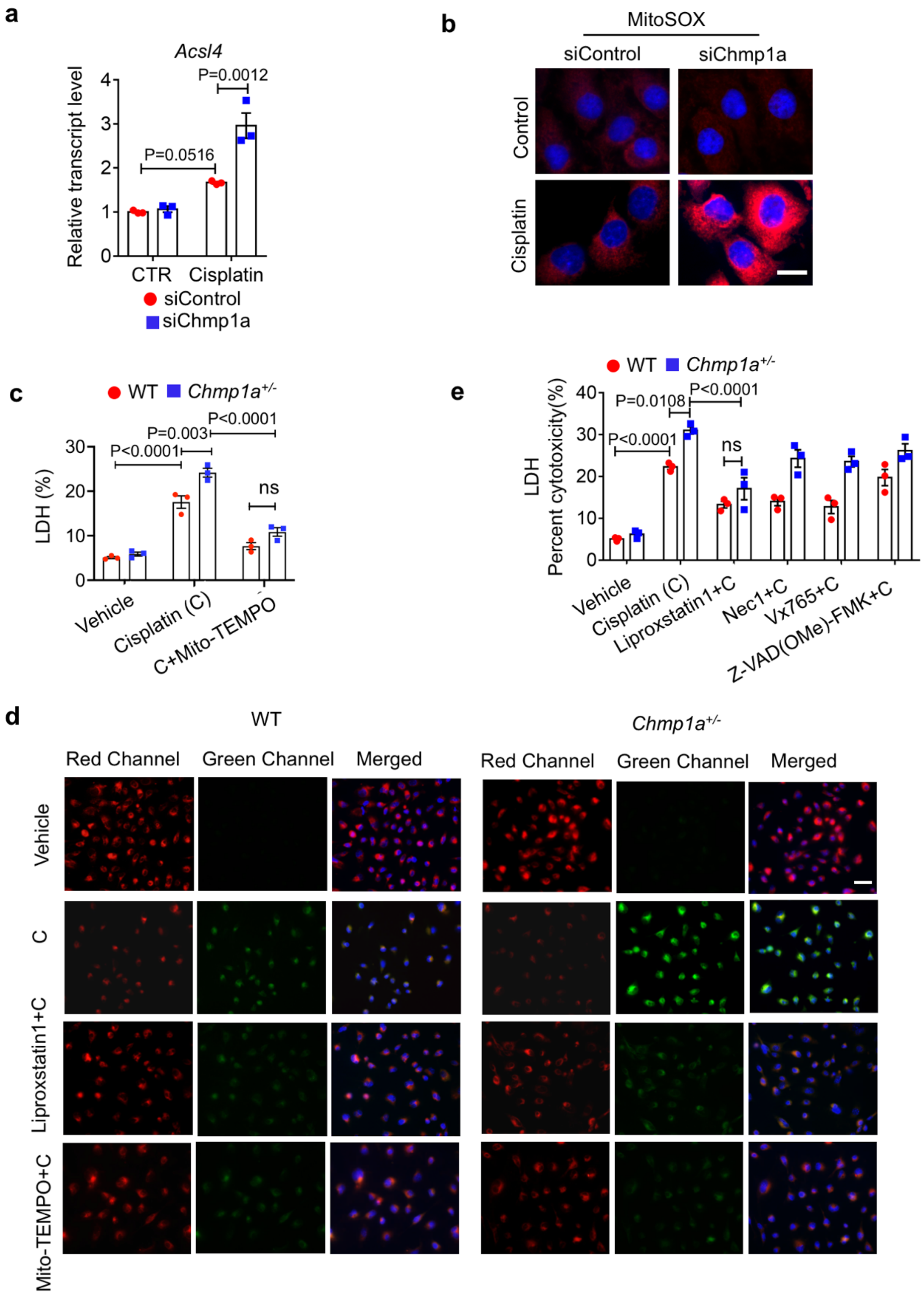

a
Acsl4
Relative transcript level
P=0.0012
P=0.0516
CTR
Cisplatin
siControl
siChmp1a
b
MitoSOX
siControl
siChmp1a
Control
Cisplatin
c
WT
Chmp1a+/-
LDH (%)
P<0.0001
P=0.003
P<0.0001
ns
Vehicle
Cisplatin (C)
C+Mito-TEMPO
e
WT
Chmp1a+/-
LDH
Percent cytotoxicity(%)
P=0.0108
P<0.0001
P<0.0001
ns
Vehicle
Cisplatin (C)
Liproxstatin1+C
Nec1+C
Vx765+C
Z-VAD(OMe)-FMK+C
d
WT
Chmp1a+/-
Red Channel
Green Channel
Merged
Vehicle
C
Liproxstatin1+C
Mito-TEMPO+C

Fig. S12 Haploinsufficiency of *Chmp1a* increases cytotoxicity of kidney tubule cells.

a, Relative mRNA level of *Acsl4* in siControl and siChmp1a transfected tubule cell following sham or cisplatin treatment (n=3).

b, Representative mitoSOX staining of siControl and siChmp1a transfected tubule cell following sham or cisplatin treatment. Scale bar: 10 μm.

c, LDH level of primary kidney tubule cells isolated from wildtype and *Chmp1a*$^{+/-}$ mice treated with or without Mito-TEMPO in the presence or absence of cisplatin (n=3).

d, Representative BODIPY 581/591 C11 labeling of primary tubule cells isolated from wildtype and *Chmp1a*$^{+/-}$ mice following sham or cisplatin treatment with or without liproxstatin1 or Mito-TEMPO. Scale bar: 20 μm.

e, LDH level of primary kidney tubule cells isolated from wildtype and *Chmp1a*$^{+/-}$ mice treated with or without cisplatin, erastin, camptothecin, and nigericin (n=3).

All data are represented as mean ± SEM. P-value was calculated by two-way ANOVA with a post hoc Tukey test. P<0.05 is statistically significant.

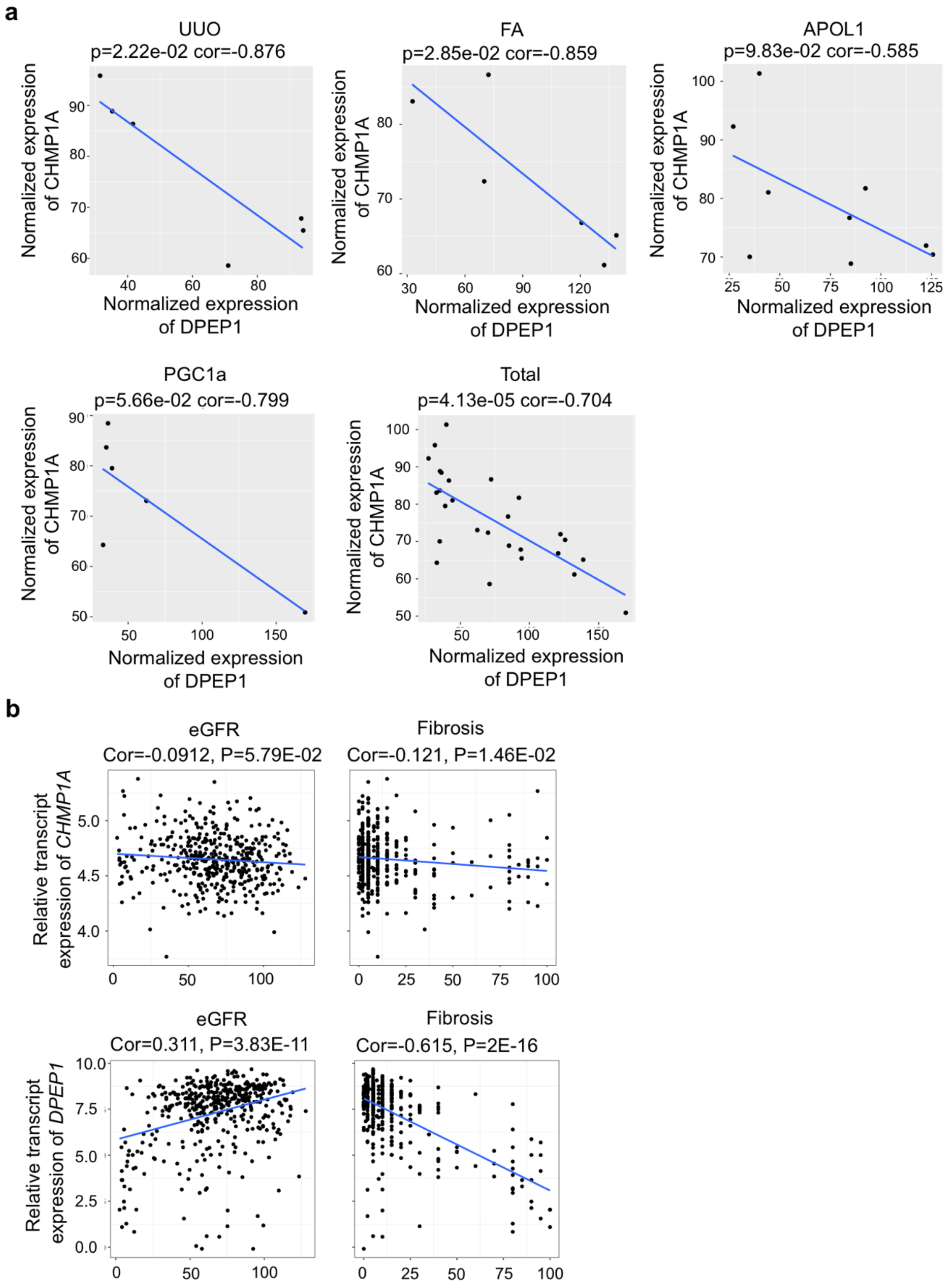
a
UUO
p=2.22e-02 cor=-0.876
FA
p=2.85e-02 cor=-0.859
APOL1
p=9.83e-02 cor=-0.585
PGC1a
p=5.66e-02 cor=-0.799
Total
p=4.13e-05 cor=-0.704
Normalized expression of CHMP1A
Normalized expression of DPEP1
b
eGFR
Cor=-0.0912, P=5.79E-02
Fibrosis
Cor=-0.121, P=1.46E-02
Relative transcript expression of CHMP1A
eGFR
Cor=0.311, P=3.83E-11
Fibrosis
Cor=-0.615, P=2E-16
Relative transcript expression of DPEP1

Fig. S13. The expression of *DPEP1* and *CHMP1A* in mouse kidney samples.

a, Relative expression of *Dpep1* (x-axis) and *Chmp1a* (y-axis) in kidneys of control and UUO, FA, APOL1, and PGC1a mouse kidney disease models (as analyzed by RNAseq). Pearson correlation is shown. Student t-test based on the Pearson correlation coefficient was used to calculate the statistical significance of the association.

b, Relative transcript levels of *CHMP1A* and *DPEP1* (y-axis), and kidney function (eGFR, x-axis) kidney fibrosis (x-axis) as analyzed in 432 microdissected human kidney samples. Pearson correlation is shown. Student t-test based on the Pearson correlation coefficient was used to calculate the statistical significance of the association.

Figure S14

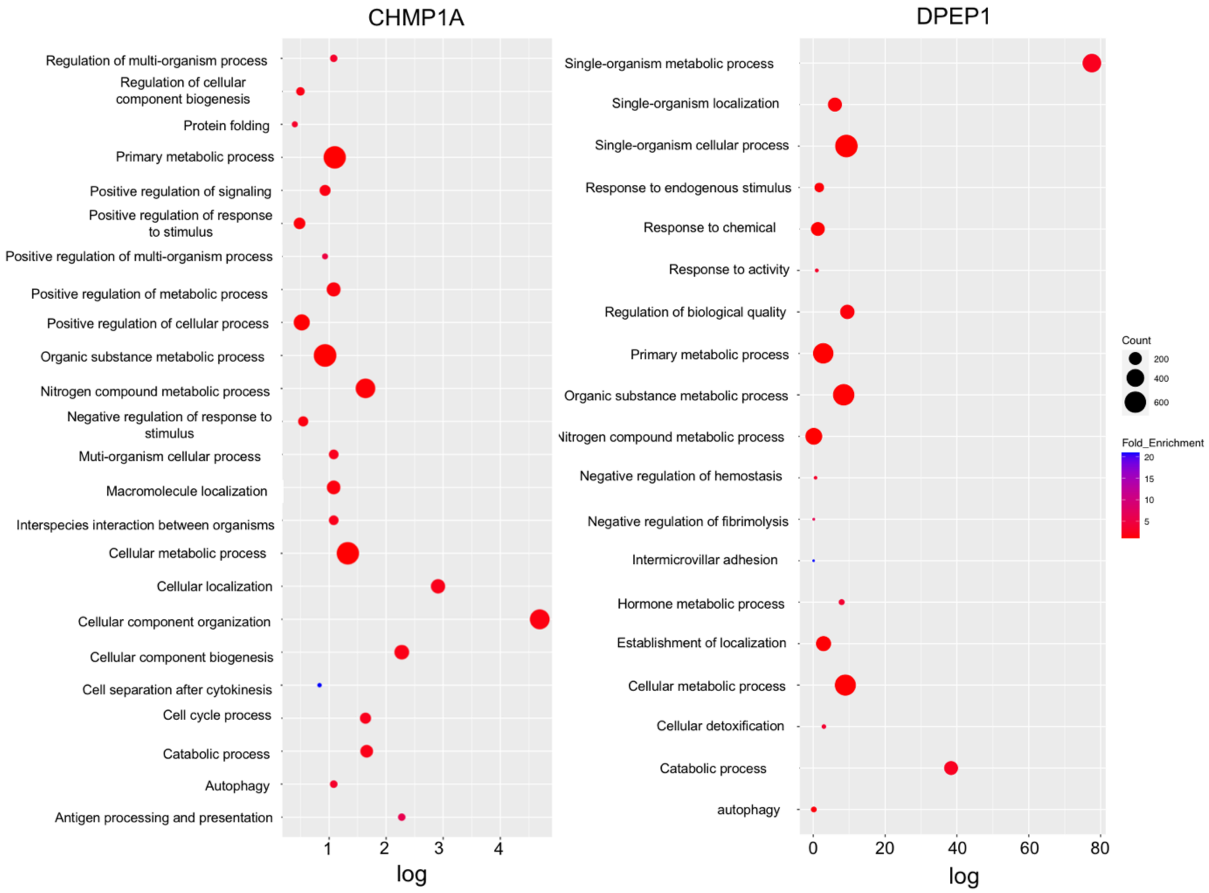

CHMP1A
Regulation of multi-organism process
Regulation of cellular component biogenesis
Protein folding
Primary metabolic process
Positive regulation of signaling
Positive regulation of response to stimulus
Positive regulation of multi-organism process
Positive regulation of metabolic process
Positive regulation of cellular process
Organic substance metabolic process
Nitrogen compound metabolic process
Negative regulation of response to stimulus
Muti-organism cellular process
Macromolecule localization
Interspecies interaction between organisms
Cellular metabolic process
Cellular localization
Cellular component organization
Cellular component biogenesis
Cell separation after cytokinesis
Cell cycle process
Catabolic process
Autophagy
Antigen processing and presentation
1
2
3
4
log
DPEP1
Single-organism metabolic process
Single-organism localization
Single-organism cellular process
Response to endogenous stimulus
Response to chemical
Response to activity
Regulation of biological quality
Primary metabolic process
Organic substance metabolic process
Nitrogen compound metabolic process
Negative regulation of hemostasis
Negative regulation of fibrimolysis
Intermicrovillar adhesion
Hormone metabolic process
Establishment of localization
Cellular metabolic process
Cellular detoxification
Catabolic process
autophagy
0
20
40
60
80
log
Count
200
400
600
Fold_Enrichment
20
15
10
5

Fig. S14. Gene ontology analysis (DAVID) of genes whose levels correlated with *CHMP1A* and *DPEP1* in human kidney samples.

Genes that were correlated with *CHMP1A* and *DPEP1* expression level in the RNA-seq data of 432 microdissected human kidney samples (Correlation coefficient >0.6) were subjected to Gene ontology analysis (DAVID).

Reference

1. Gillies, C.E. *et al.* An eQTL Landscape of Kidney Tissue in Human Nephrotic Syndrome. *Am J Hum Genet* **103**, 232-244 (2018).
