# Supplementary material for: A single genetic locus controls both expression of DPEP1/CHMP1A and kidney disease development via ferroptosis
Source: Nat Commun. 2021 Aug 23;12:5078. doi: 10.1038/s41467-021-25377-x (PMC8382756; doi:10.1038/s41467-021-25377-x)
Supplement: Supplementary file 2 — Description of Additional Supplementary Files [file 41467_2021_25377_MOESM2_ESM.pdf]

Description of Additional Supplementary Files

File Name: Supplementary Data 1
Description: Kidney-specific mQTLs associated with eGFR GWAS SNP rs164748

File Name: Supplementary Data 2
Description: Genotype and gene expression association in GTEx database

File Name: Supplementary Data 3
Description: Demographic and clinical data of human kidney samples

File Name: Supplementary Data 4
Description: Gene list that are correlated with CHMP1A and DPEP1 in human kidney samples.

File Name: Supplementary Data 5
Description: sgRNA and identification primer sequence

File Name: Supplementary Data 6
Description: qPCR primer sequences
