# Supplementary material for: A single genetic locus controls both expression of DPEP1/CHMP1A and kidney disease development via ferroptosis
Source: Nat Commun. 2021 Aug 23;12:5078. doi: 10.1038/s41467-021-25377-x (PMC8382756; doi:10.1038/s41467-021-25377-x)
Supplement: Supplementary file 4 — Reporting Summary [file 41467_2021_25377_MOESM4_ESM.pdf]

nature portfolio

Corresponding author(s): Katalin Susztak

Last updated by author(s): Jul 15, 2021

Reporting Summary

Nature Portfolio wishes to improve the reproducibility of the work that we publish. This form provides structure for consistency and transparency in reporting. For further information on Nature Portfolio policies, see our Editorial Policies and the Editorial Policy Checklist.

Statistics

For all statistical analyses, confirm that the following items are present in the figure legend, table legend, main text, or Methods section.

n/a	Confirmed	
☐	☒	The exact sample size (n) for each experimental group/condition, given as a discrete number and unit of measurement
☐	☒	A statement on whether measurements were taken from distinct samples or whether the same sample was measured repeatedly
☐	☒	The statistical test(s) used AND whether they are one- or two-sided<br>*Only common tests should be described solely by name; describe more complex techniques in the Methods section.*
☐	☒	A description of all covariates tested
☐	☒	A description of any assumptions or corrections, such as tests of normality and adjustment for multiple comparisons
☐	☒	A full description of the statistical parameters including central tendency (e.g. means) or other basic estimates (e.g. regression coefficient) AND variation (e.g. standard deviation) or associated estimates of uncertainty (e.g. confidence intervals)
☐	☒	For null hypothesis testing, the test statistic (e.g. F, t, r) with confidence intervals, effect sizes, degrees of freedom and P value noted<br>*Give P values as exact values whenever suitable.*
☒	☐	For Bayesian analysis, information on the choice of priors and Markov chain Monte Carlo settings
☒	☐	For hierarchical and complex designs, identification of the appropriate level for tests and full reporting of outcomes
☐	☒	Estimates of effect sizes (e.g. Cohen's d, Pearson's r), indicating how they were calculated

Our web collection on statistics for biologists contains articles on many of the points above.

Software and code

Policy information about availability of computer code

Data collection	qRT-PCR data were collected by Applied Biosystems ViiA7. Western blot images were collected by Odyssey Fc. Bright field and fluorescent images were collected by fluorescent microscope (Olympus BX43F) and confocal microscope (Zeiss LSM81). Absorbance signal was collected by plate reader (BioTek Synergy H1)
Data analysis	MatrixQTL (v2.1.0), ChromHMM (v1.15), LocusZoom (v0.4.8), GraphPad Prism (v6.0), Image J (v1.49), Cicero (v. 1.5.5), Harmony (v. 1.0), IGV (v. 2.8.9), MACS2 (v. 2.2.6), R (v1.64.0), Seurat (v. 3.0)

For manuscripts utilizing custom algorithms or software that are central to the research but not yet described in published literature, software must be made available to editors and reviewers. We strongly encourage code deposition in a community repository (e.g. GitHub). See the Nature Portfolio guidelines for submitting code & software for further information.

Data

Policy information about availability of data

All manuscripts must include a data availability statement. This statement should provide the following information, where applicable:

- Accession codes, unique identifiers, or web links for publicly available datasets
- A description of any restrictions on data availability
- For clinical datasets or third party data, please ensure that the statement adheres to our policy

The RNA-Seq data used in this study are available in the NCBI's Gene Expression Omnibus database under accession code GSE115098 (https://www.ncbi.nlm.nih.gov/geo/query/acc.cgi?acc=GSE115098). Mouse kidney snATAC-Seq data used in this study are available in the NCBI's Gene Expression Omnibus database under accession code GSE157079 (https://www.ncbi.nlm.nih.gov/geo/query/acc.cgi?acc=GSE157079) and can be viewed on the Susztak Lab website (http://susztaklab.com/developing_adult_kidney/igv/). The precomputed human kidney eQTL data used in this study are available in the NCBI's Gene Expression

Omnibus database under accession code GSE115098 (https://www.ncbi.nlm.nih.gov/geo/query/acc.cgi?acc=GSE115098) and can be viewed on the Susztak Lab website (http://www. susztaklab.com/eQTLci/download.php). The human kidney single-nuclei ATAC-Seq data used in this study are available in the NCBI's Gene Expression Omnibus database under accession code GSE172008 (https://www.ncbi.nlm.nih.gov/geo/query/acc.cgi?acc=GSE172008) and can be viewed on the Susztak Lab website (http://www.susztaklab.com/human_kidney/igv/.). Source data are provided with this paper.

Field-specific reporting

Please select the one below that is the best fit for your research. If you are not sure, read the appropriate sections before making your selection.

- [x] Life sciences
- [ ] Behavioural & social sciences
- [ ] Ecological, evolutionary & environmental sciences

For a reference copy of the document with all sections, see nature.com/documents/nr-reporting-summary-flat.pdf

Life sciences study design

All studies must disclose on these points even when the disclosure is negative.

Sample size	No sample size calculation was performed. For the animal experiment, sample size are determined based on the means and variation of previous pilot and published experiments (PMID: 30275566, 33441424). For cell and biochemical data, we aimed to collect data from at least three biological replicates.
Data exclusions	No data were excluded in the study.
Replication	All attempts at replication were successful. Independent repeated times for each experiment were indicated in the figure ledgends.
Randomization	All samples were randomly distributed into experimental group.
Blinding	Blinding was not relevant with this type of analysis, we collected samples that were available to us. Investigators were blinded to allocation during experiments and outcome assessments.

Reporting for specific materials, systems and methods

We require information from authors about some types of materials, experimental systems and methods used in many studies. Here, indicate whether each material, system or method listed is relevant to your study. If you are not sure if a list item applies to your research, read the appropriate section before selecting a response.

Materials & experimental systems

n/a	Involved in the study	
[ ]	[x]	Antibodies
[ ]	[x]	Eukaryotic cell lines
[x]	[ ]	Palaeontology and archaeology
[ ]	[x]	Animals and other organisms
[x]	[ ]	Human research participants
[x]	[ ]	Clinical data
[x]	[ ]	Dual use research of concern

Methods

n/a	Involved in the study	
[x]	[ ]	ChIP-seq
[x]	[ ]	Flow cytometry
[x]	[ ]	MRI-based neuroimaging

Antibodies

Antibodies used	DPEP1 (Invitrogen #PA5-52984), DPEP1(Proteintech # 12222-1-AP)CHMP1A (Proteintech #15761-1-AP), RIPK3 (Sigma #PRS2283), Cleaved Caspase1 (Santa cruz #sc-56036), Collagen III (Abcam #ab7778), Fibronectin (Abcam #ab2413), aSMA (Sigma #A5228), ACSL4 (Abcam #ab155282), CD63 (Abcam #ab193349), GPX4 (Abcam #ab125066), ACTIN (Sigma #A3854), GAPDH (Proteintech #60004-1-Ig) and TUBULIN (BioLegend #801202). Cleaved Caspase-3 (CST #9664), Fluorescein labeled Lotus Tetragonolobus Lectin (LTL) (Vector #FL-1321), AQP2 (Santa Cruz #sc-9882), Fluorescein labeled Dolichos Biflorus Agglutinin (DBA) (Vector #FL-1031-5), Fluorescein labeled Peanut Agglutinin (PNA) (Vector # FL-1071-5), EEA1 (BD#610456), RAB5 (CST #3547), RAB7 (Sigma #R8779), RAB11 (BD #610658), VAMP7 (NOVUS #NBP1-07118), GM130 (BD #610822). Anti-rabbit IgG (H+L) (DyLight™ 800 4X PEG Conjugate) (CST #5151) and Anti-mouse IgG (H+L) (DyLight™ 680 Conjugate) (CST #5470) was used as a secondary antibody.
Validation	All antibodies were previously validated by the manufacturer. Validation statement are provided online at vendors' websites. We used the primary antibodies according to the user manuals and got similar results as the vendors' websites.

Eukaryotic cell lines

Policy information about cell lines

Cell line source(s)	Primary TECs were isolated from mouse kidneys. Rat epithelial cell line NRK52E and HEK293T cells were from ATCC.

Authentication	Culture protocols of Primary TECs and NRK52E were previously validated in our lab (PMID: 30226866, 30275566).
Mycoplasma contamination	Primary TECs were not tested for mycoplasma contamination. NRK52E and HEK293T were negative for mycoplasma contamination when tested.
Commonly misidentified lines (See ICLAC register)	This study did not involve misidentified lines.

Animals and other organisms

Policy information about studies involving animals; ARRIVE guidelines recommended for reporting animal research

Laboratory animals	8- to 10-week-old male mice were used in this study. All mice were maintained under SPF conditions with ambient temperature 20-22, humidity 50-70% and a 12/12h light/dark cycle.
Wild animals	This study did not involve wild animals.
Field-collected samples	This study did not involve samples collected from the field.
Ethics oversight	The animal experiments were reviewed and approved by the Institutional Animal Care and Use Committee (IACUC) of the University of Pennsylvania in accordance with the guidelines of the National Institutes of Health.

Note that full information on the approval of the study protocol must also be provided in the manuscript.
