# Supplementary material for: A single genetic locus controls both expression of DPEP1/CHMP1A and kidney disease development via ferroptosis
Source: Nat Commun. 2021 Aug 23;12:5078. doi: 10.1038/s41467-021-25377-x (PMC8382756; doi:10.1038/s41467-021-25377-x)

Figure 3f aSMA

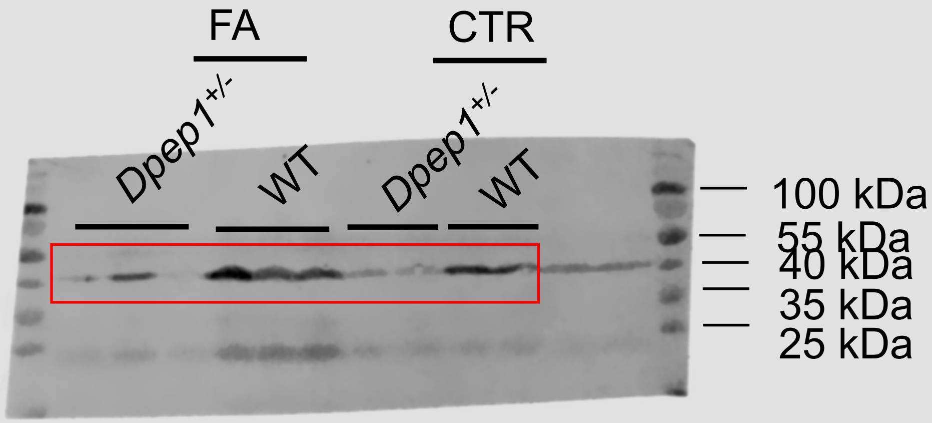

Figure 3f actin

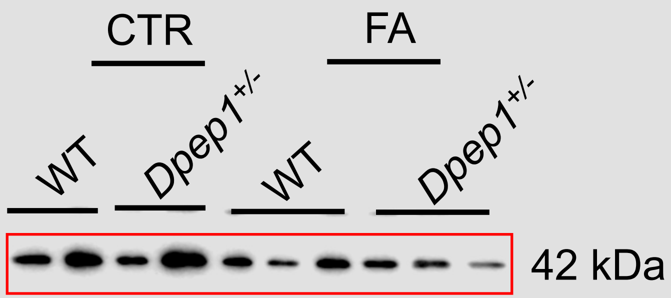

Figure 3l aSMA

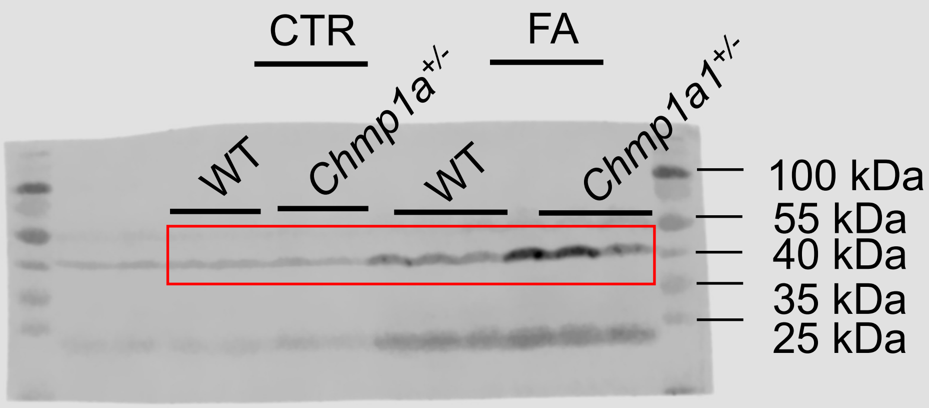

Figure 3l actin

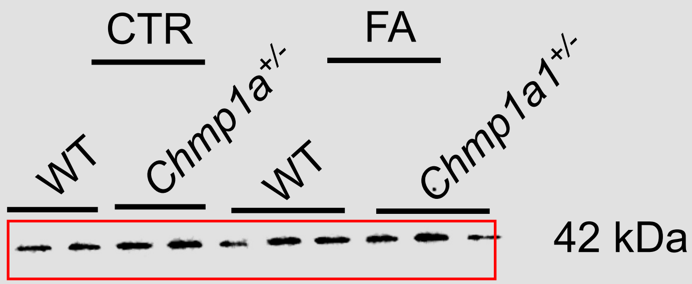

Figure 3f FN

Figure 3l FN

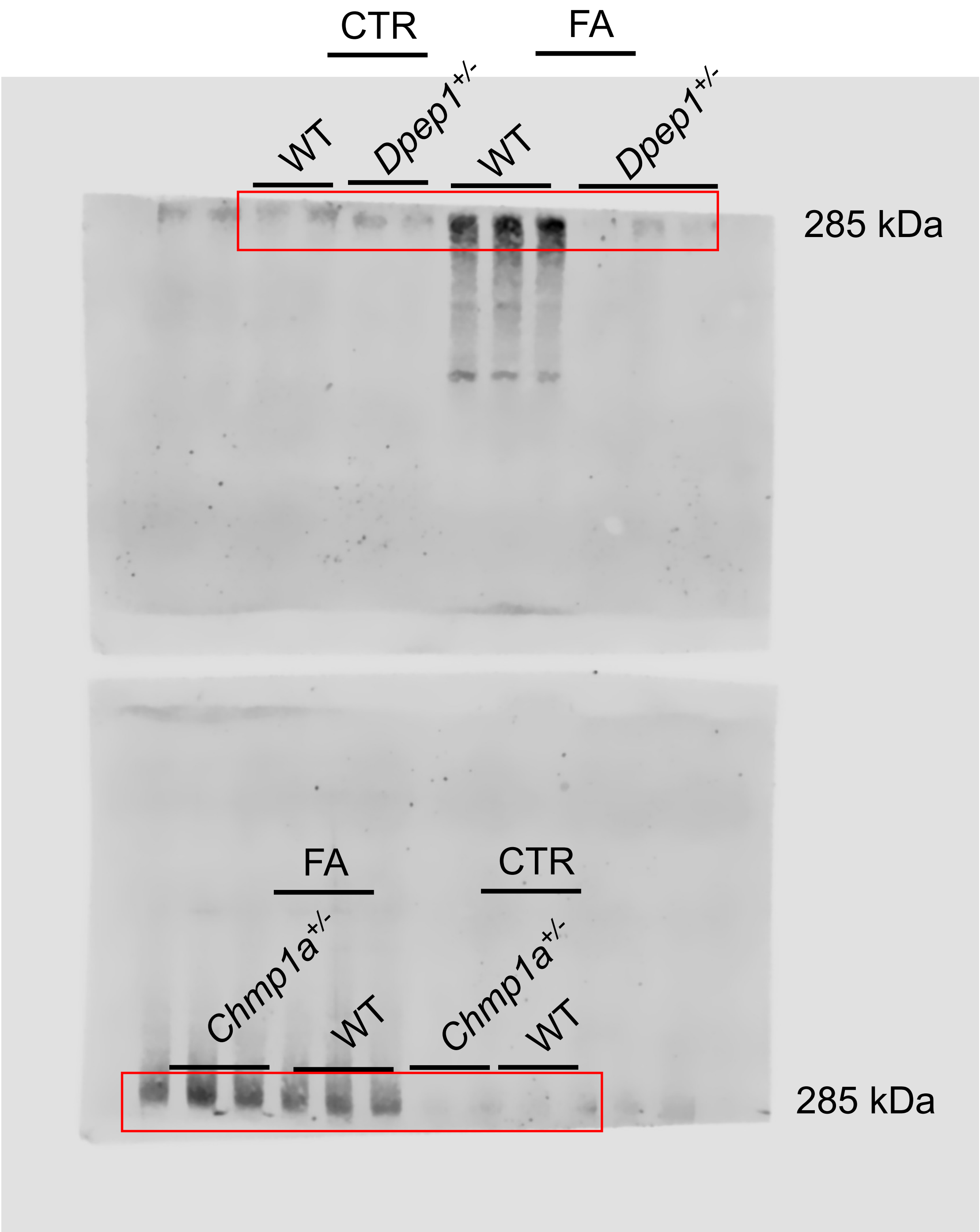

Figure 3l COL

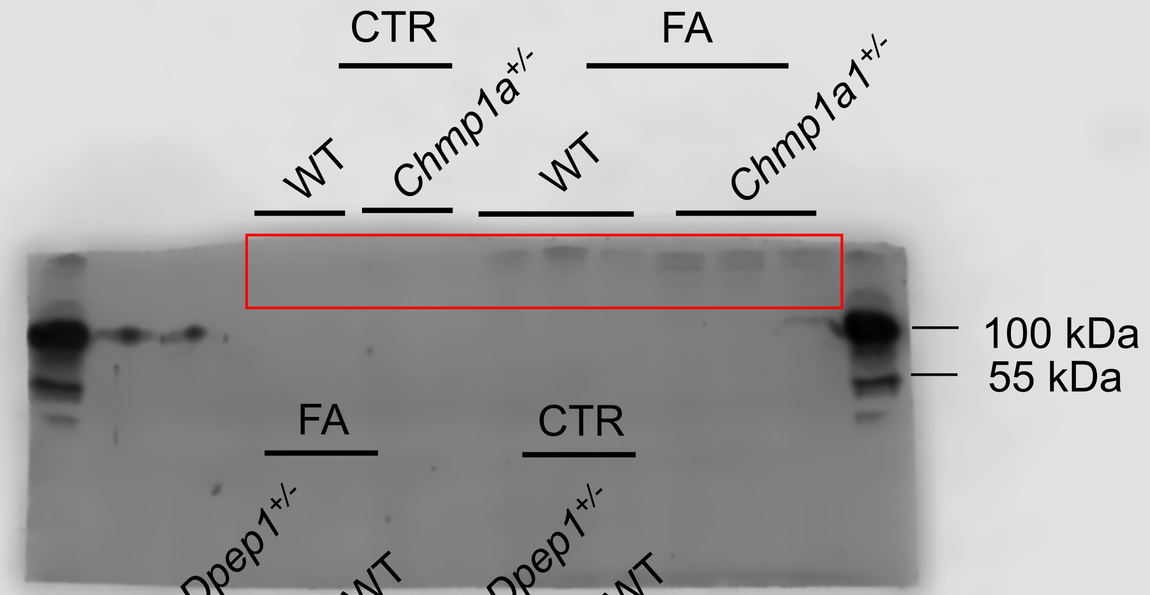

Figure 3f COL

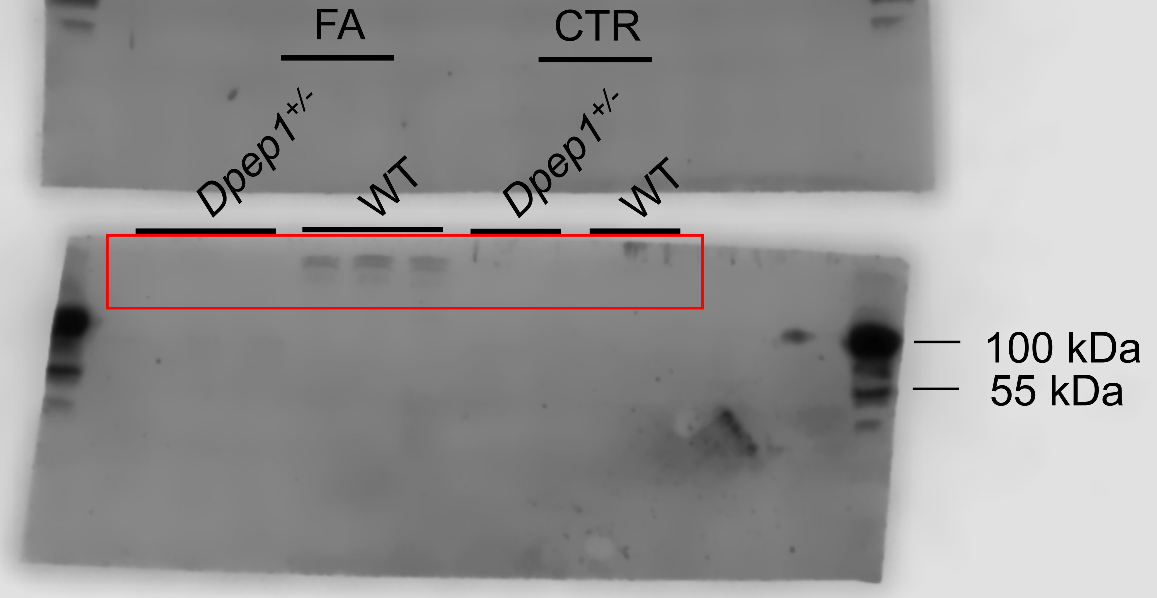

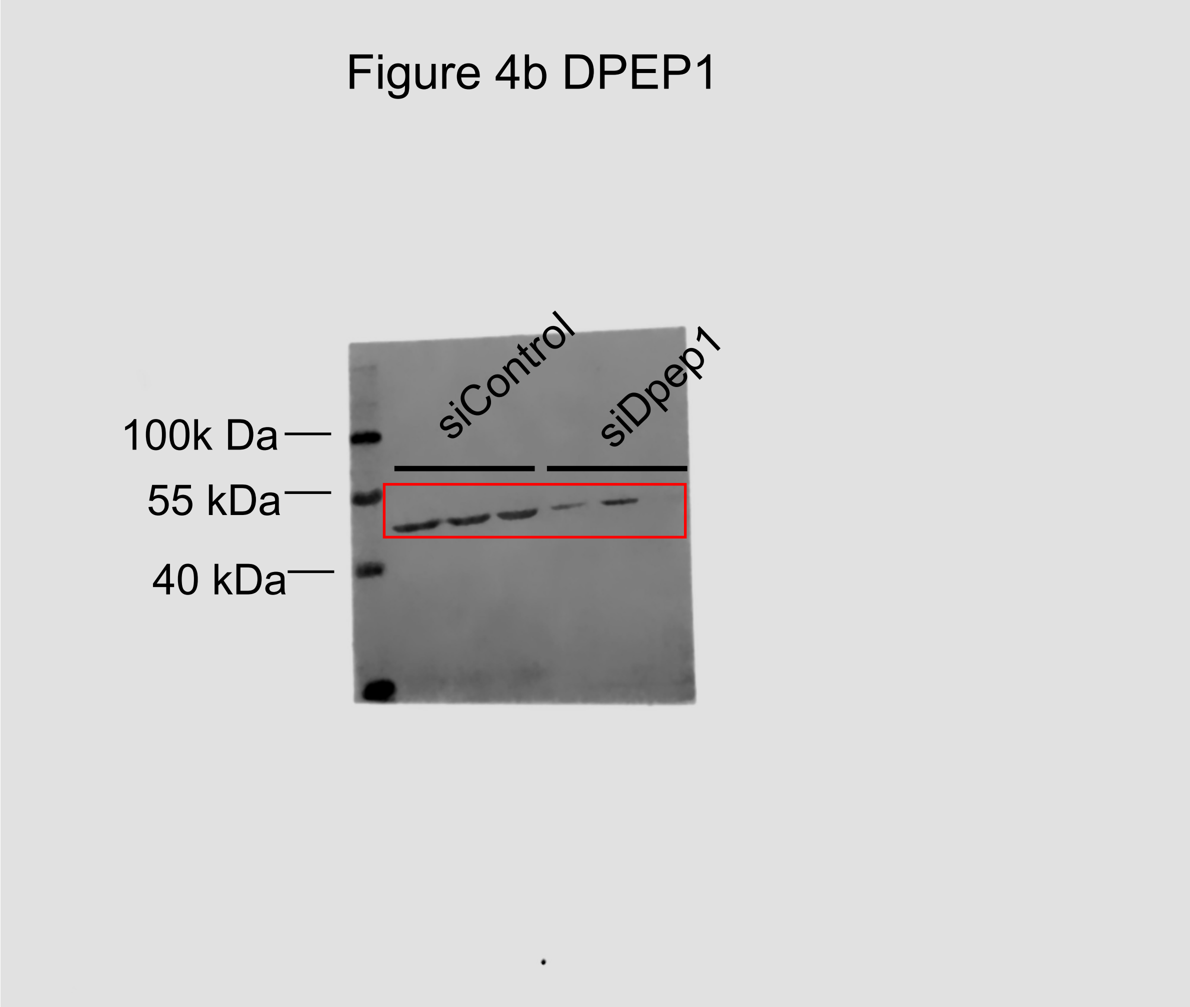
Figure 4b DPEP1
siControl
siDpep1
100k Da
55 kDa
40 kDa

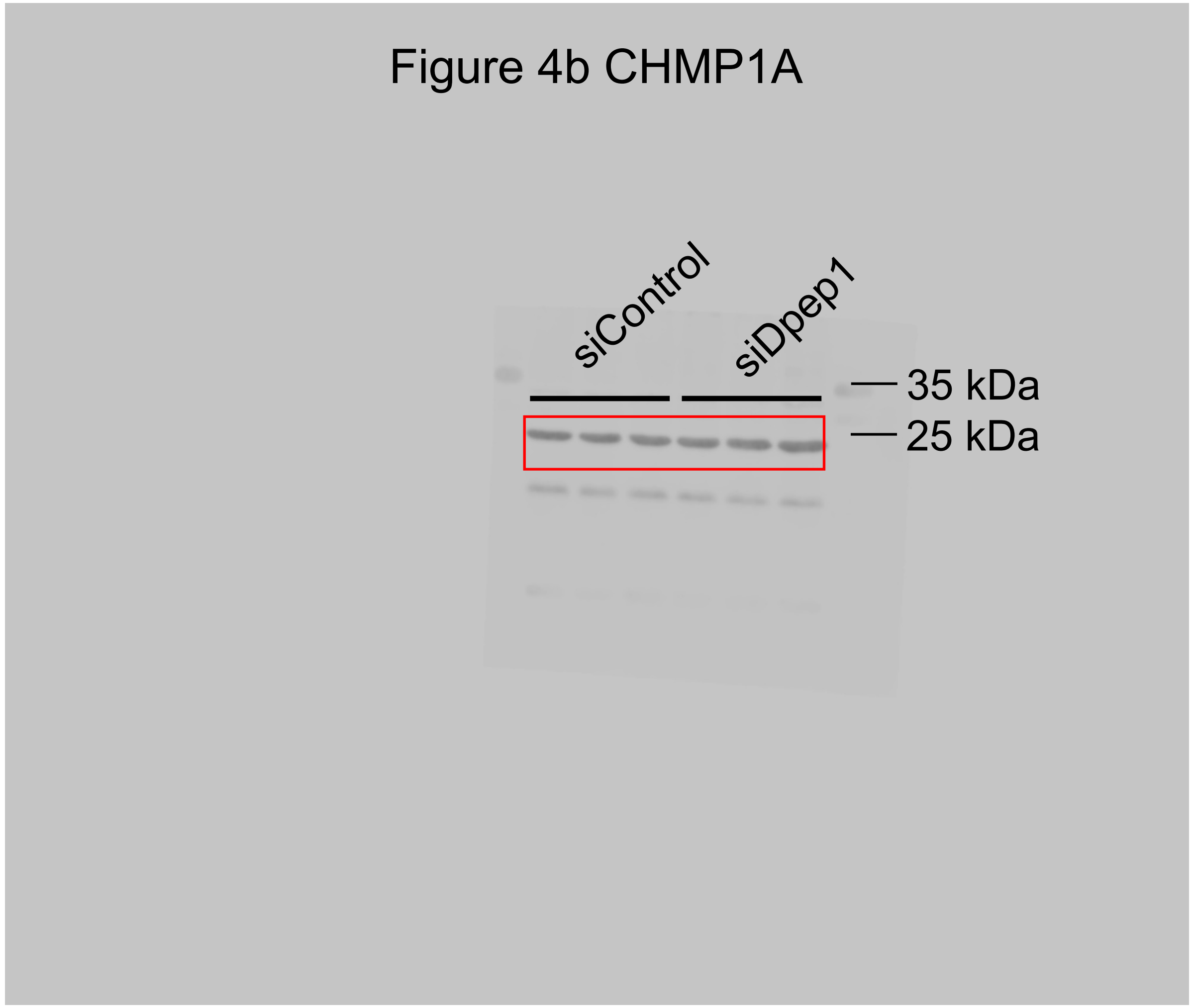
Figure 4b CHMP1A
siControl
siDpep1
35 kDa
25 kDa

Figure 4b TUBULIN

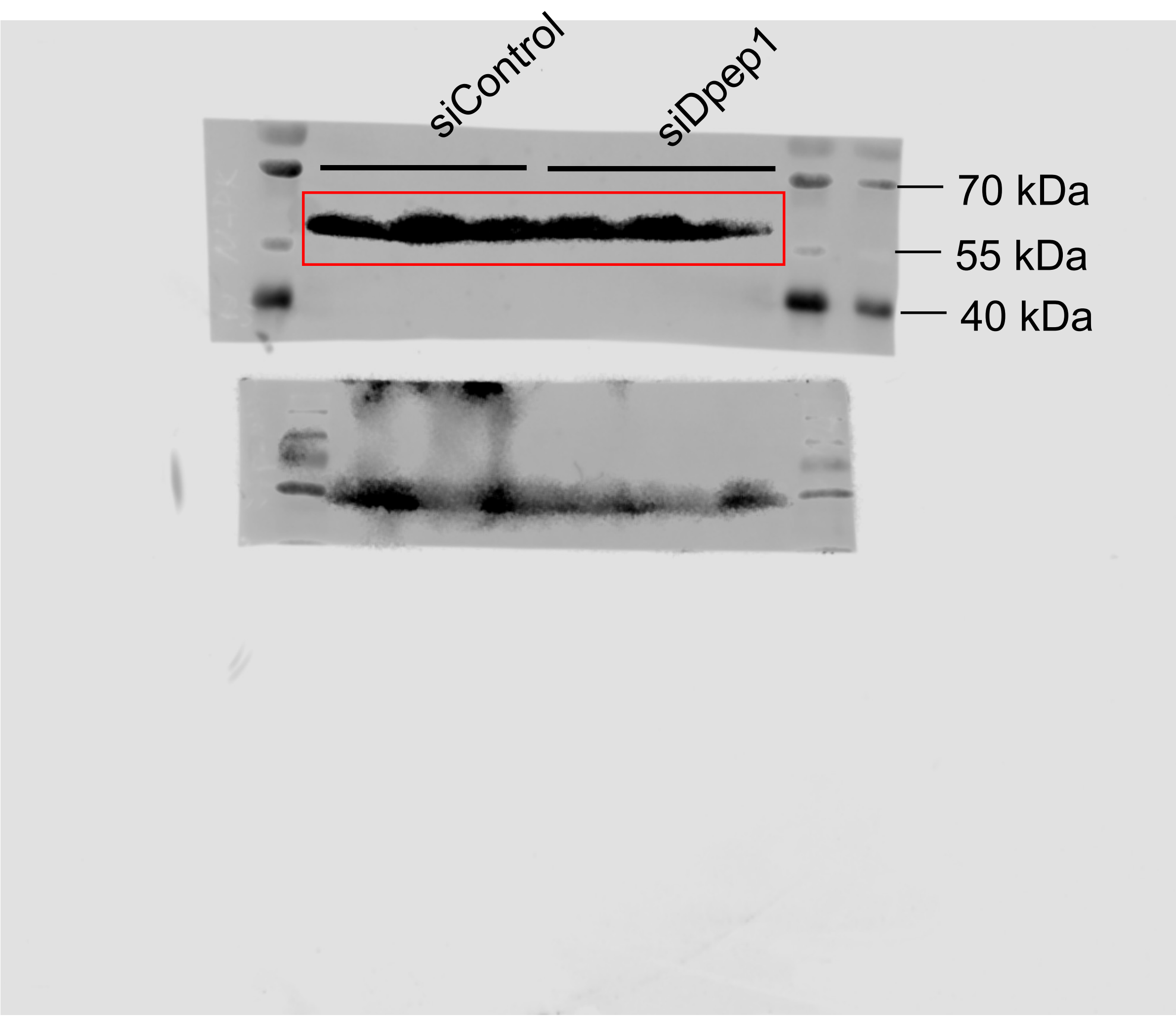

Figure 4l RIP3

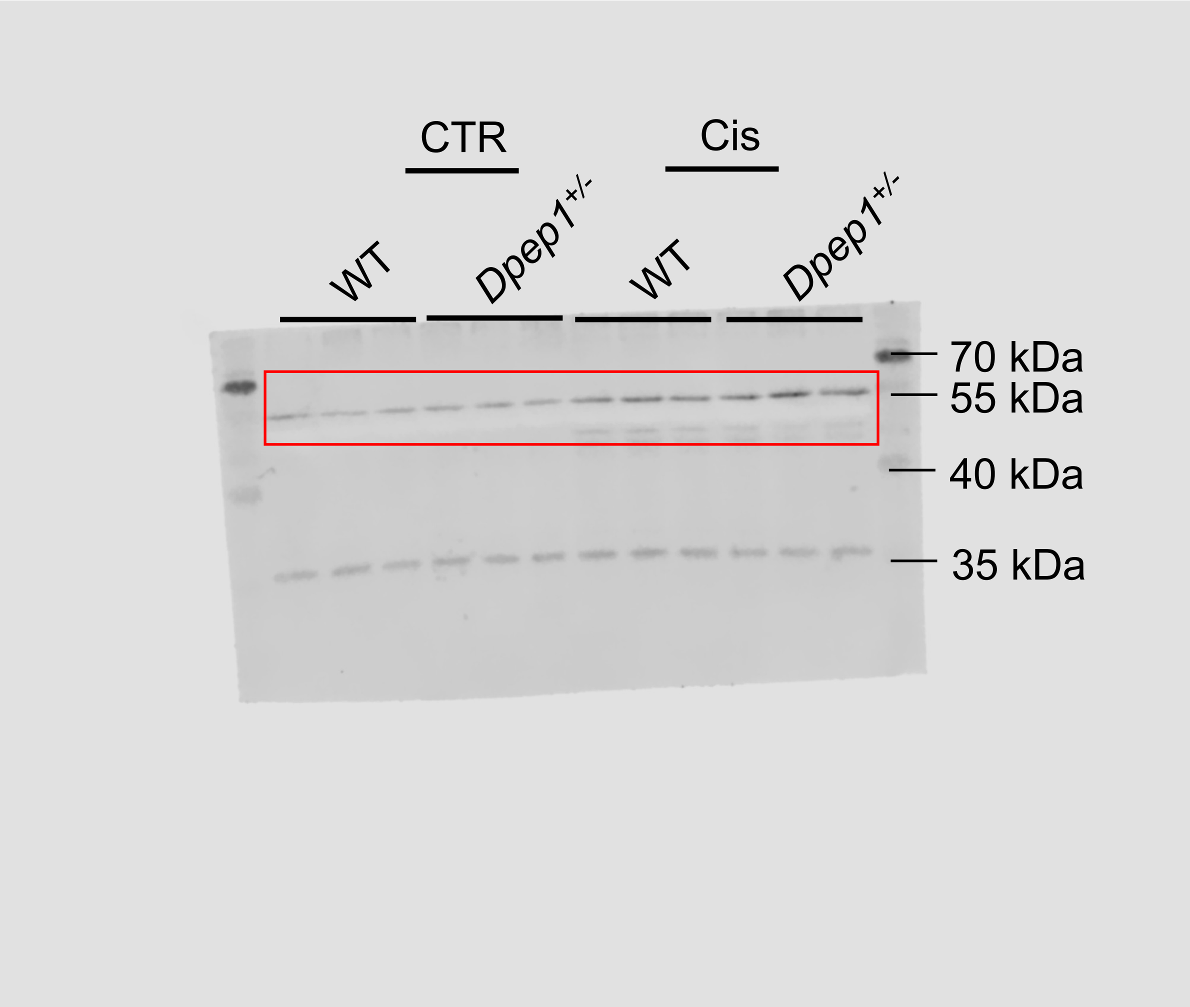

Figure 4I cleCaspase1

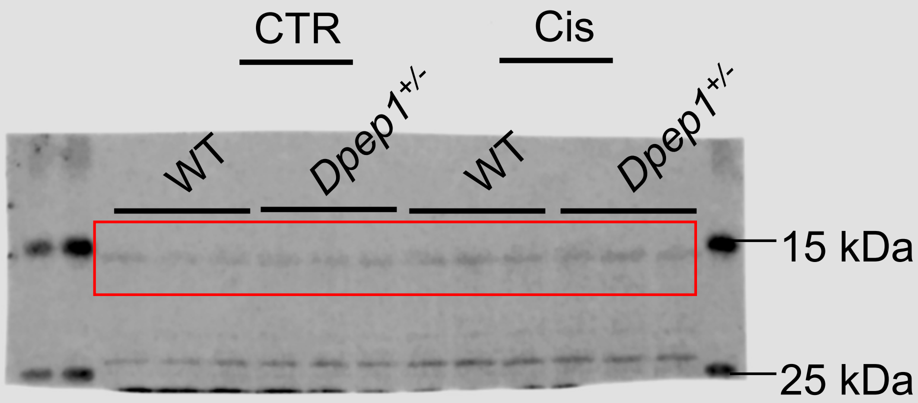

Figure 4I actin

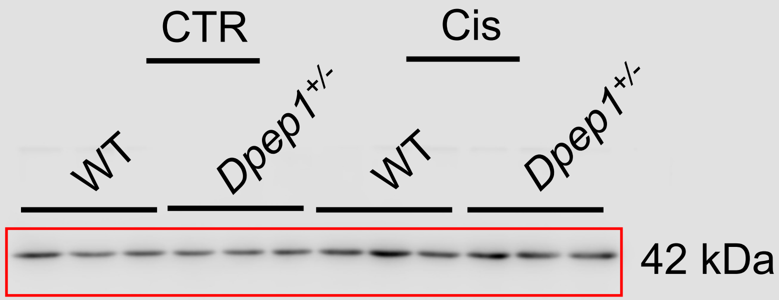

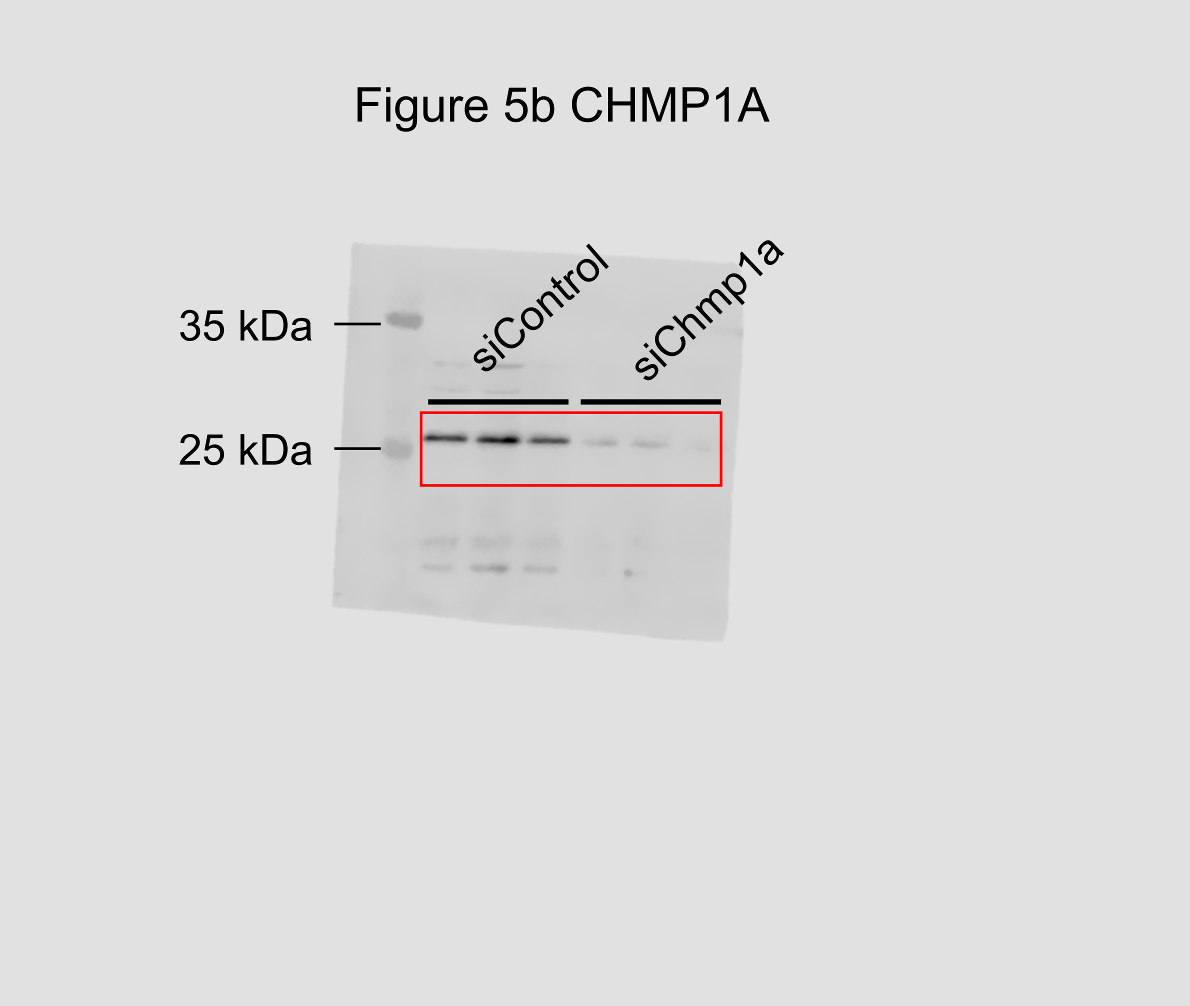
Figure 5b CHMP1A
35 kDa
25 kDa
siControl
siChmp1a

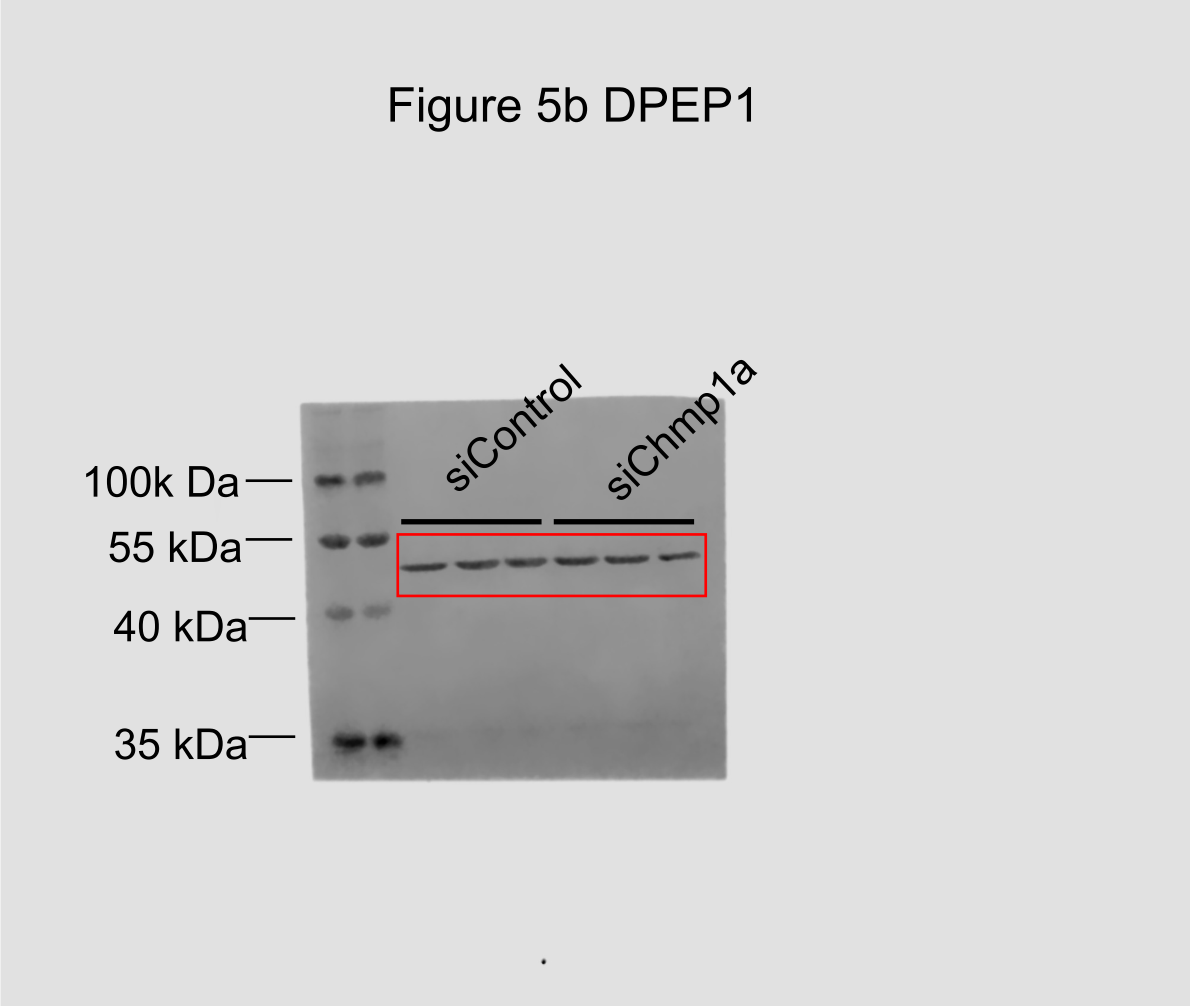
Figure 5b DPEP1
100k Da
55 kDa
40 kDa
35 kDa
siControl
siChmp1a

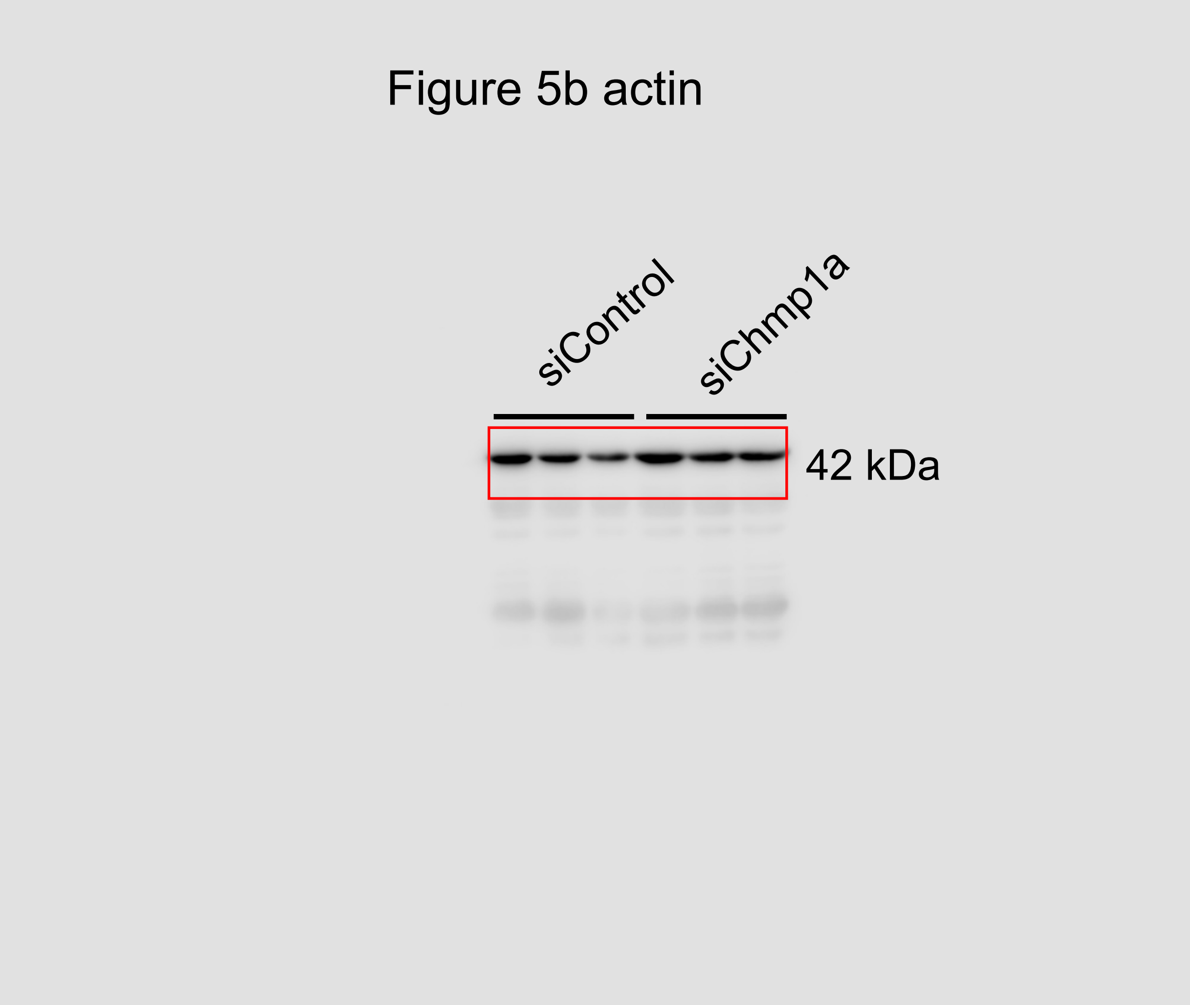
Figure 5b actin
siControl
siChmp1a
42 kDa

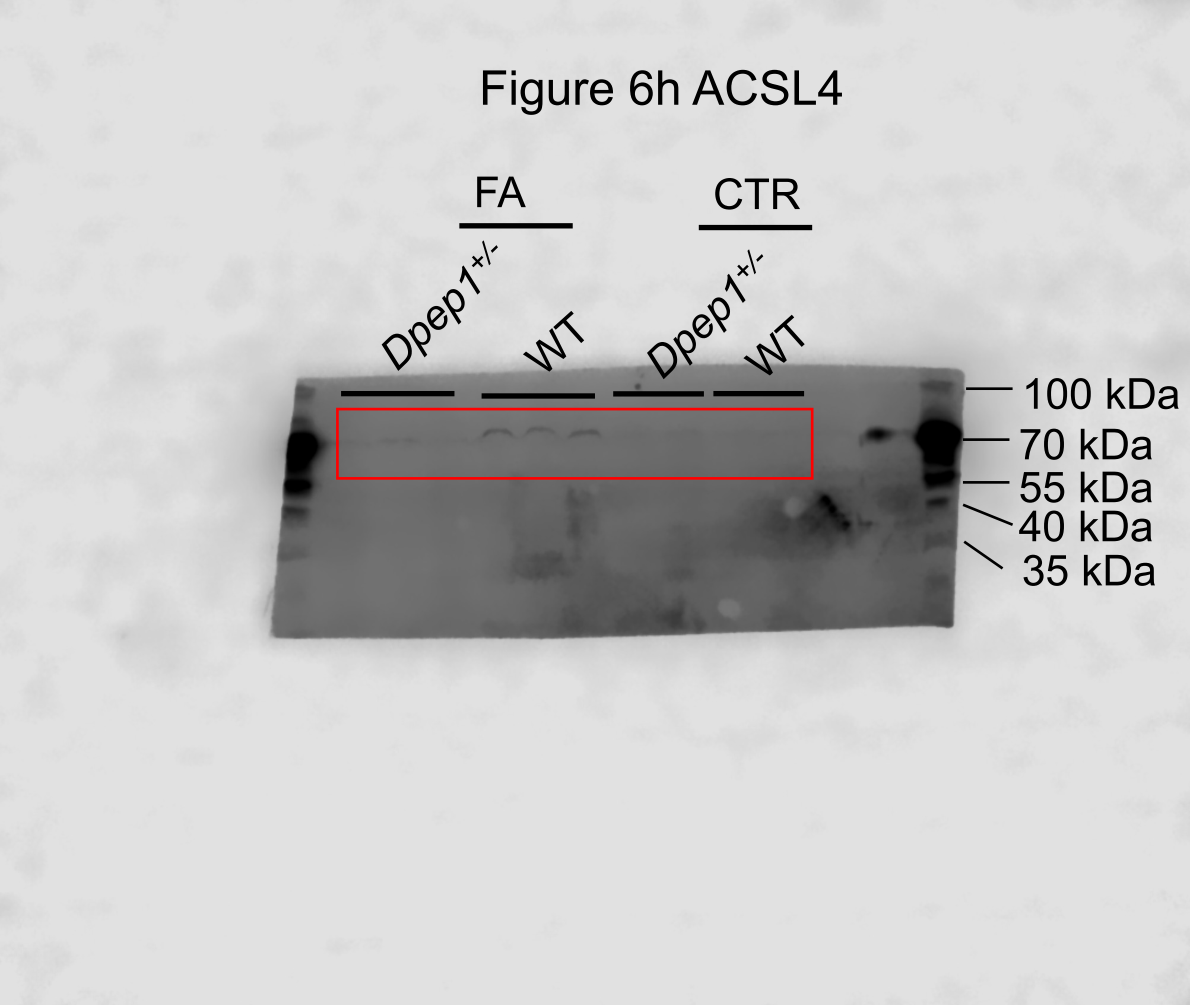
Figure 6h ACSL4
FA
CTR
Dpep1+/-
WT
Dpep1+/-
WT
100 kDa
70 kDa
55 kDa
40 kDa
35 kDa

Figure 6h TUBULIN

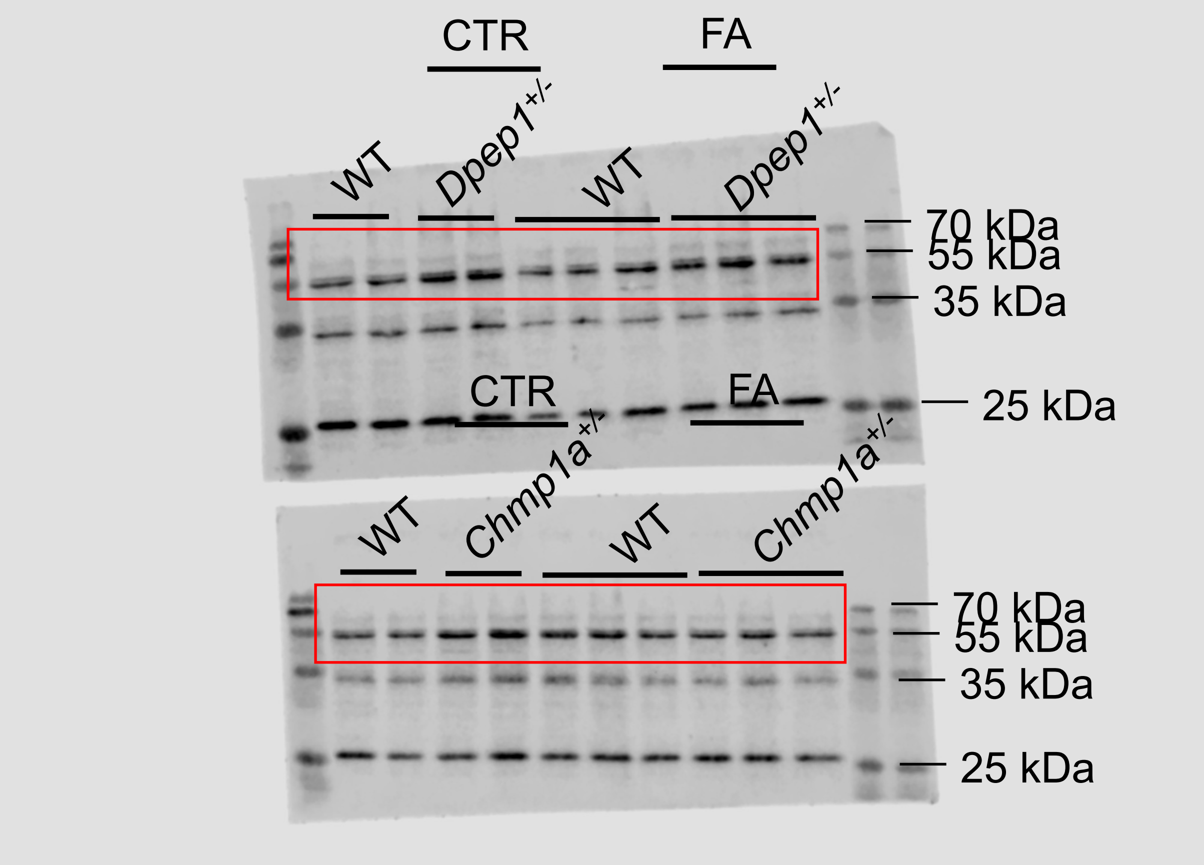

Figure 7c TUBULIN

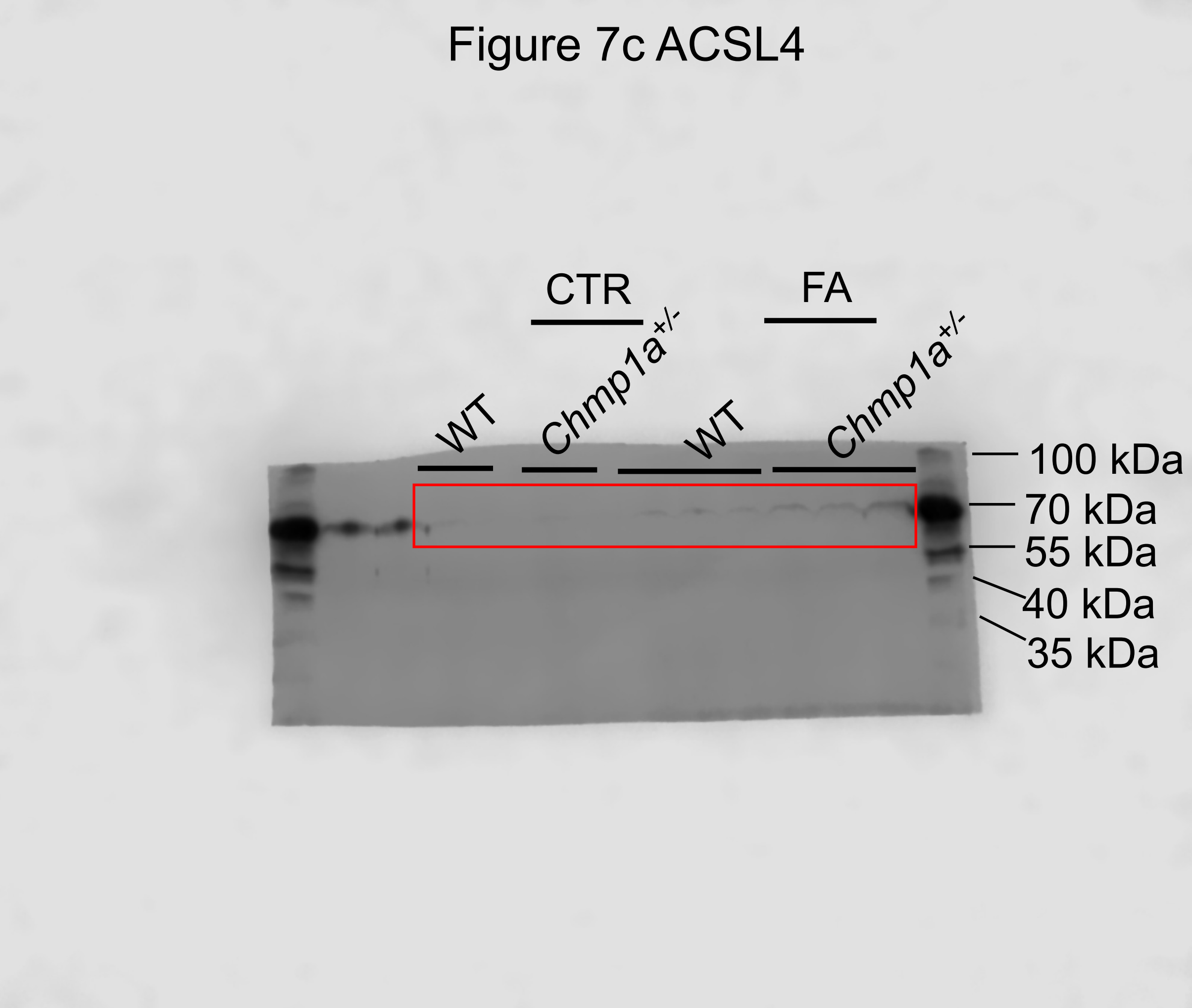

Figure 7f ACTIN

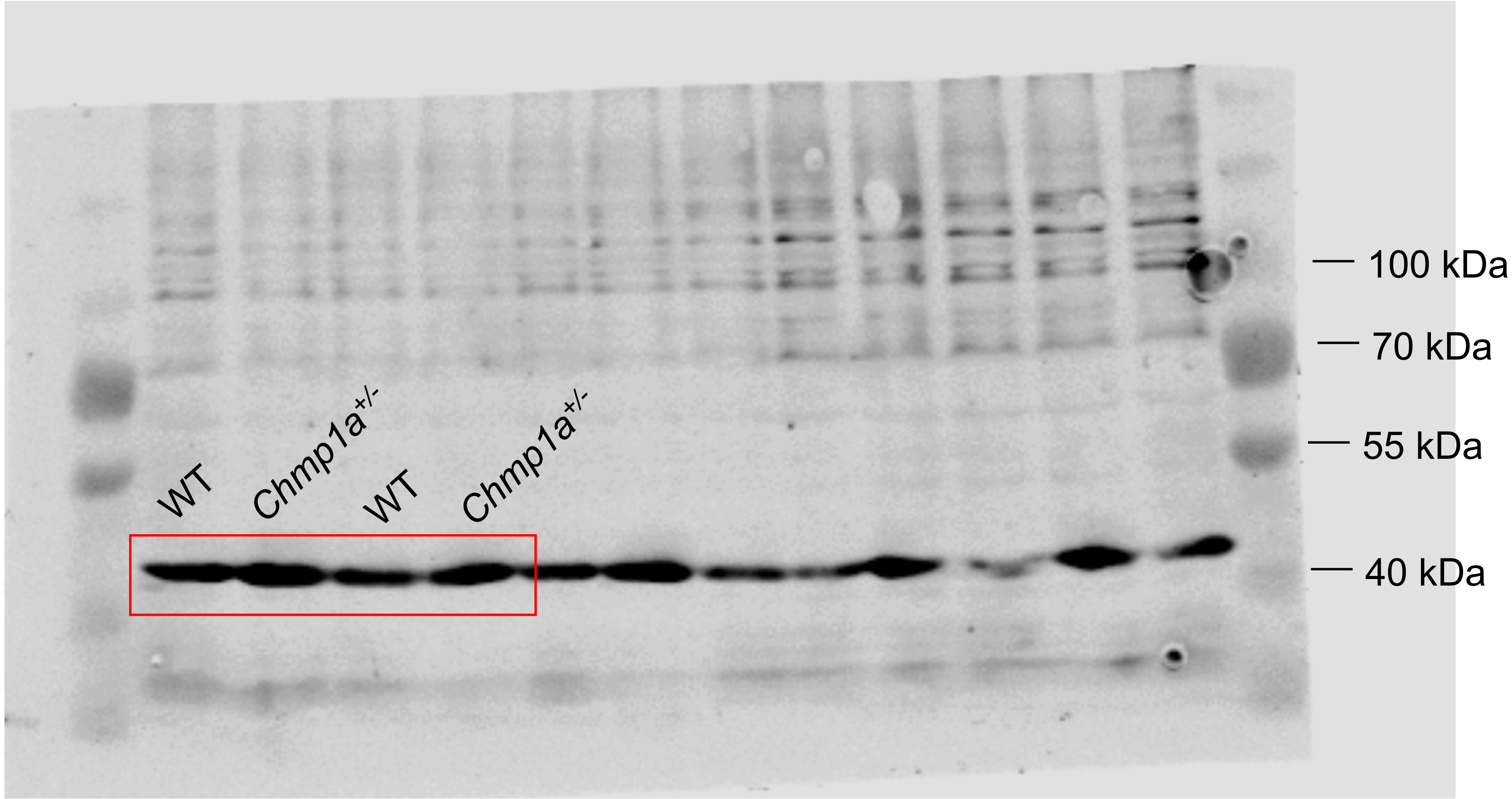

Figure 7f CD63

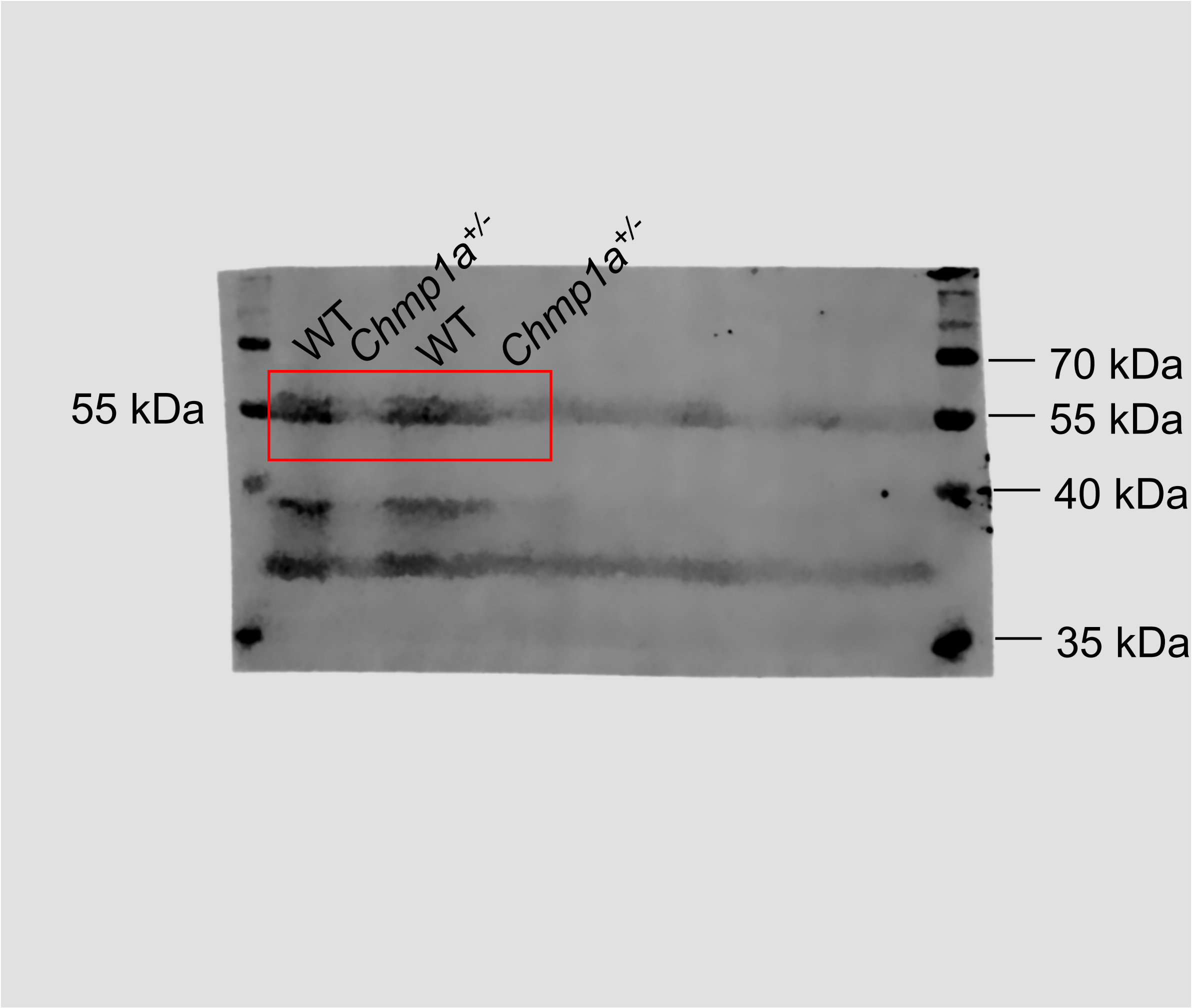

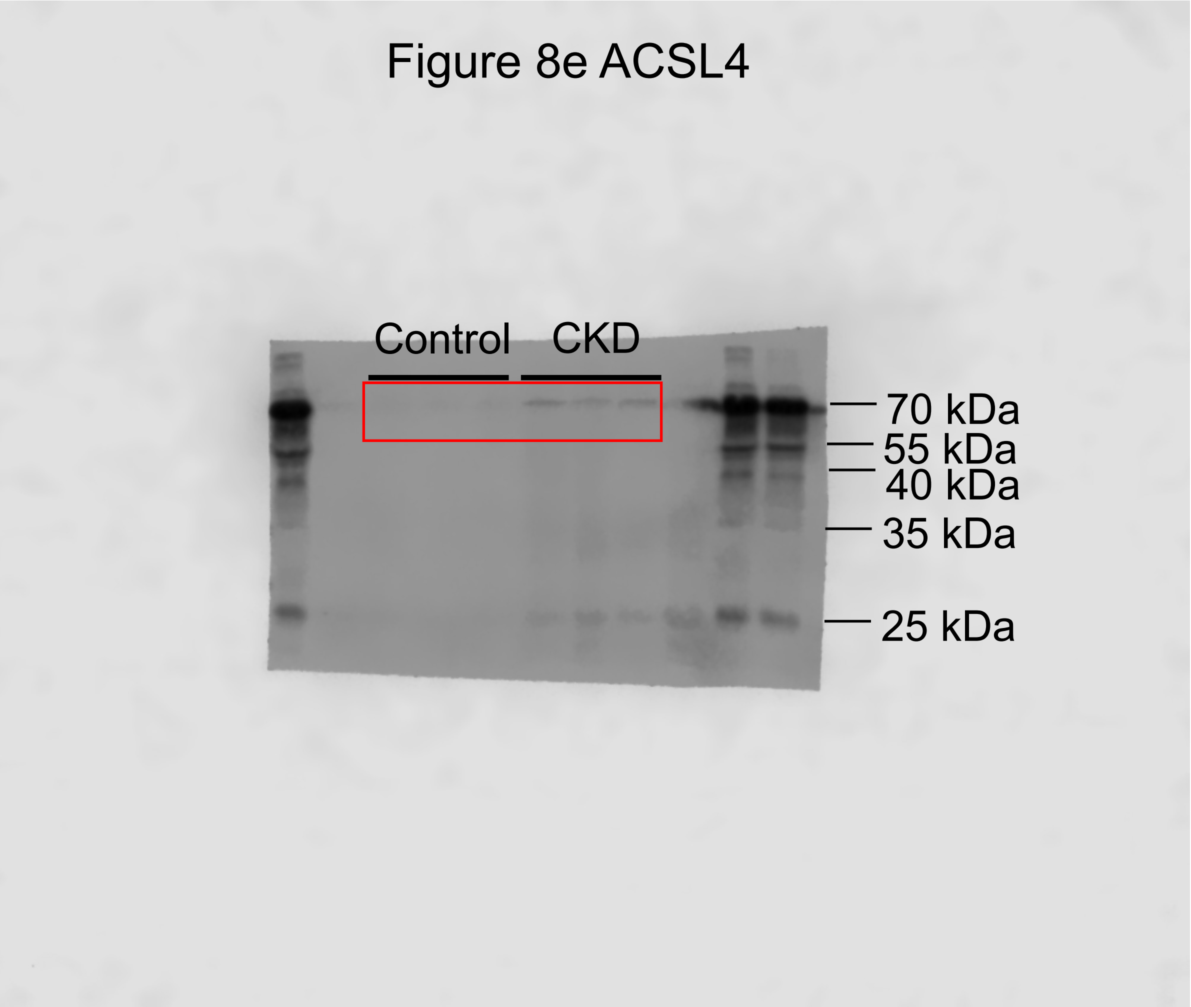
Figure 8e ACSL4
Control
CKD
70 kDa
55 kDa
40 kDa
35 kDa
25 kDa

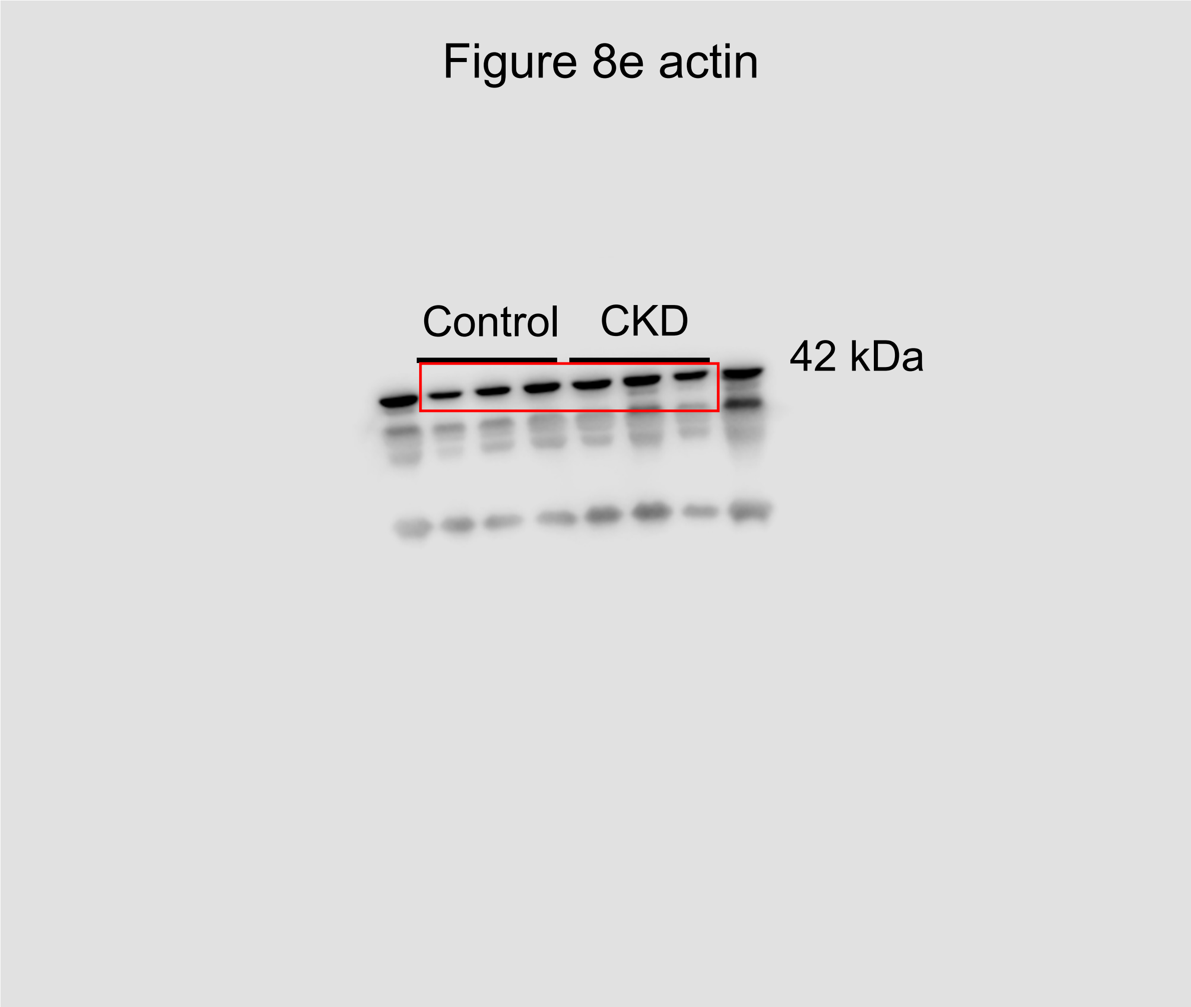
Figure 8e actin
Control
CKD
42 kDa

Figure S4e Dpep1

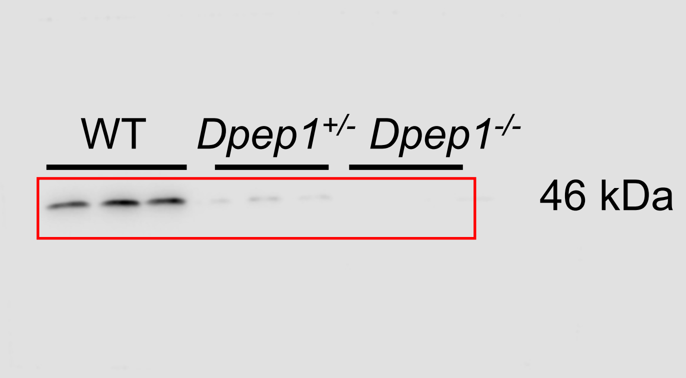

Figure S4e actin

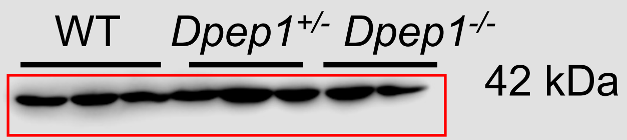

Figure S5b CHMP1A

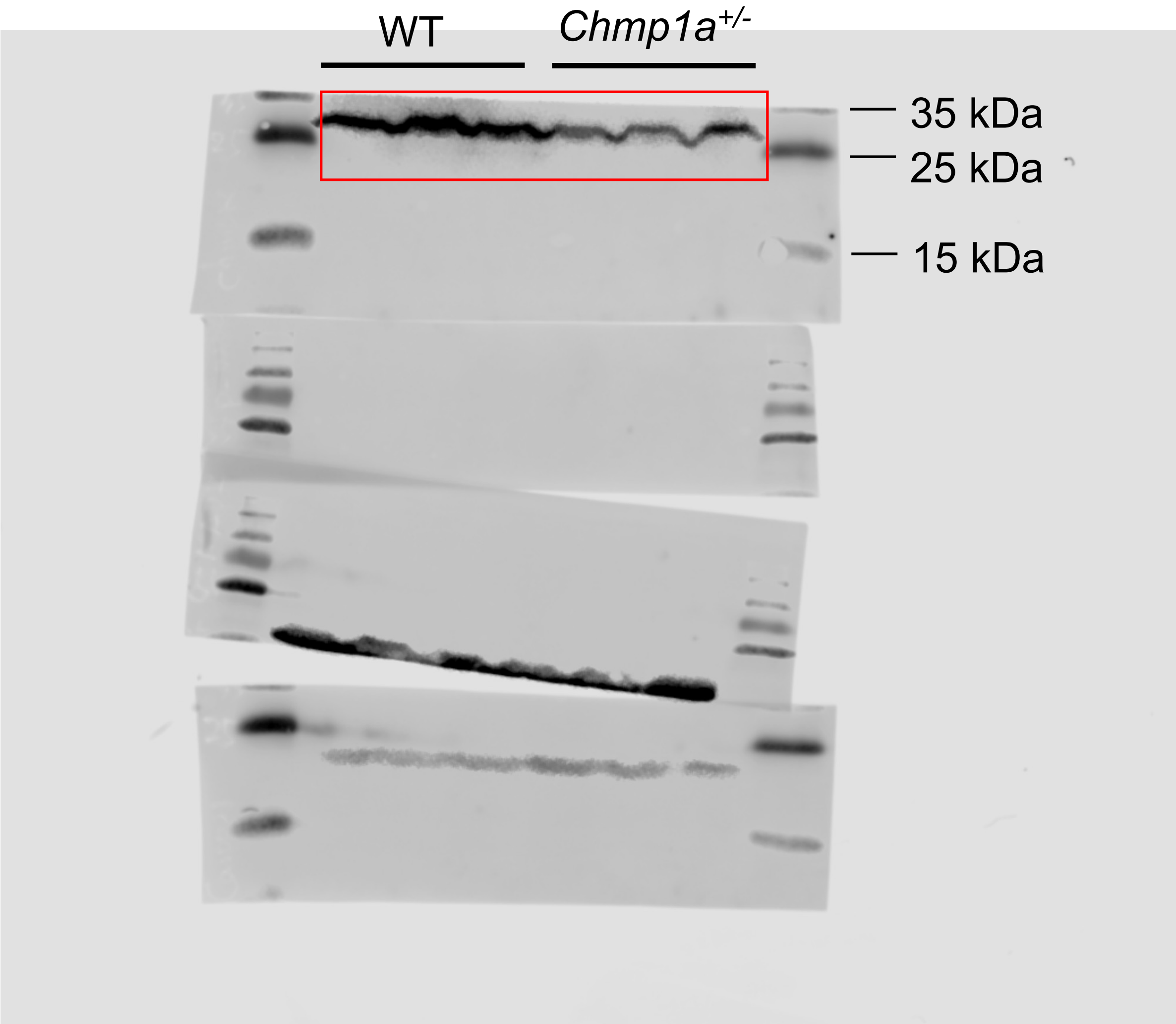

Figure S5b TUBULIN

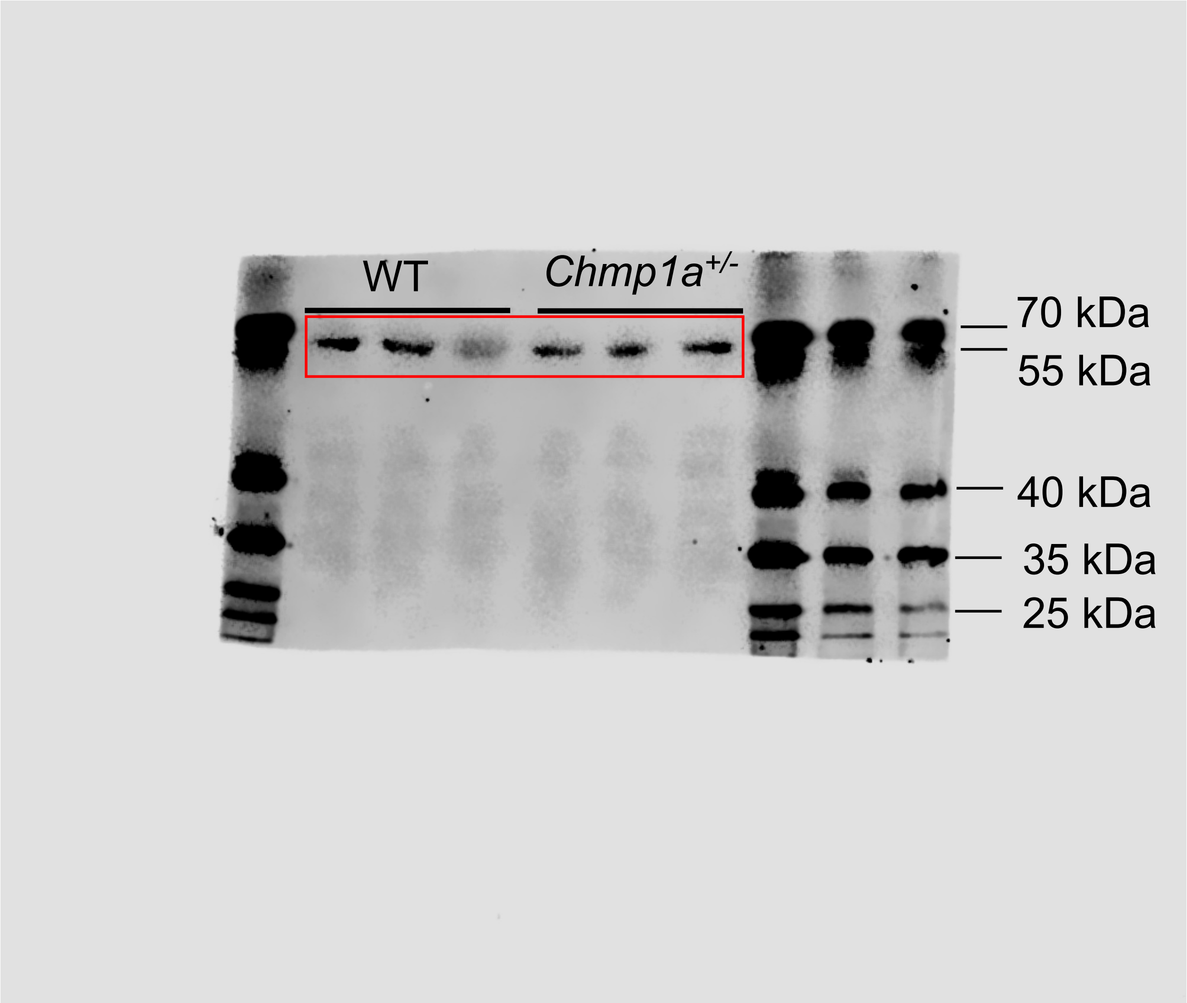

Figure S10c GPX4

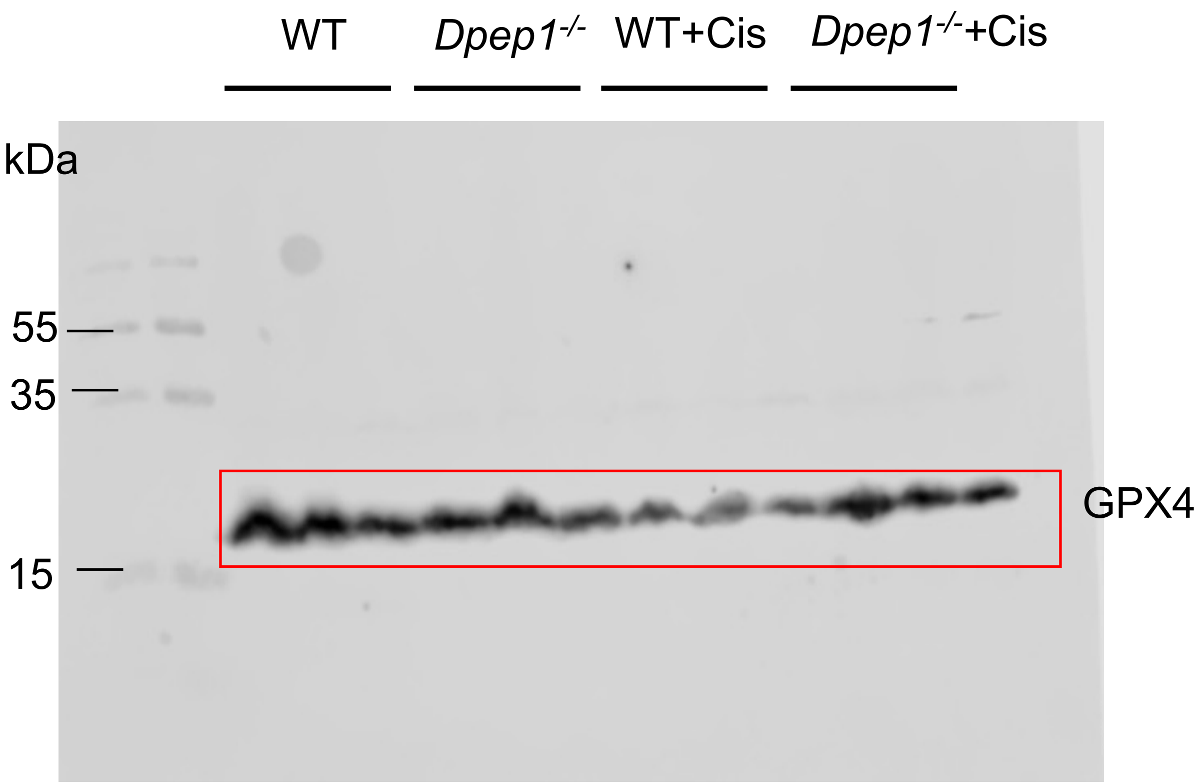

Figure S10c Actin

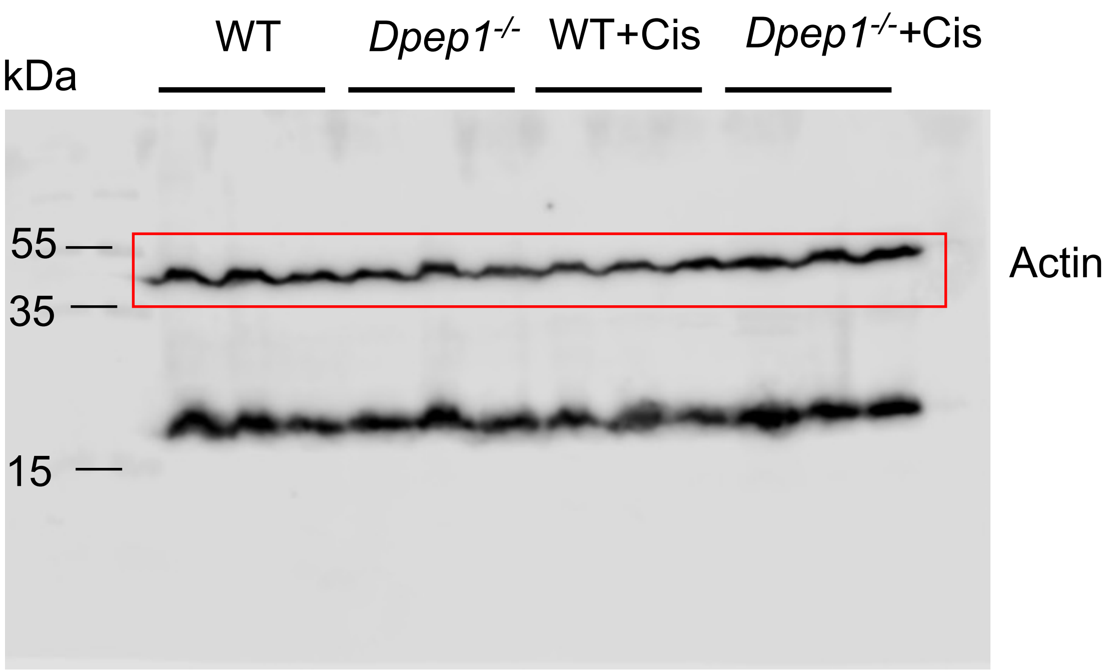

Supplement: Supplementary file 5 — Source Data [file 41467_2021_25377_MOESM5_ESM.zip › Source Data/Western blots.pdf]
